# Beckmann rearrangement of ketoximes for accessing amides and lactams promoted by a perimidine-2-thione supported Hg(ii) complex: a mechanistic perception†

**DOI:** 10.1039/d5ra02843d

**Published:** 2025-07-11

**Authors:** Priyanka Velmurugan, Poovarasan Kanniyappan, Tapas Ghatak

**Affiliations:** a Advanced Catalysis Facility, Department of Chemistry, School of Advanced Sciences, Vellore Institute of Technology Vellore-632014 Tamil Nadu India tapaschem@gmail.com tapas.ghatak@vit.ac.in

## Abstract

In this study, we present a new distorted tetrahedral mononuclear mercury(ii) complex mediated by a 1-isopropyl-1*H*-perimidine-2(3*H*)-thione ligand, with a molecular formula C_28_H_28_Cl_2_HgN_4_S_2_. Several characterization techniques, such as single-crystal X-ray diffraction, NMR, and FT-IR, were employed to analyze the complex. The synthesized complex acted as a metallic Lewis acid catalyst for the conversion of a wide array of ketoximes into amides and lactams, resulting in an expansion of the Beckmann rearrangement. Different types of substrates were successfully converted into corresponding amides under mild reaction conditions.

## Abstract 2

A newly synthesized Hg(ii)–perimidine-2-thione complex catalyzes the Beckmann rearrangement across a broad range of oximes, enabling high-yield access to amides and lactams.

## Graphical abstract

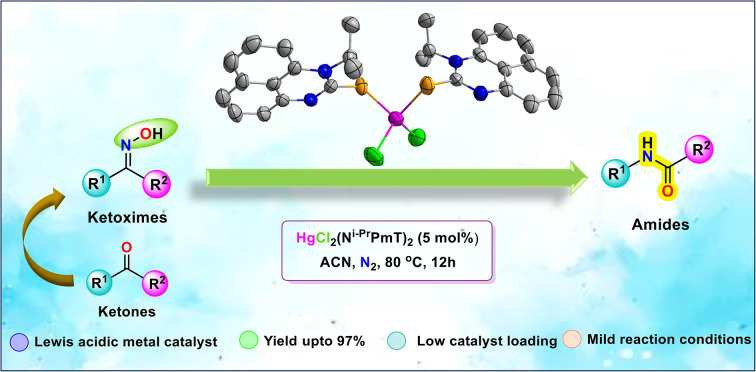

## 1 Introduction

**Scheme 1:**
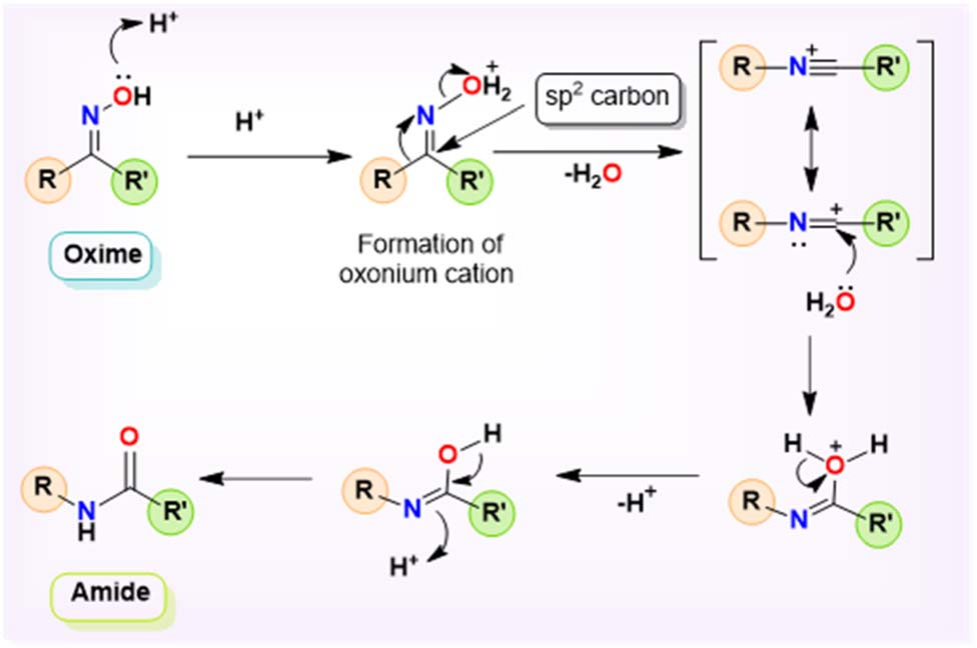
General mechanism of Beckmann rearrangement.

Beckmann rearrangement (BKR) is recognized as a viable alternative pathway for the synthesis of amides and lactams.^1–3^ In 1886, the German chemist Ernst Otto Beckmann devised the BKR as an alternative method for synthesizing amides and lactams using the easily accessible oxime precursors.^4,5^ Specifically, the conversion of a ketoxime to its corresponding amide, known as the Beckmann rearrangement, is a widely acknowledged method in organic chemistry and remains an area of continual study.^6–10^ The topical and vital role of BKR in several manufacturing industries is seen in the industrial preparations of paracetamol and *ε*-caprolactam, which are intermediates in the production of nylon-6,6.^11–13^ Continuing along this line, it is notable that secondary amides are of particular relevance due to their prevalence as the primary structural component in several natural products, agrochemicals, medicines, detergents, lubricants, and functional materials.^10,14–17^ Furthermore, even from an atom-economy perspective, BKR is particularly appealing because of the affordable access to the matching ketoximes as starting materials, which is made possible by the extensive availability of structurally diverse and affordable ketones.^18,19^ An appropriate departing oxonium cation is produced *via* the first protonation at the ketoxime oxygen in BKR, which causes the hydroxyl group to leave and a substituent (an alkyl or aryl fragment, opposite the leaving group) to migrate from the sp^2^ carbon atom to the nitrogen cation (Scheme 1).^20^ The concurrent cleavage of the C–C bond and the formation of a new C–N bond represent the most direct and dependable method for incorporating the nitrogen atom into linear, branched, and cyclic ketones, resulting in the formation of an amide bond.^10^ Owing to its capacity to enable the cleavage of a C–C bond while simultaneously facilitating the formation of a C–N bond, BKR has attracted considerable interest in recent years. Amides are a significant class of functional groups in the fields of organic and biological chemistry.^21^ Amide functionality is abundant in pharmaceuticals and drugs, natural products, and a broad range of industrial materials, such as polymers, detergents, and lubricants.^22,23^ Therefore, the synthesis of amides is recognized as a significant chemical transformation in organic chemistry. Several methods using various precursors may be used to synthesize amides; these include the acylation of amines,^24^ Staudinger ligation process,^25^ Schmidt reaction,^26^ and BKR, among others.^27–31^

Nevertheless, the typical Beckmann reaction is associated with significant limitations, including harsh reaction conditions, yields that range from low to moderate, generation of numerous byproducts, and low compatibility with acid-sensitive substrates.^32,33^ Recent years have seen the development of several intriguing variants that address these deficiencies by activating the oxime hydroxy group.^34–36^ Consequently, significant endeavors have been undertaken over the past few decades to navigate the challenging conditions; a range of methodologies in the liquid phase,^37^ vapor phase,^38^ supercritical water,^39^ and ionic liquids^40^ have been established. In the liquid phase, ferric chloride-impregnated silica gel, referred to as the silferc catalyst, along with ferric chloride-impregnated montmorillonite K10, were employed as catalysts for the Beckmann rearrangement.^41,42^ Many “improved” approaches to BKR have been published in recent years, including gas-phase reactions,^43–45^ organic small-molecule catalysis,^46–53^ transition metal catalysis,^54^ Brønsted or Lewis acid catalysts,^55^ photocatalytic systems,^56,57^ and a noncatalytic Beckmann rearrangement for caprolactam synthesis that is triggered by supercritical water.^58,59^ Even though several reagents have been developed thus far for BKR, they have several economic and environmental consequences. In recent years, metal-catalyzed Beckmann rearrangement has surfaced as a promising approach for facilitating amide conversions.

**Scheme 2:**
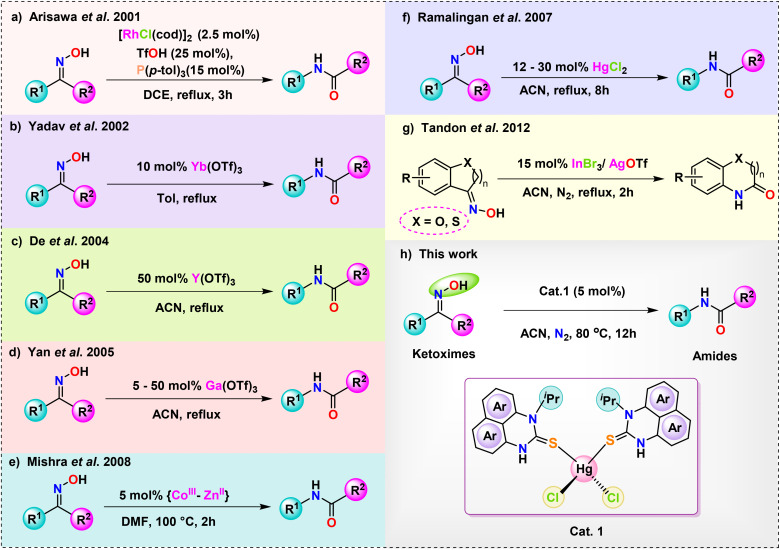
Schematic representation of different Lewis acid-based metal catalyst-promoted Beckmann rearrangement of oximes to amides.

Arisawa *et al.* reported that a rhodium complex, together with triflic acid and a tri-aryl substituted phosphine ligand, potentially catalyzes Beckmann rearrangement (Scheme 2a).^60^ The issue of how to transform cyclic ketoximes into lactams remains unanswered, despite the fact that this new strategy effectively catalyses acyclic ketoximes. Many metal triflate salts, including Yb(OTf)_3_,^61^ Y(OTf)_3_,^62^ Ga(OTf)_3_,^63^ and *in situ* produced In(OTf)_3_ from InBr_3_/AgOTf,^64^ are suitable catalysts for Beckmann rearrangement because of their Lewis acidic character (Scheme 2b–d and g). Additionally, Mishra *et al.* have examined the catalytic application of a heterobimetallic complex [Co(iii)–Zn(ii)] towards Beckmann rearrangement (Scheme 2e).^65^ Despite extensive substrate research, the reactivity of cyclic ketoximes has so far eluded examination, and very low yields of the compounds severely restrict its potential applications.

In 2007, Ramalingan *et al.* implemented a HgCl_2_ metal halide to catalyze the Beckmann rearrangement of ketoximes into amides and lactams (Scheme 2f).^66^ In recent years, numerous mercury-based catalytic systems have been employed in organic synthesis to facilitate selective and essential transformations.^67–70^ Investigating the potential catalytic applications of transition metal-perimidin-2-thione complexes in a range of organic transformations is the current focus of our lab. The exceptional efficacy of the freshly synthesized Hg(ii)-perimidine-2-thione complex in Beckmann Rearrangement, a process that transforms ketoximes into amides, became apparent upon careful analysis of its catalytic characteristics in relation to several chemical processes. This report elucidates the application of metallic Lewis acid catalysts, specifically HgCl_2_(N^i-Pr^PmT)_2_ (1), (N^i-Pr^PmT = 1-isopropyl-1*H*-perimidin-2(3*H*)-thione/*N*-isopropyl-1*H*-perimidin-2(3*H*)-thione), alongside the implementation of mild reaction conditions, including moderate temperature, which collectively augment the yield of amides and lactams in the conventional Beckmann rearrangement reaction (Scheme 2h). Using a perimidin-2-thione ligand as a catalyst, this seems to be the first instance of a mercury(ii) metal complex system that enables Beckmann rearrangement.

## 2 Results and discussion

### 2.1. Synthesis of mercury(ii) complex (1)

**Scheme 3:**
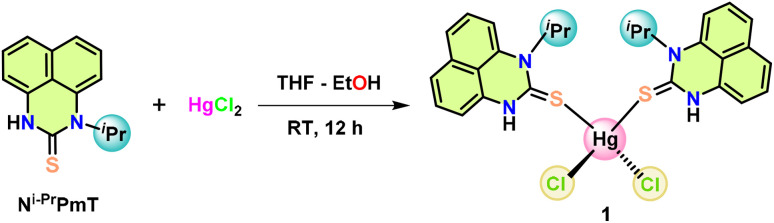
Synthesis of mercury complex 1.

**Fig. 1:**
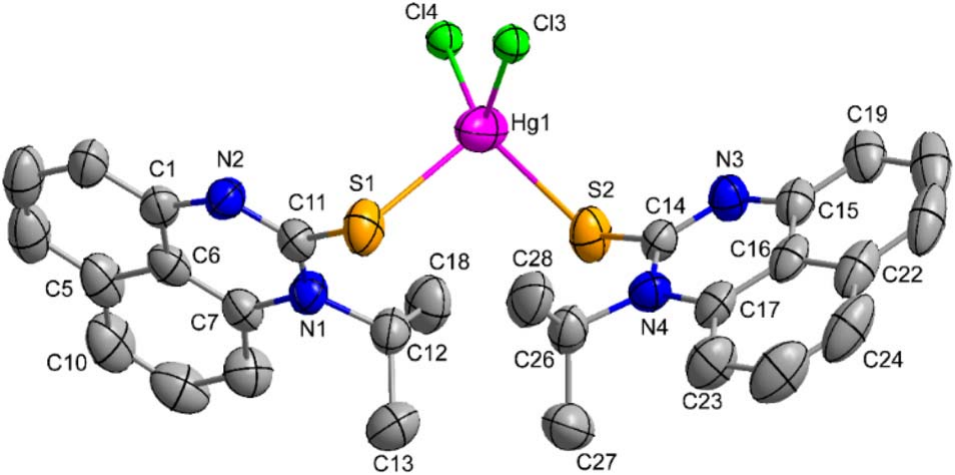
Molecular structure of mercury(ii) complex (1). Thermal ellipsoids are at a 50% probability level. Hydrogen atoms and solvent molecules are omitted for clarity. Selected bond distances (Å): Hg1–Cl4 2.443(3); Hg1–Cl3 2.448(3); Hg1–S2 2.540(2); Hg1–S1 2.596(2). Bond angles (°): Cl4–Hg1–Cl3 112.76(10); Cl3–Hg1–S2 117.10(9); Cl4–Hg1–S1 113.61(9); S2–Hg1–S1 104.16(7).

The synthesis of the Hg(ii) complex involved the careful addition of a THF solution containing the N^i-Pr^PmT ligand to an ethanolic solution of mercuric chloride, resulting in the formation of a dark green precipitate. The mixture was stirred overnight at ambient temperature to yield the mercury(ii) complex (1) (Scheme 3). The dark yellow crystals suitable for single crystal X-ray diffraction studies were obtained by concentrating its toluene solution at −20 °C. The crystal structure of complex 1 is depicted in Fig. 1.

### 2.2. Infrared spectra and NMR

Fig. S55† presents the stacked FTIR spectra of complex 1 and the N^i-Pr^PmT ligand. In the complex, the N–H stretching vibration appears at 3150 cm^−1^, indicating a slight red shift relative to the free ligand, which displays this band at 3159 cm^−1^. This shift suggests that coordination to the metal center perturbs the N–H bond, potentially due to its involvement in hydrogen bonding interactions with chloride ligands, resulting in a weakened N–H bond.^71^ Complex 1 displayed a distinct absorption band at 1162 cm^−1^, which is attributed to the C=S stretching vibration mode. The *ν*(C–H) stretching vibrations for the unsaturated and saturated bonds in the permidine-2-thione ligands are detected at 3010 cm^−1^ and 2989 cm^−1^, respectively.^72^ The band at 1659 cm^−1^ corresponds to the characteristic absorption of the C–N bond of the perimidine ring core in complex 1. The absorption bands observed in the 1400–1600 cm^−1^ region are attributed to the *ν*(C–C) and *ν*(C–N) stretching vibrations of the perimidine ring, reflecting the characteristic vibrational frequencies of the ring system.

In the ^1^H NMR spectrum of the Hg(ii) complex 1, the –NH proton of the two perimidine-2-thione ligands appears as a sharp singlet at *δ* 11.50 ppm, whereas in the free ligand, this signal is observed at *δ* 9.04 ppm.^71^ The pronounced downfield shift of the –NH resonance upon complexation suggests deshielding of the proton, likely due to coordination-induced electronic effects and possible involvement in hydrogen bonding. This shift serves as clear evidence for the formation of the metal–ligand complex 1. The signals corresponding to the isopropyl group in the ligands of complex 1 were identified, with the methine (–CH) proton appearing as a broad singlet at *δ* 5.93 ppm, and the methyl (–CH_3_) protons resonating as a doublet in the region of *δ* 1.58–1.60 ppm. The aromatic protons of the ligand were observed in the chemical shift range of *δ* 6.85–7.28 ppm. In the ^13^C NMR spectrum, the thio-carbonyl carbon (C=S) of the free perimidine-2-thione ligand appears at *δ* 175.81 ppm. Upon coordination, this signal shifts upfield to *δ* 171.07 ppm, indicating increased electron density and enhanced shielding of the C=S carbon due to the sulfur–metal bonding (Fig. S1†).

### 2.3. Crystal data of complex 1

An ORTEP view of 1 with atom numbering is presented in Fig. 1. The crystallographic data and selected bond lengths and bond angles are given in Tables 1 and 2, respectively. The single-crystal X-ray diffraction studies revealed that the compound in solid-state crystallized in the triclinic system in the *P*1̄ space group, and it is a neutral complex. The mercury(ii) metal exhibits a four-coordinate geometry, with two coordination sites occupied by the sulfur atoms of the ligand molecules, while the remaining two sites are bonded to chlorine atoms. The estimated *τ*_4_ parameter for 1 is 0.92, which validates the slightly distorted tetrahedral coordination geometry around the Hg(ii) center.^73^ The important bond distances Hg1–Cl4; 2.443(3) Å, Hg1–Cl3; 2.448(3) Å, Hg1–S2; 2.540(2) Å, Hg1–S1; 2.596(2) Å and bite angles Cl4–Hg1–Cl3; 112.76(10)°, Cl3–Hg1–S2; 117.10(9)°, Cl4–Hg1–S1; 113.61(9)°, S2–Hg1–S1; 104.16(7)° around the mercury(ii) center are similar to the reported Hg(ii) complex, such as HgCl_2_(1-benzoyl-3-phenylthiourea)_2_.^74^

**Table 1:** Crystal data and structure refinement details of 1

	1. C_2_H_6_OS
Chemical formula	C_28_H_28_Cl_2_HgN_4_S_2_·C_2_H_6_OS
Compound weight (g mol^−1^)	834.28
Temperature (K)	300(2)
Crystal system	Triclinic
Space group	*P*1̄
*a* (Å)	9.2166(7)
*b* (Å)	13.6839(9)
*c* (Å)	13.6960(9)
*α* (°)	74.558(2)
*β* (°)	76.599(2)
*γ* (°)	79.748(2)
Volume (Å^3^)	1607.14(19)
*Z*	2
Density (g cm^−3^)	1.722
Crystal dimensions (mm^3^)	0.109 × 0.186 × 0.206
Crystal color	Yellow
*μ* (mm^−1^)	9.825
*θ* Range (deg)	*θ*_min_ = 1.94, *θ*_max_ = 28.30
Index ranges	−12 ≤ *h* ≤ 12
	−18 ≤ *k* ≤ 18
	−18 ≤ *l* ≤ 18
Unique data	7971
Observed data [*I* > 2*σ*(*I*)]	4532
*F* (000)	822
*R*1	0.0602
w*R*^2^	0.1538
GooF	1.023
No. parameters	379
Transmission factors	*T*_min_ = 0.3450, *T*_max_ = 0.5690
Largest difference map hole (e Å^−3^)	Δ*ρ*_min_ = −0.923 Δ*ρ*_max_ = 1.969
CCDC	2446065

**Table 2:** Selected bond distances (Å) and bond angles (°) for Hg(ii) complex 1

Bond angles (°)		Bond distances (Å)	
Cl4–Hg1–Cl3	112.76(10)	Hg1–Cl4	2.443(3)
Cl3–Hg1–S2	117.10(9)	Hg1–Cl3	2.448(3)
Cl4–Hg1–S2	107.33(10)	Hg1–S2	2.540(2)
Cl4–Hg1–S1	113.61(9)	Hg1–S1	2.596(2)
Cl3–Hg1–S1	101.65(10)	S1–C11	1.716(7)
S2–Hg1–S1	104.16(7)	S2–C14	1.739(7)
C11–S1–Hg1	107.7(3)	N1–C7	1.438(9)
C14–S2–Hg1	105.2(2)	N1–C12	1.484(9)
		N2–C11	1.336(9)
		N2–C1	1.394(9)
		N3–C14	1.326(9)
		N3–C15	1.391(9)
		N4–C14	1.341(9)
		N4–C17	1.424(9)
		N4–C26	1.486(10)
		C1–C2	1.367(11)
		C1–C6	1.399(11)
		C15–C19	1.368(11)
		C16–C22	1.421(10)
		C5–C6	1.428(10)
		C9–C10	1.338(13)

### 2.4. Optimization of solvent, temperature, and catalyst loading

Table 3 presents the findings of the study examining the relationship among catalyst concentration, reaction temperature, and solvent influence, aimed at optimizing the reaction conditions. To optimize the reaction conditions, the Beckmann rearrangement of acetophenone oxime (3a) to *N*-phenylacetamide (4a) was selected as a model reaction under a nitrogen atmosphere. The initial investigation focused on the impact of catalyst loading and reaction temperature, with findings indicating that a 5 mol% of catalyst 1 at 80 °C in acetonitrile produced a high yield (up to 96%) (Table 3, entry 1). Decreasing the catalyst loading from 3 to 1 mol% while maintaining a temperature of 80 °C with acetonitrile as the solvent results in a notable reduction in the product yield (Table 3, entries 2–3). The lack of catalysts in the reaction, conducted under the same conditions as previously, is notable; however, there was no observed progress in the conversion of amides (Table 3, entry 4). Despite an extended duration for the reaction at room temperature, no observable reaction occurs, and the yield diminishes as the reaction temperature decreases from 80 °C to 50 °C (Table 3, entries 5–6). The solvents were integral to the Beckmann rearrangement of acetophenone oxime, facilitated by Hg(ii) complex 1 in a nitrogen atmosphere. Several polar protic (water), polar aprotic (1,4-dioxane, tetrahydrofuran, acetone, dimethyl sulfoxide, and dimethylformamide), and non-polar (toluene) solvents were tested, and the results are summarized in Table 3. Solvents, such as 1,4-dioxane, acetone, and THF, result in a diminished conversion of amides, while toluene, DMSO, and DMF produce only negligible amounts of the product (Table 3, entries 7–12). Using water as the solvent for the Beckmann rearrangement within 24 hours did not result in any visible products, possibly due to the catalyst being insoluble in water (Table 3, entry 13). It demonstrates the significance of the solvent in this reaction. Consequently, acetonitrile emerged as the optimal solvent for the Beckmann rearrangement reaction in the current investigation.

**Table 3:** Optimization of catalyst loading, solvent, and temperature for the Beckmann rearrangement reaction of benzophenone oxime^a^

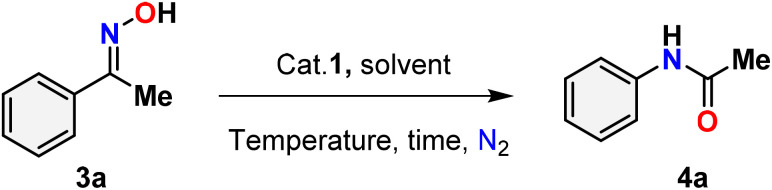					
Entry	Catalyst (mol%)	Solvent	*T* (°C)	Time (h)	Yield^b^ %
1	HgCl_2_(N^i-Pr^PmT)_2_ (5)	CH_3_CN	80	12	96
2	HgCl_2_(N^i-Pr^PmT)_2_ (3)	CH_3_CN	80	24	78
3	HgCl_2_(N^i-Pr^PmT)_2_ (1)	CH_3_CN	80	24	56
4	—	CH_3_CN	80	24	No reaction
5	HgCl_2_(N^i-Pr^PmT)_2_ (5)	CH_3_CN	50	24	68
6	HgCl_2_(N^i-Pr^PmT)_2_ (5)	CH_3_CN	25	24	No reaction
7	HgCl_2_(N^i-Pr^PmT)_2_ (5)	1,4-Dioxane	80	12	11
8	HgCl_2_(N^i-Pr^PmT)_2_ (5)	THF	80	12	26
9	HgCl_2_(N^i-Pr^PmT)_2_ (5)	Acetone	80	12	18
10	HgCl_2_(N^i-Pr^PmT)_2_ (5)	Toluene	80	12	Trace
11	HgCl_2_(N^i-Pr^PmT)_2_ (5)	DMSO	80	12	>5
12	HgCl_2_(N^i-Pr^PmT)_2_ (5)	DMF	80	12	Trace
13	HgCl_2_(N^i-Pr^PmT)_2_ (5)	Water	80	24	No reaction

a Reaction conditions: Oxime (1 mmol), specified quantity of catalyst HgCl_2_(N^i-Pr^PmT)_2_ (1), solvent (3 mL), temperature, time.

b Isolated yields were reported after column chromatography.

### 2.5. Catalytic properties

As shown in Table 4, the effectiveness and compatibility of the acetonitrile solution of mercury compound 1-catalyzed Beckmann rearrangement of oximes were thoroughly investigated under ideal reaction conditions. In order to assess the degree to which the Hg(ii) complex 1 facilitates the Beckmann rearrangement, a diverse array of oxime compounds (3a–3ag) were initially synthesized from their respective ketone substrates (2a–2ag), incorporating various structural and electronic modifications.^75,76^ A variety of functional groups, such as methyl, ethyl, methoxy, hydroxy, trifluoromethyl, halo (–F, –Cl, –Br), and amino-substituted acetophenone oximes, propiophenone oxime, butyrophenone oximes, benzophenone oximes, and cyclohexanone oxime, were well tolerated, and treated under standard reaction conditions for rearrangement to afford the corresponding amides 4a–4y in high to excellent yields (71–97%). All acetophenone oximes were successfully converted into *N*-phenylacetamides (4a–4o) under specified conditions with good yields ranging from 71% to 96% (Table 4, entries 1–15). The substrates listed here follow the customary migratory aptitude of Beckmann rearrangement.

**Table 4:** Beckmann rearrangement of ketoximes to amides catalyzed by Hg(ii) complex 1^a^

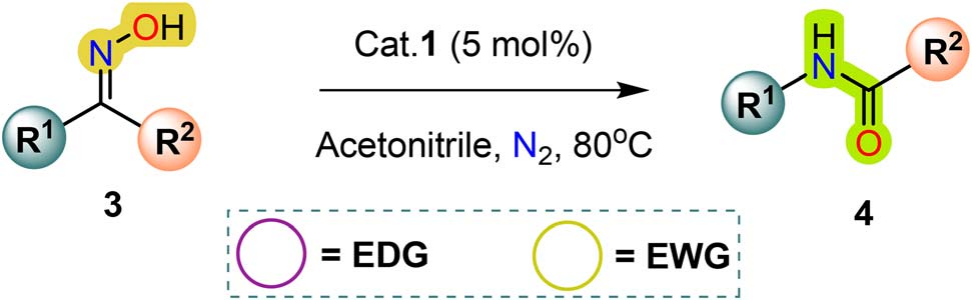			
Entry	Ketoximes	Amides	Yield (%)
1	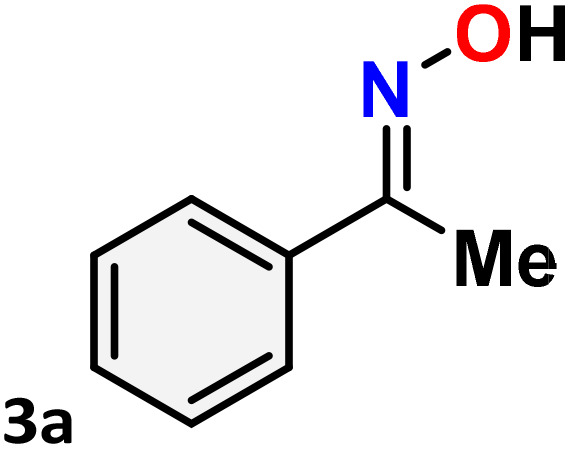	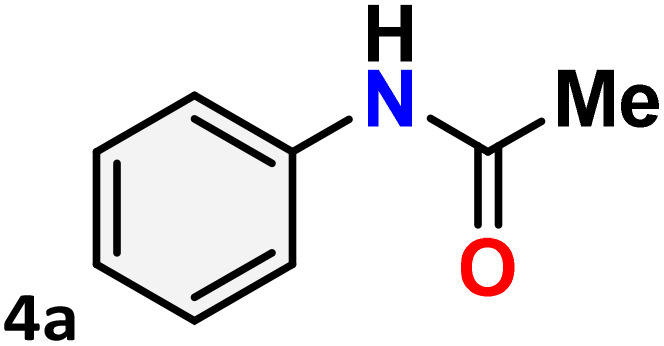	95
2	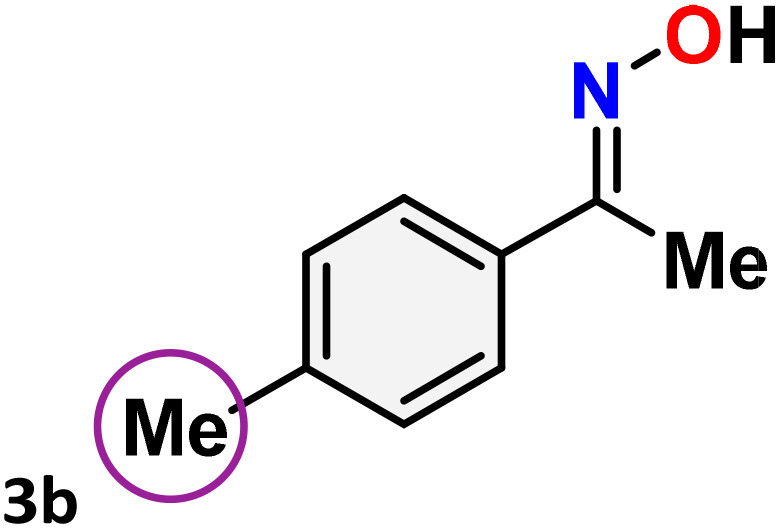	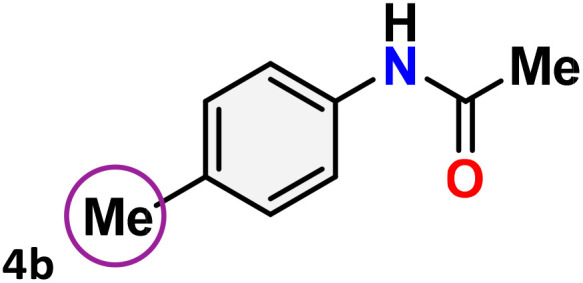	91
3	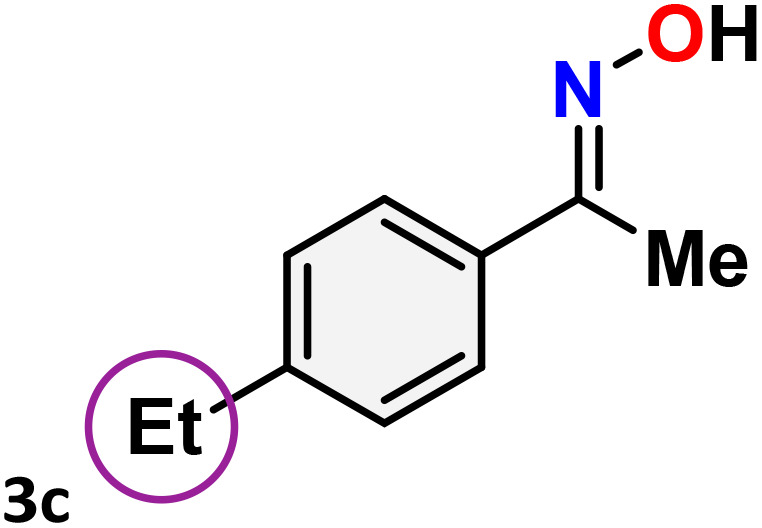	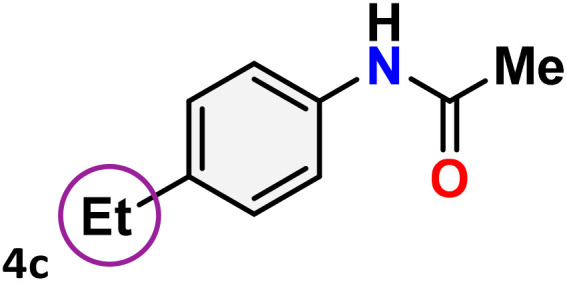	89
4	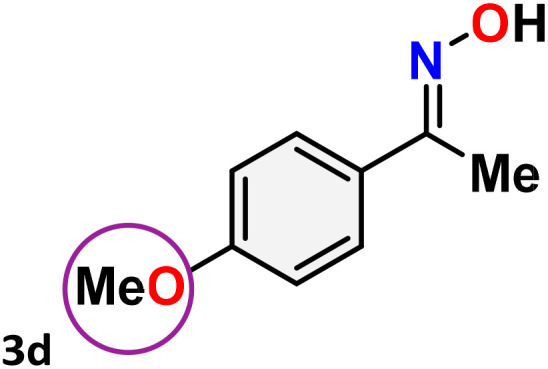	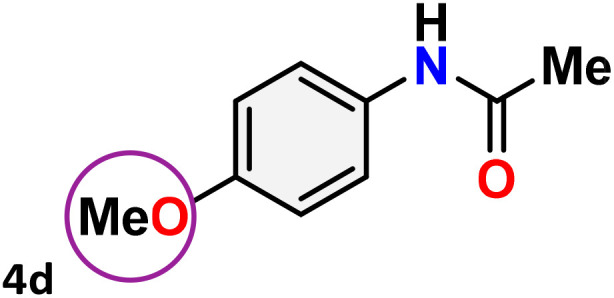	93
5	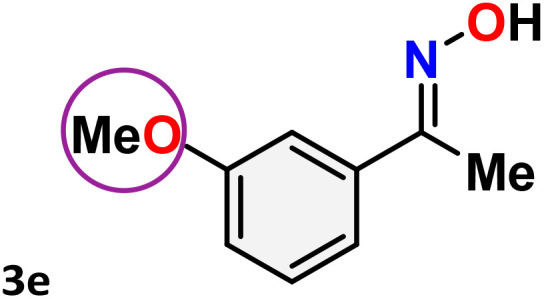	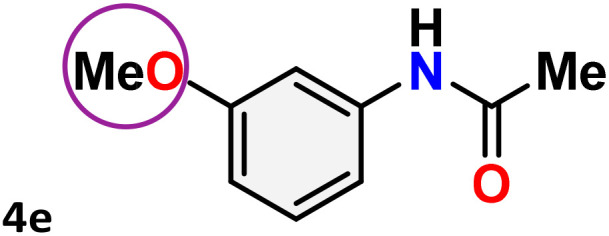	85
6	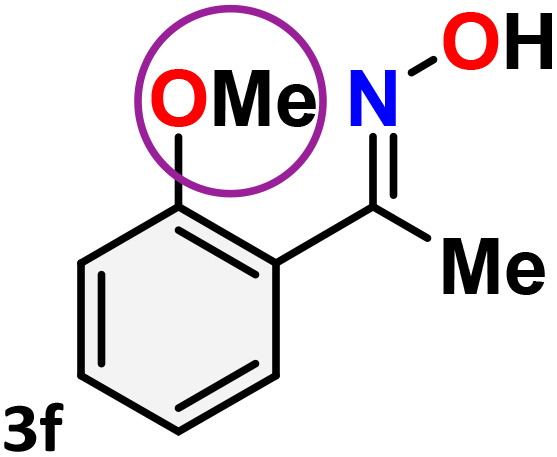	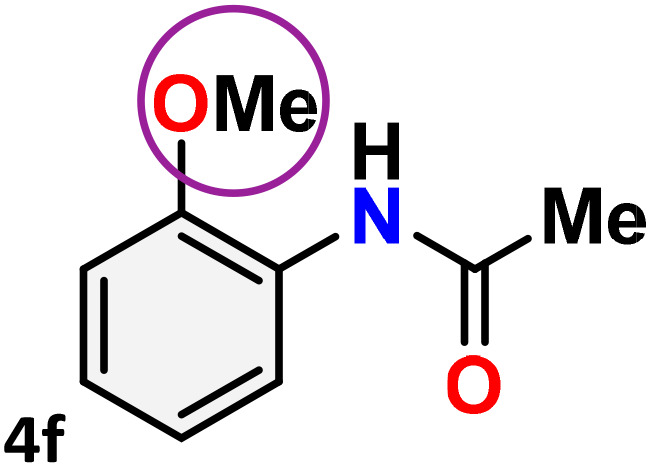	81^b^
7	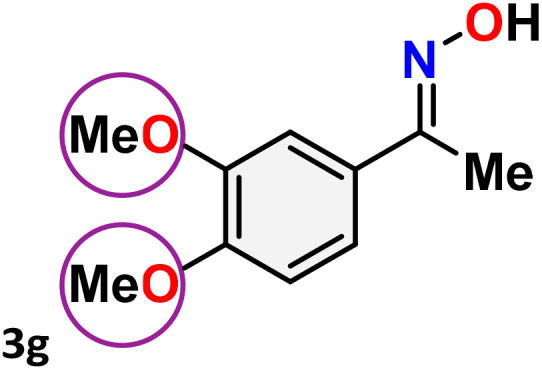	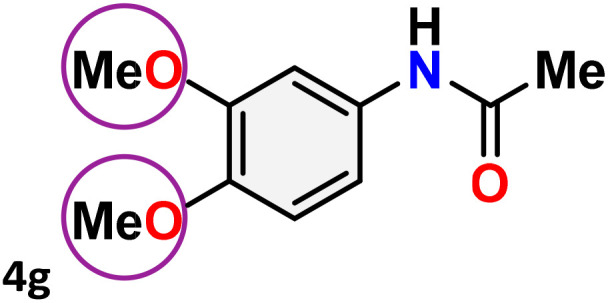	96
8	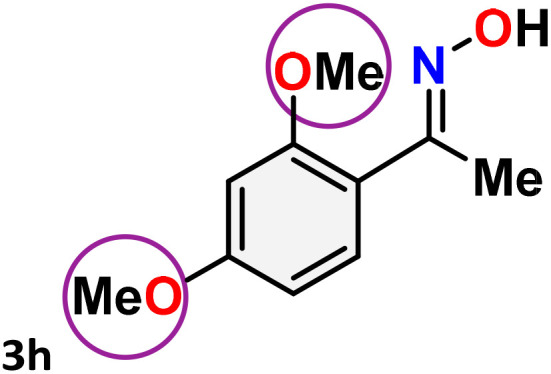	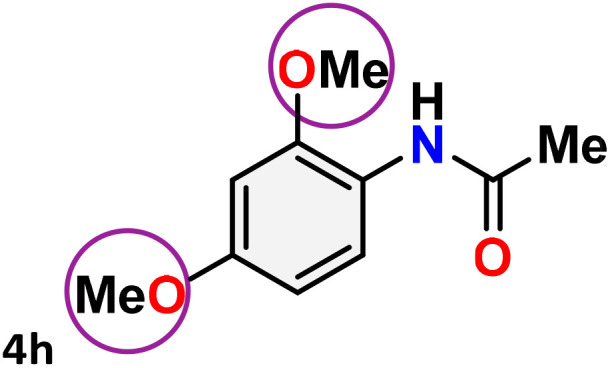	91
9	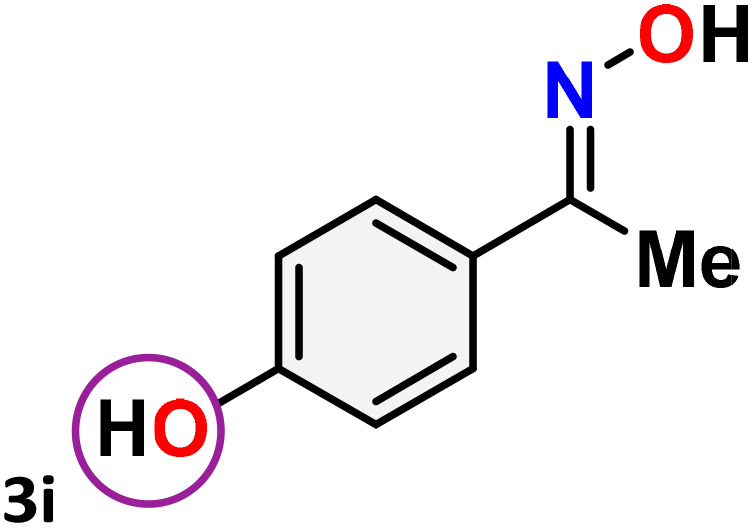	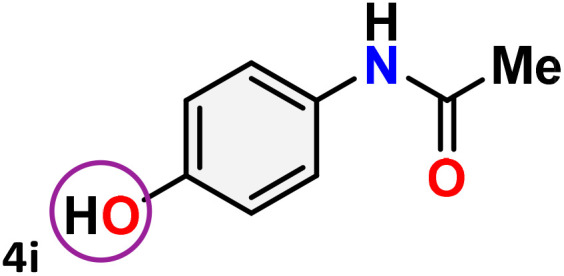	90
10	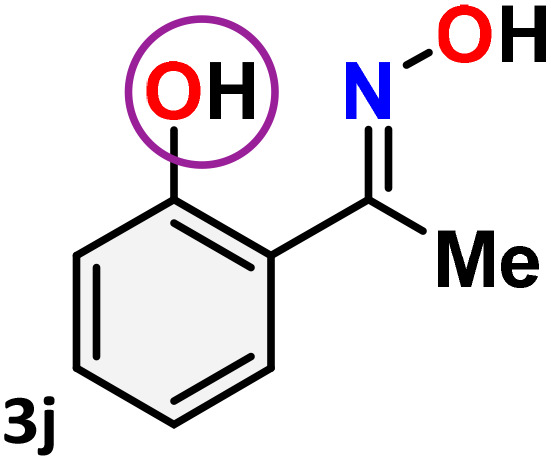	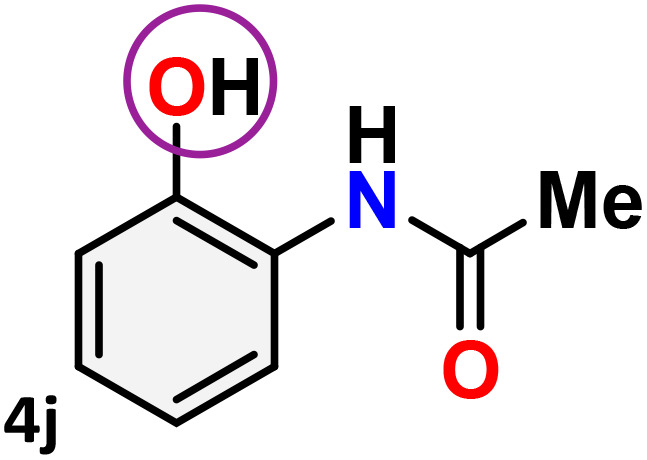	78^b^
11	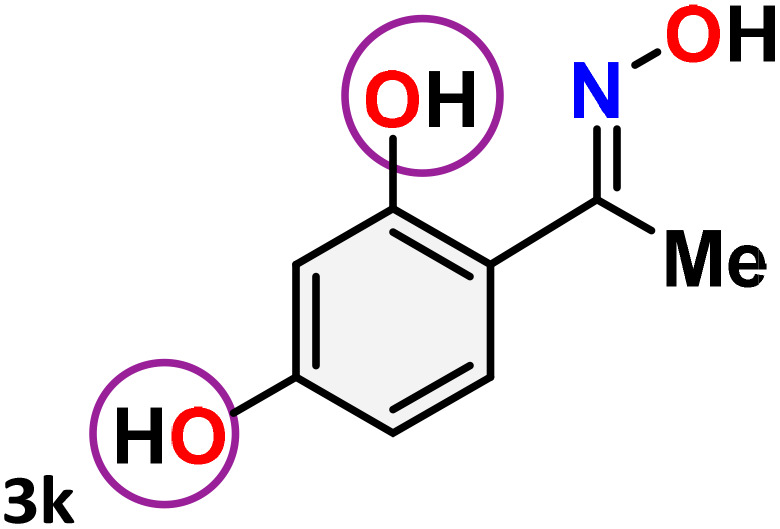	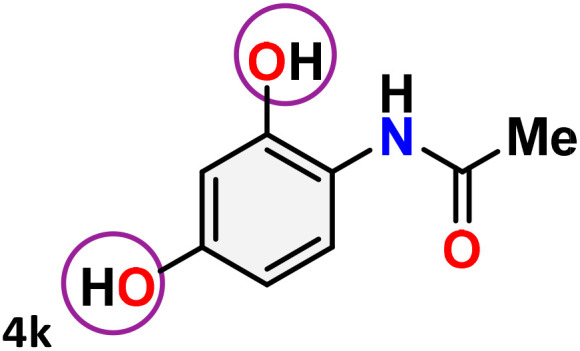	71
12	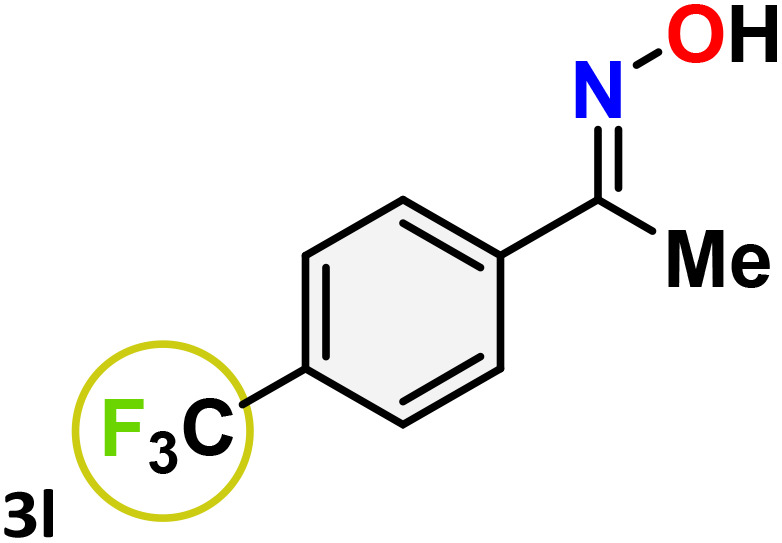	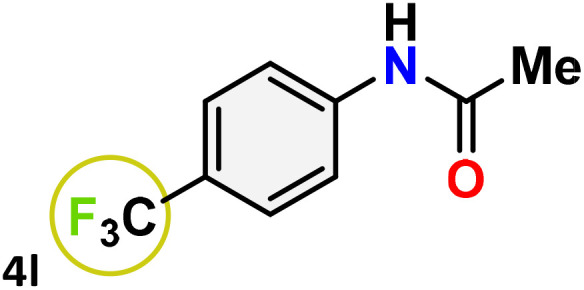	92
13	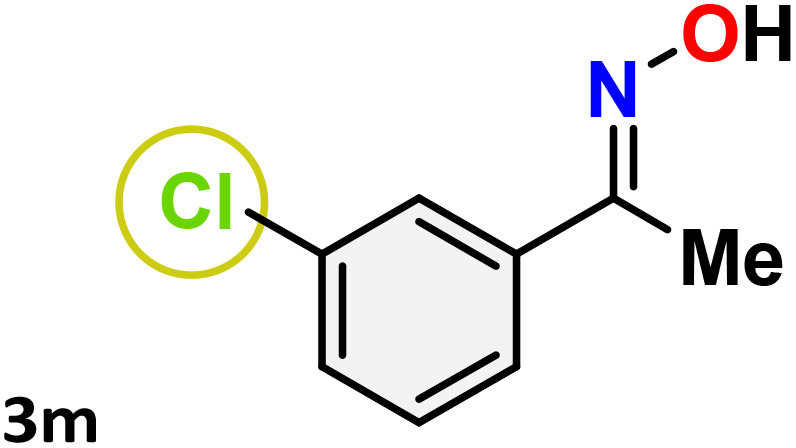	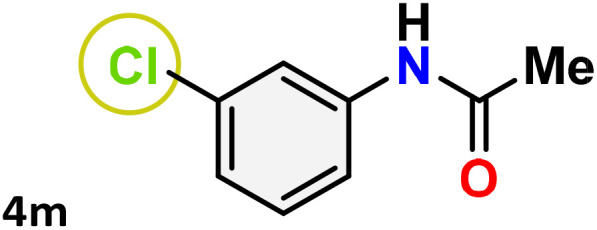	91
14	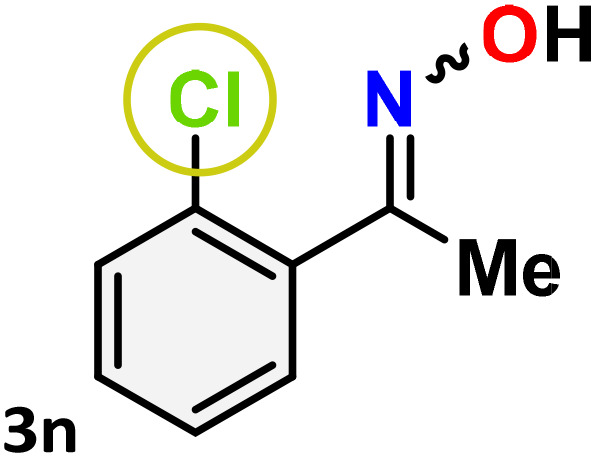	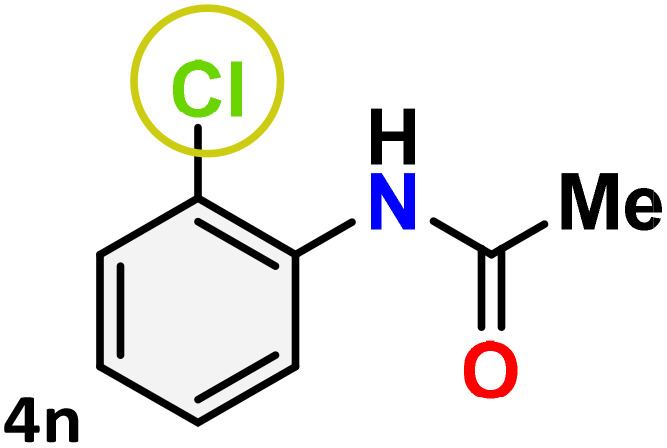	89^b^^,^^c^
15	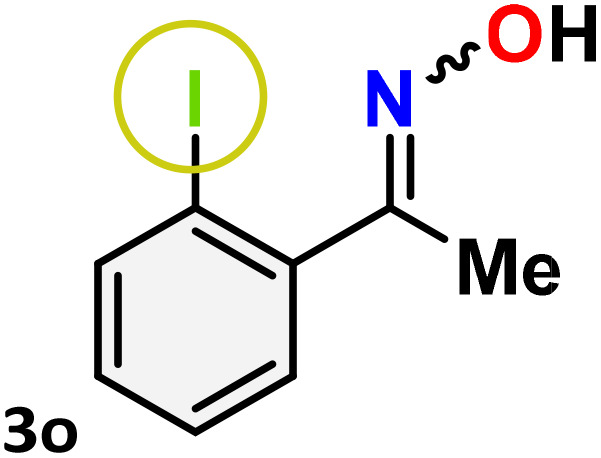	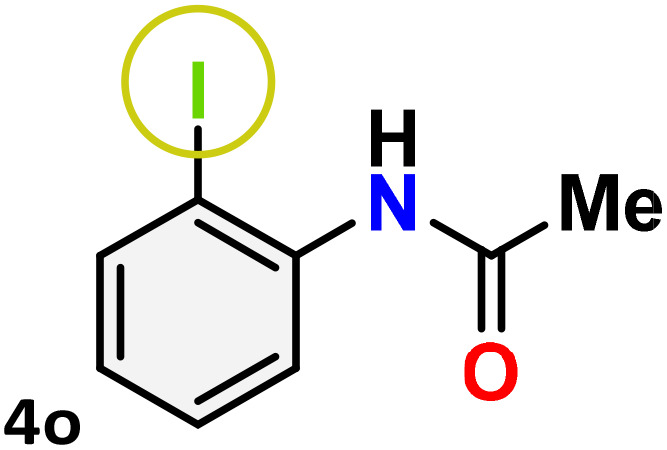	79^b^^,^^c^
16	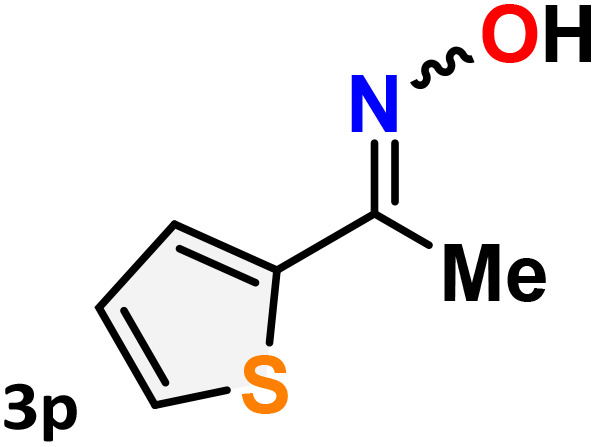	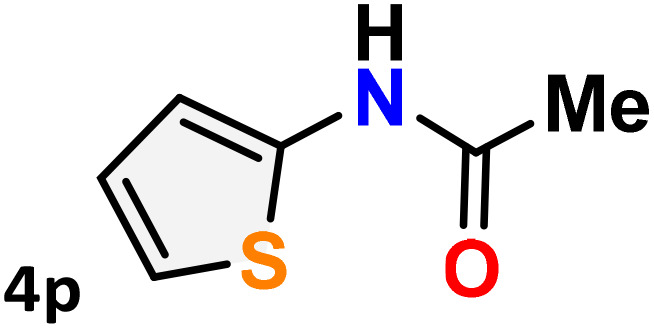	86^c^
17	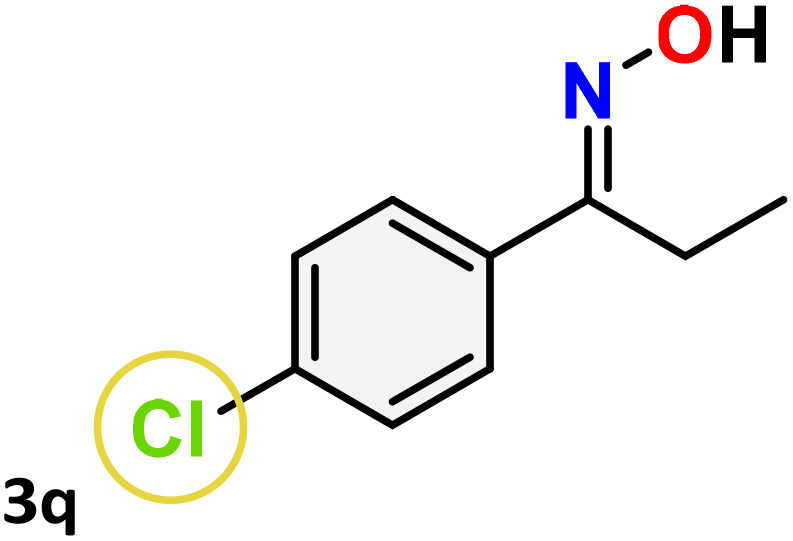	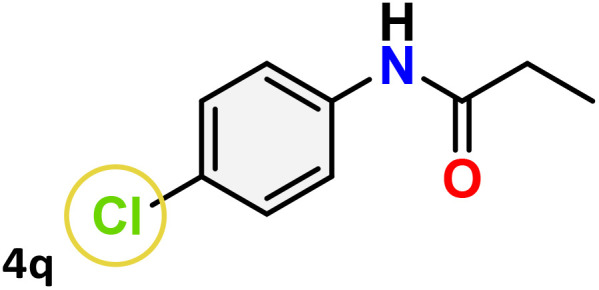	87
18	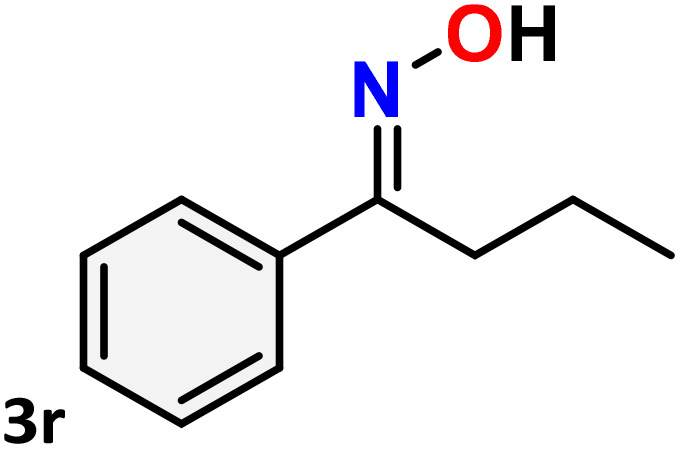	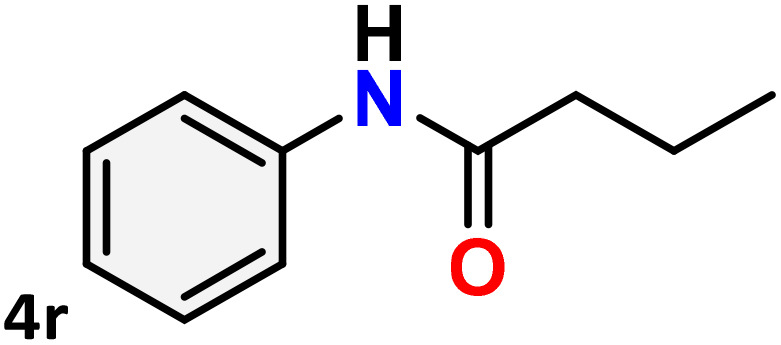	72
19	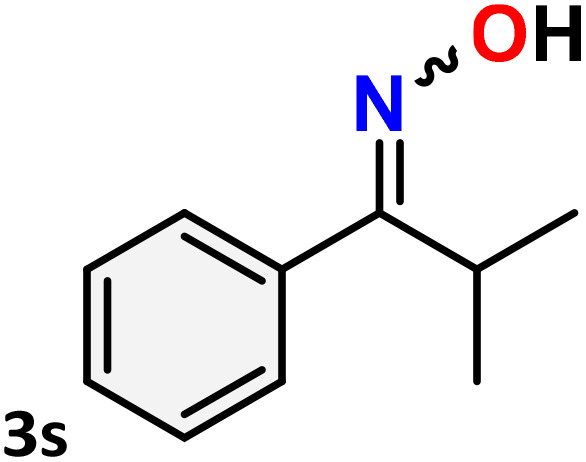	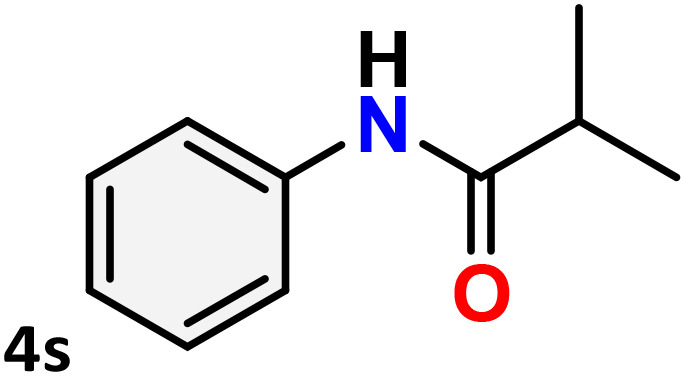	83^c^^,^^d^
20	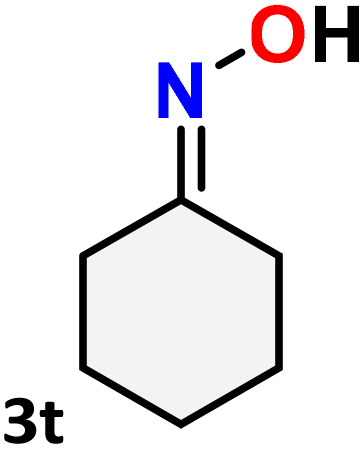	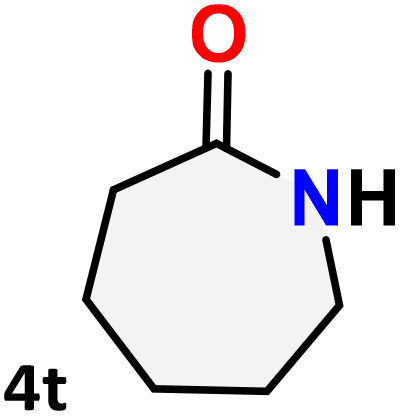	92^e^
21	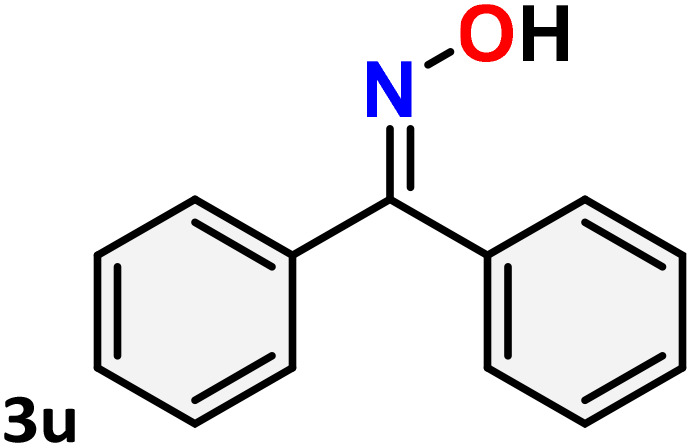	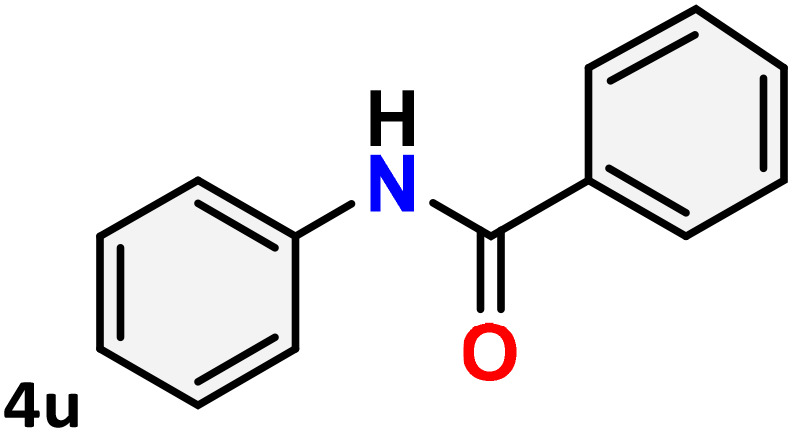	97
22	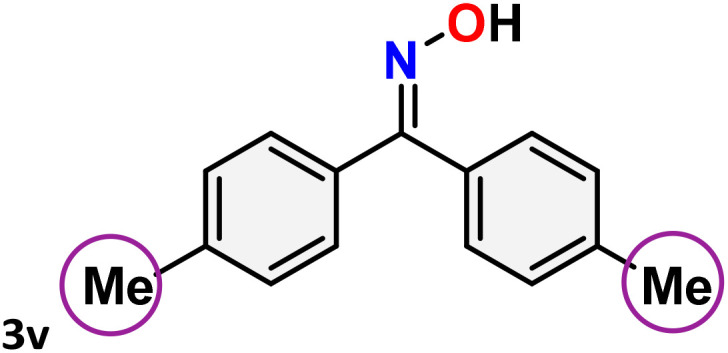	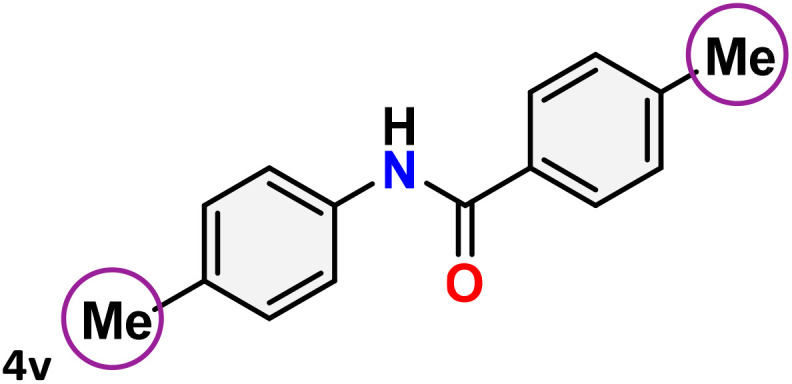	94
23	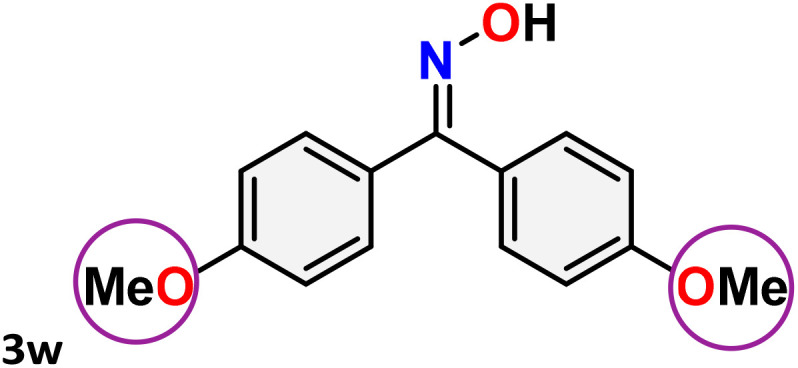	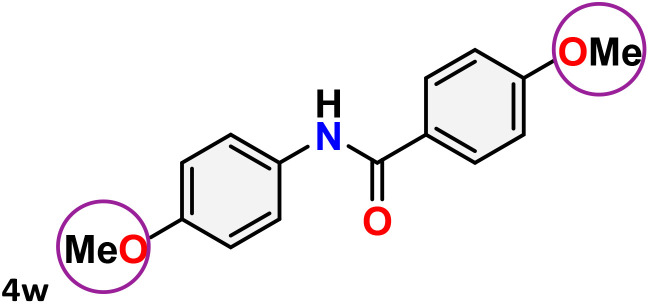	90
24	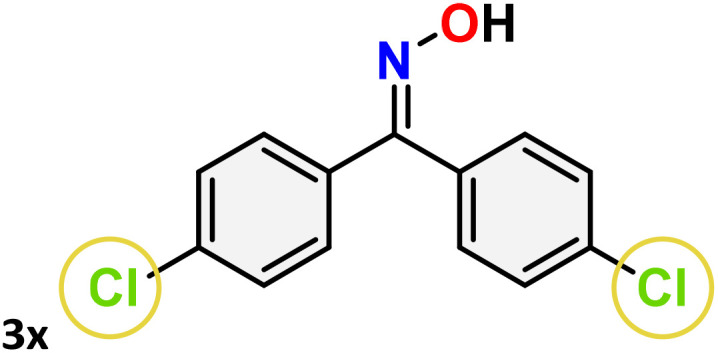	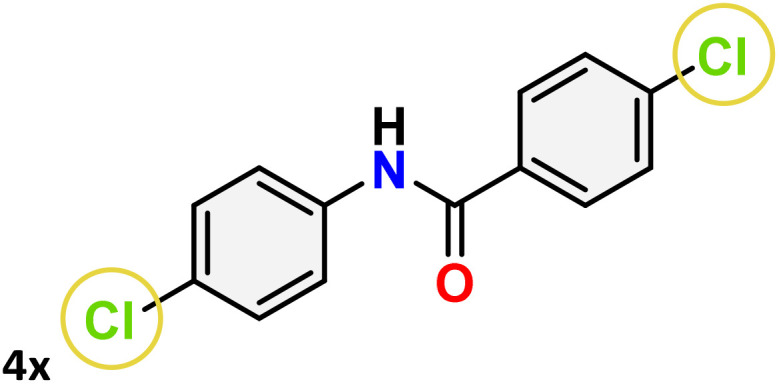	89
25	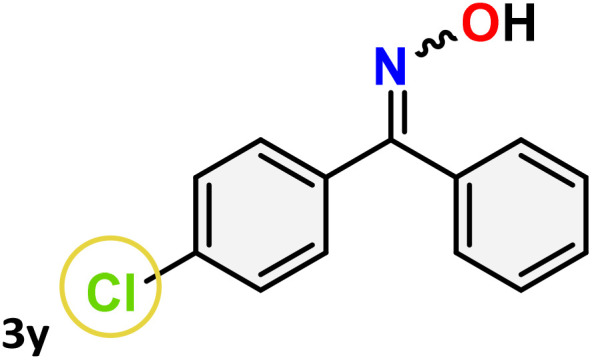	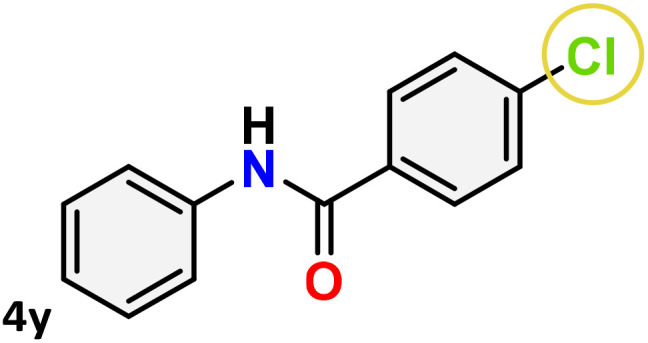	86^c^^,^^d^
26	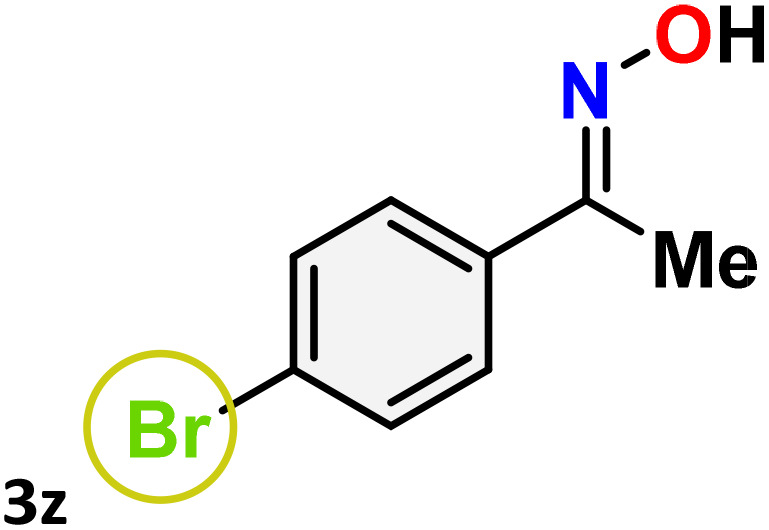	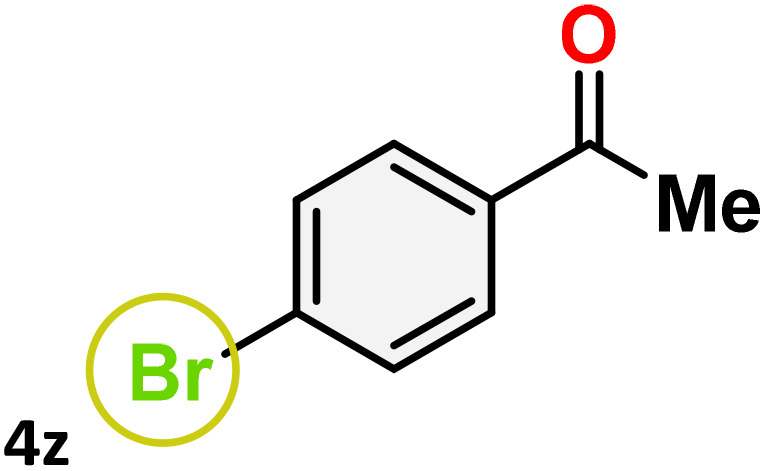	99
27	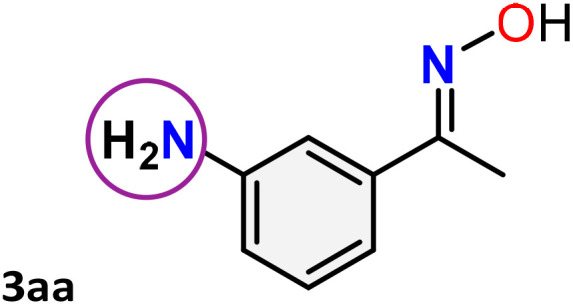	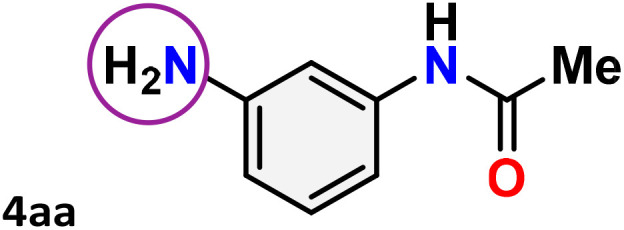	N.D^f^
28	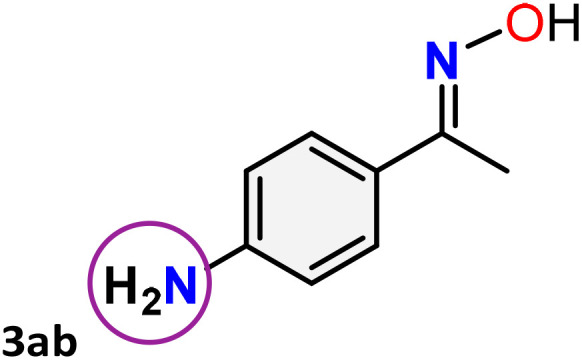	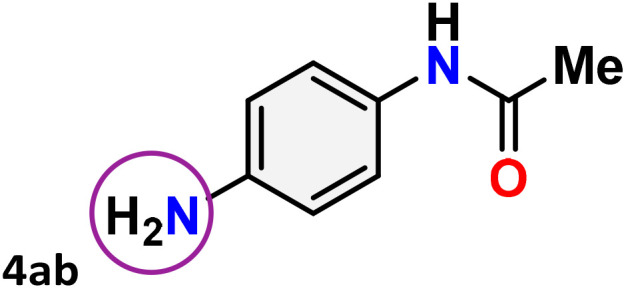	N.D^f^
29	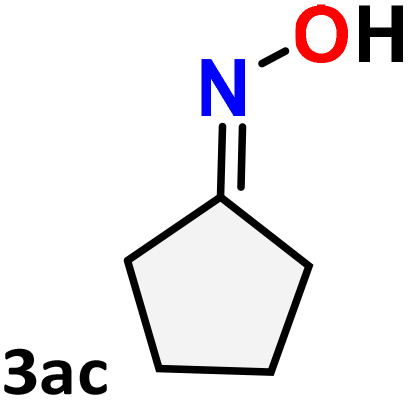	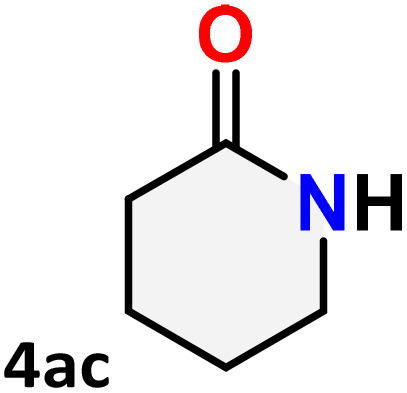	N.D^f^
30	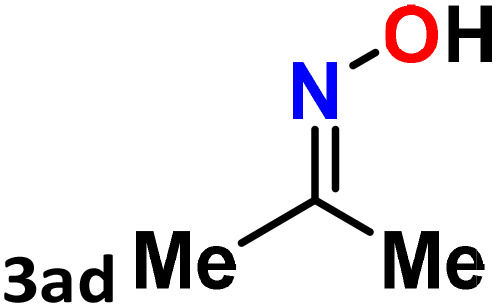	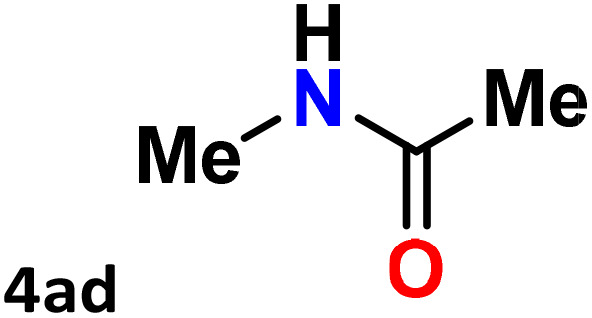	N.D^f^
31	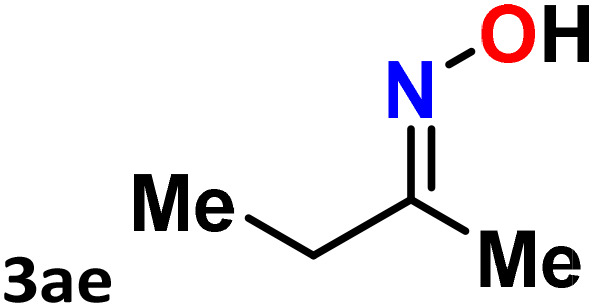	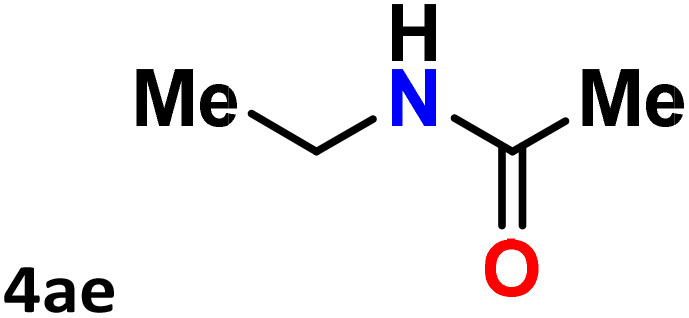	N.D^f^
32	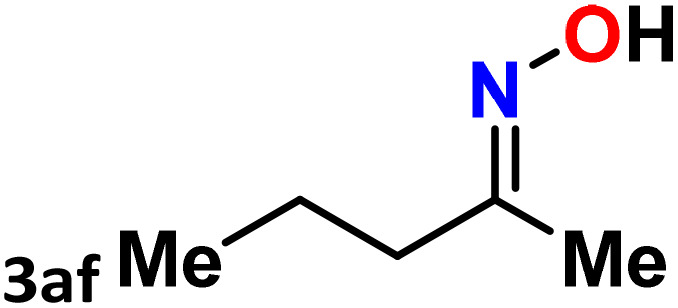	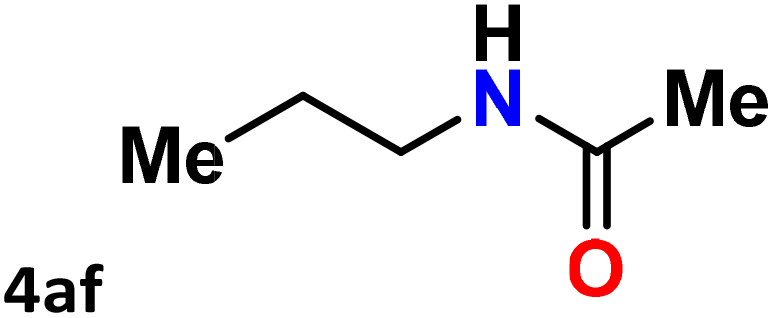	N.D^f^
33	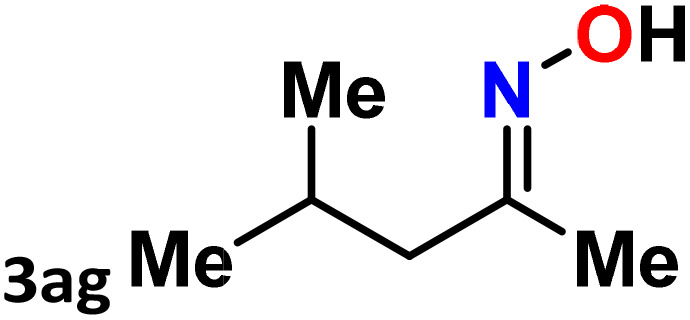	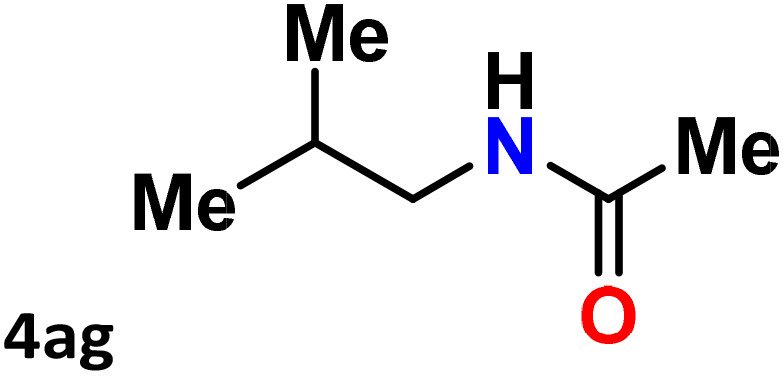	N.D^f^

a Reaction conditions: oxime (1 mmol), catalyst 1 (5 mol%, 0.05 mmol), CH_3_CN (3 mL), 80 °C, N_2_, 12 h. Isolated yields were reported after column chromatography.

b 18 h.

c Oximes were prepared as a mixture of isomers.

d Overall yield of the isomeric mixtures 
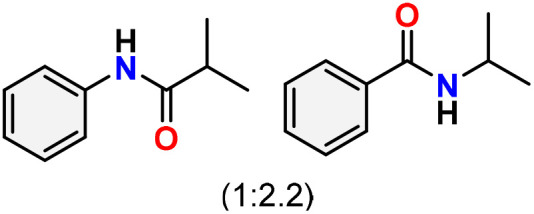

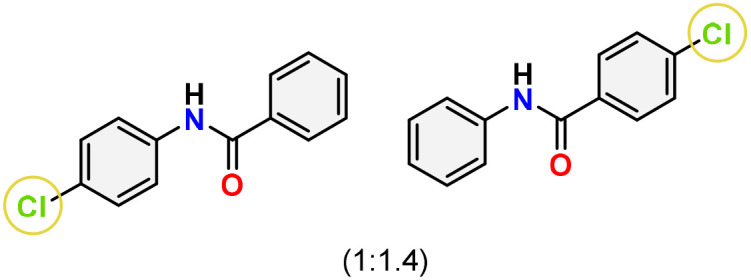
.

e Used 8 mol% of catalyst 1, 24 h.

f Not detected.

For instance, in all cases of acetophenone and substituted acetophenone oximes, the migration of the phenyl group is the only one visible, with no discernible products resulting from the migration of the alkyl group. When juxtaposed with -*p* and -*m* substituted acetophenones, the -*o* substituted acetophenone oxime necessitates a prolonged reaction duration and culminates in a relatively diminished yield (Table 4, entries 6, 10, 11, 14–15). Notably, oximes that included a heteroatom (3p, obtained as a mixture of *E*/*Z* isomers) also showed remarkable reactivity, producing the *N*-(thiophen-2-yl)acetamide product with great efficiency (Table 4, entry 16). Along with acetophenone oximes, various alkyl phenyl ketoximes, including 4′-chloropropiophenone oxime (3q), *n*-butyrophenone oxime (3r), and isobutyrophenone oxime (3s), demonstrated good tolerance, resulting in the respective products 4q–4s with yields of 87%, 72%, and 83%, respectively (Table 4, entries 17–19). The oxime of cyclic ketones, similar to the challenging cyclohexanone oxime, is very reactive and forms ε-caprolactam (4t), an important building block for the Nylon-6 polymer (Table 4, entry 20). The catalyst also reacted well with benzophenone and its substituted oxime derivatives (3u–3x), producing the corresponding products in excellent yields (4u–4x). The symmetrical bis(aryl)methanone oxime substrates were subsequently analyzed under the established conditions, yielding *N*-(aryl) benzamide products in high yields (Table 4, entries 21–24). Interestingly, the unsymmetrical oxime (4-chlorophenyl)(phenyl)methanone oxime (3y) underwent preferential migration of the electron-rich phenyl group, yielding 4-chloro-*N*-phenylbenzamide (4y) as the major isomer. Furthermore, a small quantity of the isomer arising from the migration of the electron-deficient 4-chlorophenyl group was also observed (Table 4, entry 25). On the other hand, only the hydrolysis product, acetophenone, was observed for the *p*–Br substituted acetophenone oxime (3z) as shown in Table 4, entry 26. Inevitably, oxime substrates featuring nucleophilic functional groups, such as –NH_2_ (3aa–ab), exhibited no evidence of reactivity in Beckmann rearrangement (Table 4, entries 27–28). Additionally, certain aliphatic oximes (3ac–3ag) exhibited incompatibility with this protocol (Table 4, entries 29–33). Beckmann rearrangement of *O*-methyl oximes is generally not favorable due to the absence of a free hydroxyl group, which is critical for activation and rearrangement. In this study, the treatment of (*E*)-1-phenylethan-1-one *O*-methyl oxime (3ah) with Hg(ii) catalyst 1 under standard reaction conditions failed to form the corresponding amide product (Scheme S1, page S78†). This is consistent with literature reports and underscores the importance of the free oxime OH group in promoting the rearrangement. These findings suggest that the electronic effects significantly influence the catalytic efficiency.

**Scheme 4:**
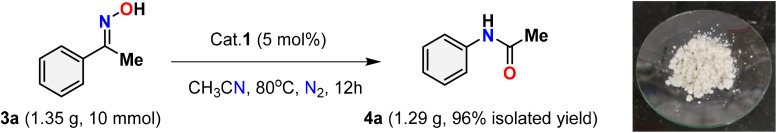
Gram-scale synthesis of *N*-phenylacetamide 4a.

**Scheme 5:**
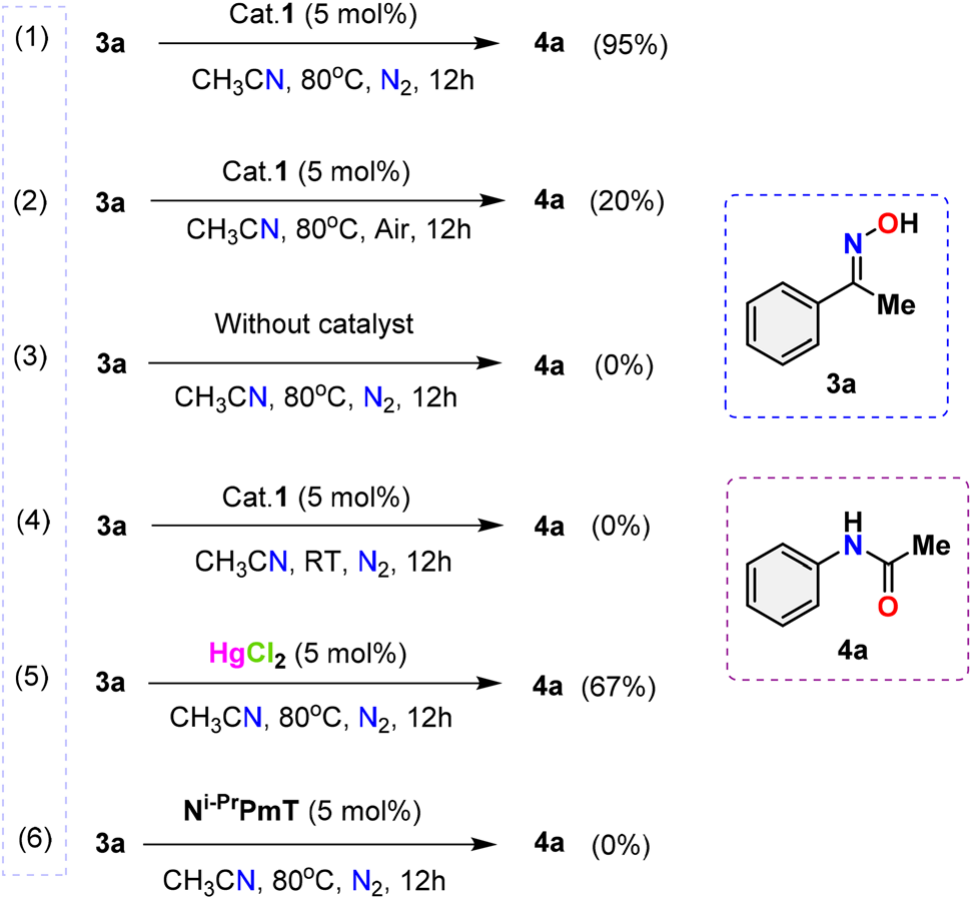
Controlled experiments for investigating the mechanism.

One purpose of the gram-scale synthesis of *N*-phenyl acetamide 4a was to investigate the synthetic efficacy of the HgCl_2_(N^i-Pr^PmT)_2_ (1) assisted Beckmann rearrangement in the presence of acetonitrile (Scheme 4). Under standard conditions, the reaction of acetophenone oxime 3a proceeded smoothly, delivering the desired product 4a in 96% isolated yield. A sequence of meticulously executed experiments was conducted to explore the potential reaction mechanism (Scheme 5). Interestingly, the reaction proceeded even in the absence of an inert nitrogen atmosphere, albeit with significantly reduced yield (2). Further experimental data indicated that the presence of the Hg(ii) catalyst (3) and elevated temperature conditions (4) were indispensable for the reaction to occur. Significantly, the use of mercury salt alone under the same reaction conditions led to a reduced yield, whereas the perimidine-2-thione ligand (N^i-Pr^PmT) by itself failed to promote any reaction progress (5) and (6). These observations underscore the indispensable role of the combined catalytic system in driving the transformation. Collectively, the results confirm that the presence of catalyst 1, an inert N_2_ atmosphere, and thermal activation are all vital factors for the effective execution of Beckmann rearrangement.

### 2.6. Proposed mechanism

**Scheme 6:**
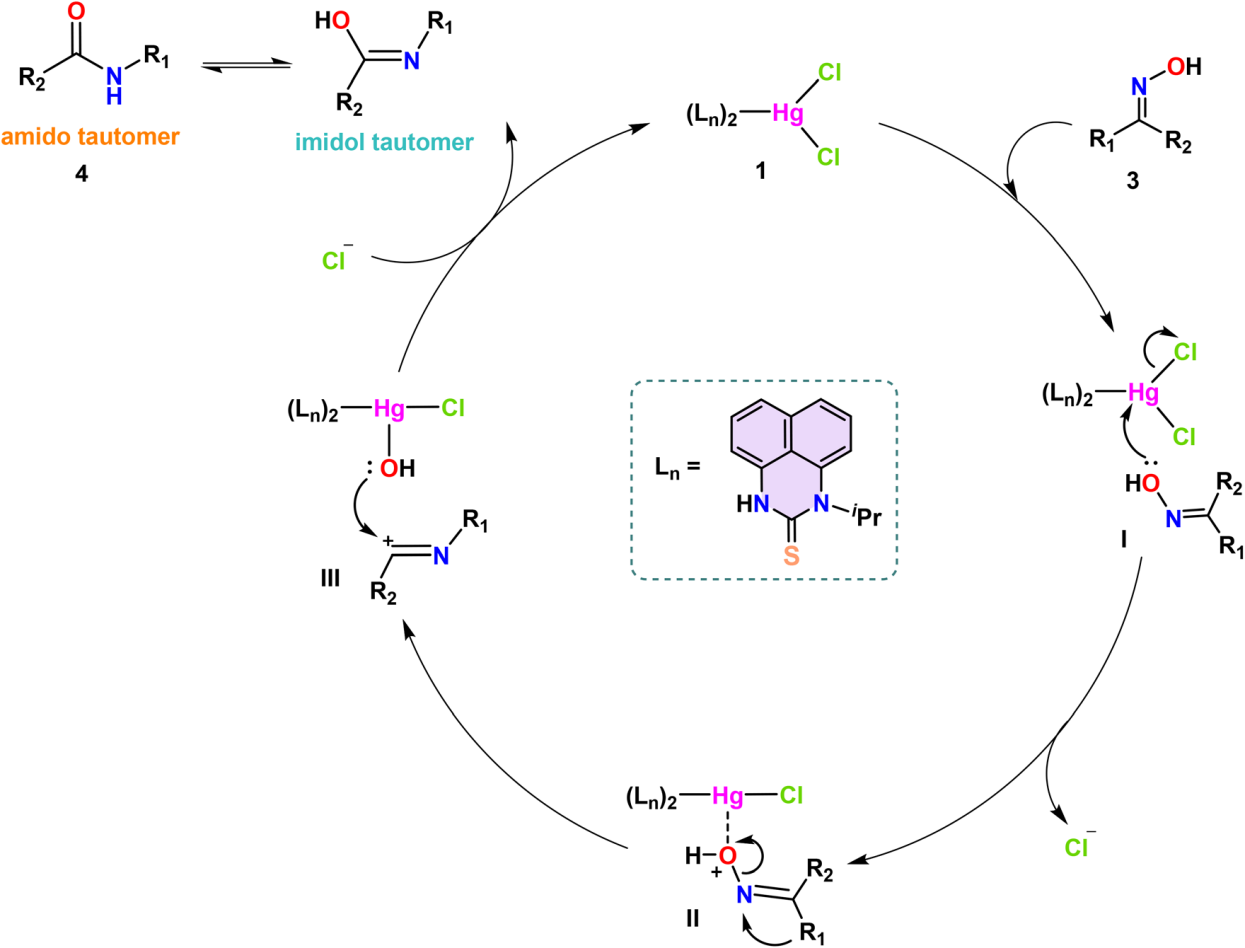
Proposed mechanism for the Hg(ii) complex 1-catalyzed Beckmann rearrangement of ketoximes to amides.

The plausible mechanism for the complex 1-catalyzed Beckmann Rearrangement of ketoximes is shown in Scheme 6. Beckmann rearrangement is recognized for its stereospecific nature; notably, the group on the ketoxime that is positioned anti to the hydroxyl group tends to migrate selectively. The catalytic cycle commences with the engagement of the Hg(ii) catalyst 1 and the oxime molecule (3), resulting in the formation of the intermediate I. An adduct-like oxonium–mercury complex II was produced when catalyst 1 had one chlorine atom cleaved due to the attack of a single pair of electrons from the hydroxyl group attached to the imine nitrogen on the electrophilic mercury center. Given the inherent instability of the oxonium cation, the alkyl or aryl group positioned anti-periplanar to the oxime oxygen transitions to the nitrogen atom, resulting in the formation of complex III. Additionally, the hydroxyl group from the oxo–mercury complex attacks the cationic imine moiety with its lone pair, producing the imidol form, which undergoes tautomerism and yields the final desired amide 4.

## 3 Conclusion

In summary, the objectives of the current work were successfully achieved, including the synthesis and structural characterization of the four-coordinated mercury(ii) complex 1 with a perimidine-2-thione ligand of the general formula HgCl_2_(N^i-Pr^PmT)_2_. A single crystal X-ray diffraction study revealed the absolute structural parameter, which confirmed that complex 1 crystallizes in a triclinic system with a *P*1̄ space group. Two perimidin-2-thione ligands and two chlorine atoms as an ancillary ligand comprise the fundamental structure of the mercury(ii) complex, which is a neutral, four-coordinated, distorted tetrahedral structure. Thus far, the Beckmann rearrangement reaction has only been catalyzed by an extremely limited amount of Lewis acidic metal complexes.

Therefore, we attempted to employ this Hg(ii) catalyst for the conversion of oximes to amides, which is formally known as the Beckmann rearrangement. It is interesting to note that the produced mercury complex 1 functioned as a powerful catalyst for the well-known Beckmann rearrangement, achieving good conversions with just 5 mol% loading, which converts ketoximes into their corresponding amide derivatives. A notable observation was that, when catalyst 1 was present, all of the substrates, both cyclic and acyclic ketoximes that were tested, exhibited outstanding amide conversions. Further research should be undertaken in the future to determine whether other transition metal-based perimidine thione complexes also demonstrate this characteristic Beckmann rearrangement reaction, or if this reactivity is uniquely associated with mercury complexes.

## 4 Experimental section

### 4.1. Materials required

1-Isoropyl-1*H*-perimidine-2(3*H*)-thione was synthesized according to the previously published procedures.^71^ All the reactions were carried out under a nitrogen atmosphere unless otherwise noted. The chemicals, 1,8-diamino naphthalene, LiAlH_4_, carbon disulfide, potassium hydroxide, HgCl_2_, ketones, sodium acetate, hydroxylamine hydrochloride, chloroform-d (CDCl_3_), dimethyl sulfoxide-d_6_ (DMSO-d_6_), and other solvents, such as acetone, diethyl ether, ethyl acetate, petroleum ether, and ethanol, were purchased from Sigma Aldrich, Avra, SRL and TCI chemicals. All the oximes used were synthesized according to the published procedure from their corresponding ketones. No additional purification was performed on any of the compounds that were purchased commercially. Column chromatography was performed on silica gel (60–120 mesh) purchased from SRL, and thin-layer chromatography (TLC) analyses were performed on commercial silica gel plates (60 F254).

Caution! Mercury and its compounds are hazardous.^77^ Only a minimal amount of these materials should be prepared and handled carefully.

### 4.2. General instrumentation methods

FT-IR spectra were obtained using a Thermo Scientific, Nicolet iS50 instrument equipped with a high-sensitivity DLaTGS detector, and the spectra were recorded using the attenuated total reflectance (ATR) method. The scans were taken in the 400 to 4000 cm^−1^ range. The ^1^H and ^13^C NMR spectra were recorded on a BRUKER (400 MHz) spectrometer. Chemical shifts are reported in parts per million (ppm). Tetramethylsilane (TMS) is used as an internal standard. Single-crystal X-ray diffraction (SCXRD) analysis was performed using a Photon II area detector attached to a three-circle diffractometer. An appropriate crystal was mounted on a nylon loop, and data collection was carried out using Mo Kα radiation (*λ* = 0.71073 Å) with the aid of BRUKER APEX4 software. The crystal structures were solved in the monoclinic symmetry using APEX4 software. The atoms were refined anisotropically. Figures were drawn utilizing DIAMOND-3 software with a 50% probability of thermal ellipsoids.

### 4.3. Synthesis of complex 1

A solution of 1-isopropyl-1*H*-perimidin-2(3*H*)-thione, N^i-Pr^PmT (0.030 g, 0.12 mmol), in THF (10 mL) was added to an ethanolic solution of mercuric chloride (0.016 g, 0.061 mmol), which afforded a dark green precipitate. The above mixture was stirred for 12 h at room temperature under a nitrogen atmosphere. The green solid was filtered off, washed with distilled water, and vacuum-dried. The residue was added to 1 mL of toluene and kept undisturbed for crystallization. Yellow crystals were obtained after 14 days at −20 °C. Yield: 0.033 g, 75%. Anal. Calcd for C_28_H_28_Cl_2_HgN_4_S_2_: C, 44.48; H, 3.73; N, 7.41; S, 8.48%. Found: C, 43.32; H, 3.671; N, 7.20; S, 8.46%. M.p. = 265–267 °C. FT-IR (Diamond-ATR, neat, cm^−1^): 3150 *ν*(N–H); 3010 *ν*(Aromatic C–H); 2989 *ν*(Aliphatic C–H); 1659 *ν*(C–N); 1162 *ν*(C=S). ^1^H NMR (400 MHz, CDCl_3_) *δ* 11.50 (s, 2H), 7.28–7.21 (m, 8H), 7.04 7.01 (m, 2H), 6.87 6.85 (m, 2H), 5.93 (s, 2H), 1.59 (d, *J* = 7.1 Hz, 12H). ^13^C NMR (100 MHz, CDCl_3_) *δ* 171.1, 134.6, 132.1, 127.1, 122.4, 121.7, 119.9, 109.4, 107.8, 54.8, 18.5.

### 4.4. General procedure for the synthesis of amides and lactams using Beckmann rearrangement

To an oven-dried round bottom flask filled with a solution of the corresponding oximes (1 mmol in 5 mL of acetonitrile) equipped with a reflux condenser and magnetic stirring bar in a nitrogen atmosphere, the mercury(ii) complex 1 (0.05 mmol, 5 mol%) was added. After being stirred at 80 °C for 12 h, the reaction mixture was allowed to cool to room temperature. The completion of the reaction was identified by monitoring TLC, and then 5 mL of acetonitrile was added to dissolve the solid formed. The solvent was removed using a rotary evaporator, and the organic material was dissolved in dichloromethane (3 × 15 mL). The combined organic layer was washed with a brine solution, dried over Na_2_SO_4_, and concentrated to dryness. The residue was purified using flash column chromatography over silica gel (60–120 mesh) with an ethyl acetate-petroleum ether eluent system to afford the respective amides or lactams.

### 4.5. Gram scale synthesis

To an oven-dried round bottom flask filled with a solution of (*E*)-1-phenylethan-1-one oxime 3a (1.35 g, 10 mmol in 15 mL of acetonitrile) equipped with a reflux condenser and magnetic stirring bar in nitrogen atmosphere, mercury(ii) complex 1 (0.05 mmol, 5 mol%) was added. After being stirred at 80 °C for 12 h, the reaction mixture was allowed to cool to room temperature. The completion of the reaction was identified by monitoring TLC, and then 15 mL of acetonitrile was added to dissolve the solid formed. The solvent was removed using a rotary evaporator, and the organic material was dissolved in dichloromethane (3 × 50 mL). The combined organic layer was washed with a brine solution, dried over Na_2_SO_4_, and concentrated to dryness. The residue was purified using flash column chromatography over silica gel (60–120 mesh) with an ethyl acetate-petroleum ether eluent system to afford the pure desired *N*-phenylacetamide 4a as a white solid, yield: 1.29 g, 96%.

## Author contributions

Priyanka Velmurugan: Writing-original draft, methodology, software, visualization, investigation. Poovarasan Kanniyappan: Contribution to formal analysis. Tapas Ghatak: Writing, reviewing, and editing.

## Conflicts of interest

The authors declare that they have no competing financial interests or personal relationships that could have appeared to influence the work reported in this paper.

## Supplementary Material

RA-015-D5RA02843D-s001

RA-015-D5RA02843D-s002

## Data Availability

Crystallographic data for [1] have been deposited at the CCDC under 2446065 (1) and can be obtained from https://www.ccdc.cam.ac.uk/deposit/. The datasets supporting this article have been uploaded as part of the ESI.†
