# Supplementary material for: Beckmann rearrangement of ketoximes for accessing amides and lactams promoted by a perimidine-2-thione supported Hg(ii) complex: a mechanistic perception
Source: RSC Adv. 2025 Jul 11;15(30):24317–30. doi: 10.1039/d5ra02843d (PMC12247047; doi:10.1039/d5ra02843d)
Supplement: RA-015-D5RA02843D-s001 [file RA-015-D5RA02843D-s001.pdf]

Supplementary Information (SI) for RSC Advances.
This journal is © The Royal Society of Chemistry 2025

Supporting Information

Beckmann Rearrangement of Ketoximes for Accessing Amides and Lactams Promoted by Perimidine-2-thione Supported Hg(II) Complex: A Mechanistic Perception

Priyanka Velmurugan[a], Poovarasan Kanniyappan[a], Tapas Ghatak*[a]

[a]Vellore Institute of Technology, Vellore

[a]Advanced Catalysis Facility,

Department of Chemistry,

School of Advanced Sciences,

Vellore Institute of Technology, Vellore-632014, Tamil Nadu, India.

E-mail: tapaschem@gmail.com; tapas.ghatak@vit.ac.in

Table of contents

1. General procedure for the preparation of oxime substrates (**3a – 3z**) S4

2. General procedure for the synthesis of amides and lactam (**4a – 4z**) S13

Figure S1: ^{1}H and ^{13}C NMR spectrum of Hg(II)Cl_2($\mathbf{N^{i\text{-}Pr}PmT}$)$_2$ complex (**1**) S23

Figure S2: ^{1}H and ^{13}C NMR spectrum of (E)-1-phenylethan-1-one oxime (**3a**) S24

Figure S3: ^{1}H and ^{13}C NMR spectrum of (E)-1-(p-tolyl)ethan-1-one oxime **(3b)** S25

Figure S4: ^{1}H and ^{13}C NMR spectrum of (E)-1-(4-ethylphenyl)ethan-1-one oxime (**3c**) S26

Figure S5: ^{1}H and ^{13}C NMR spectrum of (E)-1-(4-methoxyphenyl)ethan-1-one oxime (**3d**) S27

Figure S6: ^{1}H and ^{13}C NMR spectrum of (E)-1-(3-methoxyphenyl)ethan-1-one oxime (**3e**) S28

Figure S7: ^{1}H and ^{13}C NMR spectrum of (E)-1-(2-methoxyphenyl)ethan-1-one oxime (**3f**) S29

Figure S8: ^{1}H and ^{13}C NMR spectrum of (E)-1-(3,4-dimethoxyphenyl)ethan-1-one oxime (**3g**) S30

Figure S9: ^{1}H and ^{13}C NMR spectrum of (E)-1-(2,4-dimethoxyphenyl)ethan-1-one oxime (**3h**) S31

Figure S10: ^{1}H and ^{13}C NMR spectrum of (E)-1-(4-hydroxyphenyl)ethan-1-one oxime (**3i**) S32

Figure S11: ^{1}H and ^{13}C NMR spectrum of (E)-1-(2-hydroxyphenyl)ethan-1-one oxime (**3j**) S33

Figure S12: ^{1}H and ^{13}C NMR spectrum of (E)-1-(2,4-dihydroxyphenyl)ethan-1-one oxime (**3k**) S34

Figure S13: ^{1}H and ^{13}C NMR spectrum of (E)-1-(4-(trifluoromethyl)phenyl)ethan-1-one oxime(**3l**) S35

Figure S14: ^{1}H and ^{13}C NMR spectrum of (E)-1-(3-chlorophenyl)ethan-1-one oxime (**3m**) S36

Figure S15: ^{1}H and ^{13}C NMR spectrum of 1-(2-chlorophenyl)ethan-1-one oxime (**3n**) S37

Figure S16: ^{1}H and ^{13}C NMR spectrum of 1-(2-iodophenyl)ethan-1-one oxime (**3o**) S38

Figure S17: ^{1}H and ^{13}C NMR spectrum of (E)-1-(thiophen-2-yl)ethan-1-one oxime (**3p'**) S39

Figure S18: ^{1}H and ^{13}C NMR spectrum of (Z)-1-(thiophen-2-yl)ethan-1-one oxime (**3p''**) S40

Figure S19: ^{1}H and ^{13}C NMR Spectrum of (E)-1-(4-chlorophenyl)propan-1-one oxime (**3q**) S41

Figure S20: ^{1}H and ^{13}C NMR Spectrum of (E)-1-Phenylbutan-1-one oxime (**3r**) S42

Figure S21: ^{1}H and ^{13}C NMR Spectrum of 2-Methyl-1-phenylpropan-1-one oxime (**3s**) S43

Figure S22: ^{1}H and ^{13}C NMR Spectrum of cyclohexanone oxime **(3t)** S44

Figure S23: ^{1}H and ^{13}C NMR Spectrum of diphenylmethanone oxime **(3u)** S45

Figure S24: ^{1}H and ^{13}C NMR Spectrum of di-p-tolylmethanone oxime **(3v)** S46

Figure S25: ^{1}H and ^{13}C NMR Spectrum of bis(4-methoxyphenyl)methanone oxime **(3w)** S47

Figure S26: ^{1}H and ^{13}C NMR Spectrum of bis(4-chlorophenyl)methanone oxime **(3x)** S48

Figure S27: ^{1}H and ^{13}C NMR Spectrum of (4-chlorophenyl)(phenyl)methanone oxime **(3y)** S49

Figure S28: ^{1}H and ^{13}C NMR Spectrum of (E)-1-(4-bromophenyl)ethan-1-one oxime **(3z)** S50

Figure S29: ^{1}H and ^{13}C NMR spectrum of N-Phenylacetamide (**4a**) S51

Figure S30: ^{1}H and ^{13}C NMR spectrum of N-p-Tolylacetamide (**4b**) S52

Figure S31: ^{1}H and ^{13}C NMR spectrum of N-(4-Ethylphenyl)acetamide (**4c**) S53

Figure S32: ^{1}H and ^{13}C NMR Spectrum of (N-(4-methoxyphenyl)acetamide) (**4d**) S54

Figure S33: ^{1}H and ^{13}C NMR spectrum of *N*-(3-Methoxyphenyl)acetamide (**4e**) **S55**

Figure S34: ^{1}H and ^{13}C NMR spectrum of N-(2-methoxyphenyl)acetamide (**4f**) **S56**

Figure S35: ^{1}H and ^{13}C NMR spectrum of N-(3,4-dimethoxyphenyl)acetamide (**4g**) **S57**

Figure S36: ^{1}H and ^{13}C NMR spectrum of N-(2,4-dimethoxyphenyl)acetamide (**4h**) **S58**

Figure S37: ^{1}H and ^{13}C NMR spectrum of N-(4-hydroxyphenyl)acetamide (**4i**) **S59**

Figure S38: ^{1}H and ^{13}C NMR spectrum of N-(2-Hydroxyphenyl)acetamide (**4j**) **S60**

Figure S39: ^{1}H and ^{13}C NMR spectrum of N-(2,4-dihydroxyphenyl)acetamide (**4k**) **S61**

Figure S40: ^{1}H and ^{13}C NMR spectrum of N-(4-(trifluoromethyl)phenyl)acetamide (**4l**) **S62**

Figure S41: ^{1}H and ^{13}C NMR spectrum of N-(3-chlorophenyl)acetamide (**4m**) **S63**

Figure S42: ^{1}H and ^{13}C NMR spectrum of N-(2-chlorophenyl)acetamide (**4n**) **S64**

Figure S43: ^{1}H and ^{13}C NMR spectrum of N-(2-iodophenyl)acetamide (**4o**) **S65**

Figure S44: ^{1}H and ^{13}C NMR spectrum of (N-(thiophen-2-yl)acetamide) (**4p**) **S66**

Figure S45: ^{1}H and ^{13}C NMR Spectrum of N-(4-chlorophenyl)propionamide (**4q**) **S67**

Figure S46: ^{1}H and ^{13}C NMR Spectrum of N-Phenylbutyramide (**4r**) **S68**

Figure S47: ^{1}H and ^{13}C NMR Spectrum of N-phenylisobutyramide and N-isopropylbenzamide (**4s**) **S69**

Figure S48: ^{1}H and ^{13}C NMR spectrum of Azepan-2-one (**4t**) **S70**

Figure S49: ^{1}H and ^{13}C NMR spectrum of N-Phenylbenzamide (**4u**) **S71**

Figure S50: ^{1}H and ^{13}C NMR spectrum of (4-methyl-*N*-*p*-tolylbenzamide) (**4v**) **S72**

Figure S51: ^{1}H and ^{13}C NMR spectrum of (4-methyl-*N*-*p*-tolylbenzamide) (**4w**) **S73**

Figure S52: ^{1}H and ^{13}C NMR spectrum of 4-chloro-N-(4-chlorophenyl)benzamide (**4x**) **S74**

Figure S53: ^{1}H and ^{13}C NMR spectrum of N-(4-chlorophenyl)benzamide and 4-chloro-N-phenylbenzamide (**4y**) **S75**

Figure S54: ^{1}H and ^{13}C NMR spectrum of N-(4-bromophenyl)acetamide (**4z**) **S76**

Figure S55: FT-IR spectra of complex **1** and ligand **N$^{i\text{-}Pr}$PmT** **S77**

Scheme S1: The Beckmann rearrangement of (E)-1-phenylethan-1-one O-methyl oxime (**3ah**) under standard conditions using Hg(II) catalyst (**1**) **S78**

Cautions! *Special care should still be taken when using and disposing the waste containing mercury salts.*

1. General procedure for the preparation of oxime substrates (3a – 3z)

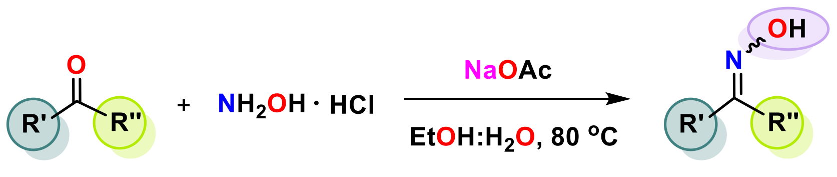

In a 100 mL round-bottom flask equipped with a condenser, aromatic or aliphatic ketones (1 mmol) were dissolved in the mixture of ethanol: water (v/v; 4:1, 20 mL). Then, hydroxylamine hydrochloride (16 mmol, 1.6 equiv) and AcONa (20 mmol, 2.0 equiv) were added in one portion. The reaction was stirred at 80 °C until the consumption of the starting material was observed by TLC. After that, the reaction was cooled to room temperature, diluted with water (55 mL), extracted with ethyl acetate (80 × 3), dried with anhydrous Na_2SO_4, and concentrated *in vacuo*. The residue was purified by recrystallization or flash column chromatography on silica gel to afford the desired oxime products (**3a – 3z**).

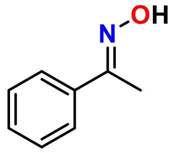

3a

(E)-1-phenylethan-1-one oxime: **3a** was isolated as a white solid, yield: 87%. ^{1}H NMR (400 MHz, $CDCl_3$) δ 7.53 (m, 2H), 7.30 (m, 3H), 2.23 (s, 3H). ^{13}C NMR (100 MHz, $CDCl_3$) δ 156.1, 136.5, 129.3, 128.6, 126.1, 12.5.

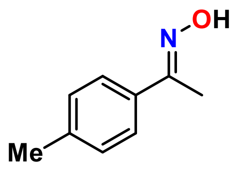

3b

(E)-1-(p-tolyl)ethan-1-one oxime: **3b** was isolated as a pale-yellow solid, yield: 84%. ^{1}H NMR (400 MHz, $CDCl_3$) δ 9.51 (s, 1H), 7.51 (d, J = 8.2 Hz, 2H), 7.18 (d, J = 7.9 Hz, 2H), 2.36 (s, 3H), 2.29 (s, 3H). ^{13}C NMR (100 MHz, $CDCl_3$) δ 156.0, 139.3, 133.7, 129.3, 126.0, 21.3, 12.4.

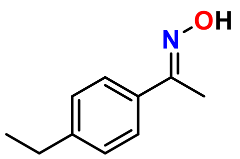

3c

(E)-1-(4-ethylphenyl)ethan-1-one oxime: **3c** was isolated as an off-white solid, yield: 76%. ^{1}H NMR (400 MHz, DMSO) δ 11.09 (s, 1H), 7.56 (d, J = 8.3 Hz, 2H), 7.22 (d, J = 8.4 Hz, 2H), 2.61 (q, J = 7.6 Hz, 2H), 2.13 (s, 3H), 1.18 (t, J = 7.6 Hz, 3H). ^{13}C NMR (100 MHz, DMSO) δ 153.4, 144.8, 134.8, 128.2, 126.0, 28.3, 15.9, 12.0.

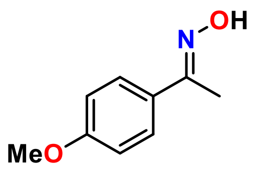

3d

(E)-1-(4-methoxyphenyl)ethan-1-one oxime: **3d** was isolated as a white solid, yield: 86%. ^{1}H NMR (400 MHz, $CDCl_3$) δ 9.04 (s, 1H), 7.61 – 7.53 (m, 2H), 7.06 – 6.87 (m, 2H), 3.83 (s, 3H), 2.28 (s, 3H). ^{13}C NMR (100 MHz, $CDCl_3$) δ 160.6, 155.6, 128.9, 127.5, 114.0, 55.3, 12.5.

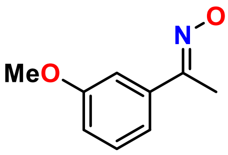

3e

(E)-1-(3-methoxyphenyl)ethan-1-one oxime: **3e** was isolated as a colourless oil, yield: 93%. ^{1}H NMR (400 MHz, $CDCl_3$) δ 9.53 (s, 1H), 7.29 (t, J = 7.9 Hz, 1H), 7.19 (m, 2H), 6.94 – 6.90 (m, 1H), 3.82 (s, 3H), 2.28 (s, 3H). ^{13}C NMR (100 MHz, $CDCl_3$) δ 159.6, 155.8, 138.1, 129.5, 118.7, 115.0, 111.4, 55.3, 12.4.

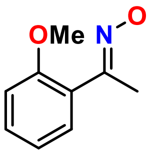

3f

(E)-1-(2-methoxyphenyl)ethan-1-one oxime: **3f** was isolated as a white solid, yield: 79%. ^{1}H NMR (400 MHz, $CDCl_3$) δ 9.68 (s, 1H), 7.34 – 7.30 (m, 1H), 7.28 (t, J = 2.4 Hz, 1H), 6.94 (d, J = 7.5 Hz, 1H), 6.91 – 6.86 (m, 1H), 3.79 (s, 3H), 2.25 (s, 3H). ^{13}C NMR (100 MHz, $CDCl_3$) δ 157.4, 156.7, 130.2, 129.4, 126.8, 120.6, 111.2, 55.4, 15.3.

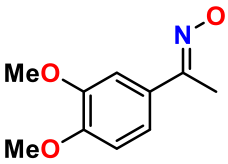

3g

(E)-1-(3,4-dimethoxyphenyl)ethan-1-one oxime: **3g** was isolated as a white solid, yield: 96%.^{1}H NMR (400 MHz, $CDCl_3$) δ 7.26 (s, 1H), 7.16 (d, J = 6.0 Hz, 1H), 6.87 (d, J = 10.6 Hz, 1H), 3.92 (s, 6H), 2.30 (s, 3H).^{13}C NMR (100 MHz, $CDCl_3$) δ 155.7, 150.2, 148.9, 129.2, 119.3, 110.7, 108.6, 55.9, 12.3.

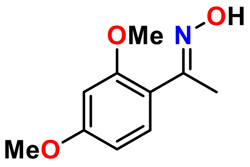

3h

(E)-1-(2,4-dimethoxyphenyl)ethan-1-one oxime: **3h** was isolated as a white solid, yield: 97%. ^{1}H NMR (400 MHz, $CDCl_3$) δ 7.24 (d, J = 9.2 Hz, 1H), 6.47 (s, 2H), 3.81 (s, 6H), 2.21 (s, 3H).^{13}C NMR (100 MHz, $CDCl_3$) δ 161.5, 158.6, 156.5, 130.1, 119.7, 104.3, 99.0, 55.4, 15.2.

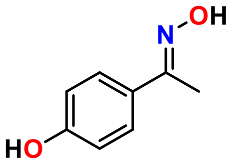

3i

(E)-1-(4-hydroxyphenyl)ethan-1-one oxime: **3i** was isolated as a brown solid, yield: 77%. ^{1}H NMR (400 MHz, DMSO) δ 10.85 (s, 1H), 9.63 (s, 1H), 7.48 (d, J = 8.7 Hz, 2H), 6.77 (d, J = 8.7 Hz, 2H), 2.09 (s, 3H). ^{13}C NMR (100 MHz, DMSO) δ 158.5, 153.0, 128.3, 127.3, 115.6, 11.9.

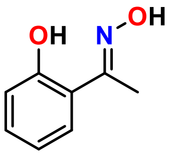

3j

(E)-1-(2-hydroxyphenyl)ethan-1-one oxime: **3j** was isolated as a white solid, yield: 81%. ^{1}H NMR (400 MHz, $CDCl_3$) δ 11.59 (s, 1H), 8.22 (s, 1H), 7.35 (m, 1H), 7.18 (m, 1H), 6.90 (m, 1H), 6.86 – 6.80 (m, 1H), 2.27 (s, 3H). ^{13}C NMR (100 MHz, $CDCl_3$) δ 159.4, 157.4, 130.8, 127.7, 119.4, 118.7, 117.2, 10.8.

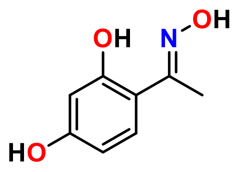

3k

(E)-1-(2,4-dihydroxyphenyl)ethan-1-one oxime: **3k** was isolated as a pale green solid, yield: 95%. ^{1}H NMR (400 MHz, DMSO) δ 11.82 (s, 1H), 11.29 (s, 1H), 9.83 (s, 1H), 7.33 (d, *J* = 8.7 Hz, 1H), 6.37 (d, *J* = 11.5 Hz, 1H), 6.30 (s, 1H), 2.24 (s, 3H).^{13}C NMR (100 MHz, DMSO) δ 159.7, 159.4, 157.9, 129.4, 111.5, 107.3, 103.3, 11.2.

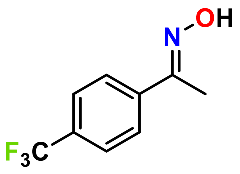

3l

(E)-1-(4-(trifluoromethyl)phenyl)ethan-1-one oxime: **3l** was isolated as a white solid, yield: 94%. ^{1}H NMR (400 MHz, $CDCl_3$) δ 9.53 (s, 1H), 7.76 – 7.68 (m, 2H), 7.68 – 7.59 (m, 2H), 2.32 (s, 3H). ^{13}C NMR (100 MHz, $CDCl_3$) δ 155.2, 139.8, 131.2 (d, *J* = 32.6 Hz), 126.4, 125.5 (q, *J* = 3.7 Hz), 122.6, 12.4.

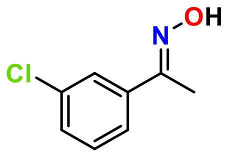

3m

(E)-1-(3-chlorophenyl)ethan-1-one oxime: **3m** was isolated as a white solid, yield: 77%. ^{1}H NMR (400 MHz, $CDCl_3$) δ 9.59 (s, 1H), 7.59 (t, *J* = 1.9 Hz, 1H), 7.49 (dt, *J* = 7.3, 1.6 Hz, 1H), 7.36 – 7.26 (m, 2H), 2.28 (s, 3H). ^{13}C NMR (100 MHz, $CDCl_3$) δ 155.2, 138.2, 134.6, 129.8, 129.3, 126.3, 124.3, 12.5.

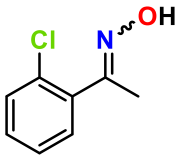

3n

1-(2-chlorophenyl)ethan-1-one oxime: **3n** was isolated as a white solid, yield: 86%. ^{1}H NMR (400 MHz, $CDCl_3$) δ 9.64 (s, 1H), 9.02 (s, 1H), 7.39 (d, *J* = 7.3 Hz, 1H), 7.35 – 7.14 (m, 4H), 2.26 (s, 3H), 2.20 (s, 1H).^{13}C NMR (100 MHz, $CDCl_3$) δ 156.9, 154.3, 136.7, 134.9, 132.6, 130.9, 130.1, 130.0, 129.8, 129.6, 128.2, 126.9, 21.1, 15.8.

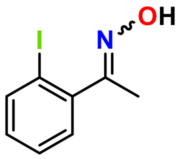

3o

1-(2-iodophenyl)ethan-1-one oxime: (1:0.5) **3o** was isolated as a white solid, yield: 78%. ^{1}H NMR (400 MHz, $CDCl_3$) δ 9.37 (s, 1H), 8.98 (s, 1H), 7.77 (dd, *J* = 8.0, 1.2 Hz, 2H), 7.33 – 7.27 (m, 1H), 7.27 – 7.23 (m, 1H), 7.15 (dd, *J* = 7.7, 1.8 Hz, 1H), 7.01 – 6.96 (m, 1H), 6.94 (dd, *J* = 7.6, 1.7 Hz, 1H), 2.16 (s, 3H), 2.11 (s, 2H).^{13}C NMR (100 MHz, $CDCl_3$) δ 159.4, 157.3, 142.6, 141.8, 139.6, 139.1, 130.1, 129.8, 129.5, 128.2, 128.2, 127.4, 95.8, 93.8, 21.2, 16.4.

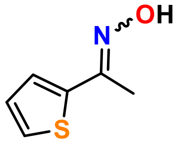

3p

1-(thiophen-2-yl)ethan-1-one oxime: **3p** was isolated as a white solid, yield: 91%. Isomers are separated for characterization. Major isomer (E-isomer, **3p'**): White solid. ^{1}H NMR (400

MHz, $CDCl_3$) δ 9.04 (s, 1H), 7.28 (m, 1H), 7.25 (m, 1H), 7.03 (m, 1H), 2.32 (s, 3H). ^{13}C NMR (100 MHz, $CDCl_3$) δ 151.77, 140.11, 127.21, 126.83, 126.59, 12.48. Minor isomer (Z-isomer, **3p"**): White solid. ^{1}H NMR (400 MHz, $CDCl_3$) δ 7.57 (dd, J = 5.1, 1.2 Hz, 1H), 7.52 (dd, J = 3.9, 1.2 Hz, 1H), 7.11 (dd, J = 5.1, 3.9 Hz, 1H), 2.39 (s, 3H). ^{13}C NMR (100 MHz, $CDCl_3$) δ 147.1, 132.3, 131.1, 129.9, 125.6, 19.7.

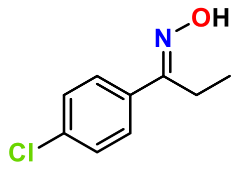

3q

(E)-1-(4-chlorophenyl)propan-1-one oxime: **3q** was isolated as a white solid, yield: 90%. ^{1}H NMR (400 MHz, $CDCl_3$) δ 9.41 (s, 1H), 7.41 (d, J = 8.7 Hz, 2H), 7.23 (d, J = 8.7 Hz, 2H), 2.69 (q, J = 7.6 Hz, 2H), 1.04 (t, J = 7.6 Hz, 3H). ^{13}C NMR (100 MHz, $CDCl_3$) δ 160.0, 135.3, 133.9, 128.9, 127.7, 19.9, 10.8.

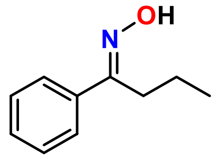

3r

(E)-1-Phenylbutan-1-one oxime: **3r** was isolated as a white solid, yield: 85%. ^{1}H NMR (400 MHz, $CDCl_3$) δ 9.66 (s, 1H), 7.54 – 7.44 (m, 2H), 7.34 – 7.22 (m, 3H), 2.75 – 2.67 (m, 2H), 1.51 (h, J = 7.4 Hz, 2H), 0.88 (t, J = 7.5 Hz, 3H).^{13}C NMR (100 MHz, $CDCl_3$) δ 159.8, 135.9, 129.2, 128.6, 126.4, 28.3, 19.8, 14.3.

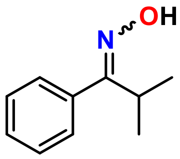

3s

2-Methyl-1-phenylpropan-1-one oxime: **3s** was isolated as a white solid, yield: 96%. ^{1}H NMR (400 MHz, $CDCl_3$) δ 9.23 – 8.02 (brs, 1H), 7.38 – 7.15 (m, 5H), 3.58-3.47 (m, 0.4H), 2.80-2.70 (m, 1H), 1.13 (d, J = 7.1 Hz, 2H), 1.04 (d, J = 6.8 Hz, 3H).^{13}C NMR (100 MHz, $CDCl_3$) δ 164.9, 163.3, 135.8, 133.7, 128.6, 128.5, 128.2, 128.2, 127.8, 127.6, 34.6, 27.7, 20.2, 19.4.

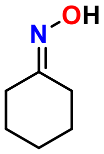

3t

Cyclohexanone oxime: **3t** was isolated as an off-white solid. yield: 95%. ^{1}H NMR (400 MHz, $CDCl_3$) δ 9.66 (s, 1H), 2.51 (t, J = 6.1 Hz, 2H), 2.28 – 2.15 (m, 2H), 1.71 – 1.55 (m, 6H). ^{13}C NMR (100 MHz, $CDCl_3$) δ 160.7, 32.1, 26.9, 25.8, 25.6, 24.5.

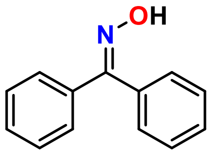

3u

Diphenylmethanone oxime: **3u** was isolated as a white solid, yield: 91%.^{1}H NMR (400 MHz, $CDCl_3$) δ 9.05 (s, 1H), 7.43 – 7.37 (m, 5H), 7.36 – 7.34 (m, 2H), 7.32 – 7.27 (m, 1H), 7.24 (m, 2H). ^{13}C NMR (100 MHz, $CDCl_3$) δ 157.9, 136.2, 132.8, 129.6, 129.3, 129.2, 128.4, 128.3, 128.0.

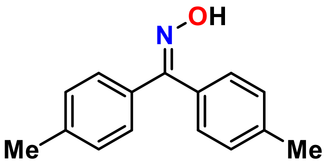

3v

Di-p-tolylmethanone oxime: **3v** was isolated as a white solid, yield: 85%. ^{1}H NMR (400 MHz, $CDCl_3$) δ 9.73 (s, 1H), 7.24 (dd, *J* = 8.2, 4.4 Hz, 4H), 7.16 (d, *J* = 8.1 Hz, 2H), 7.01 (d, *J* = 8.2 Hz, 2H), 2.30 (s, 3H), 2.23 (s, 3H). ^{13}C NMR (100 MHz, $CDCl_3$) δ 157.9, 139.6, 139.1, 133.6, 129.9, 129.3, 129.1, 128.9, 127.9, 21.5, 21.3.

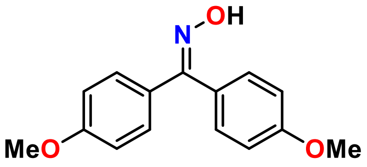

3w

Bis(4-chlorophenyl)methanone oxime: **3w** was isolated as a white solid, yield: 93%. ^{1}H NMR (400 MHz, $CDCl_3$) δ 8.06 (s, 1H), 7.45 – 7.40 (m, 2H), 7.40 – 7.35 (m, 2H), 6.97 (d, *J* = 8.9 Hz, 2H), 6.85 (d, *J* = 9.0 Hz, 2H), 3.84 (s, 3H), 3.80 (s, 3H). ^{13}C NMR (100 MHz, $CDCl_3$) δ 160.7, 160.1, 157.0, 131.2, 129.6, 129.1, 125.0, 113.8, 113.6, 55.4, 55.3.

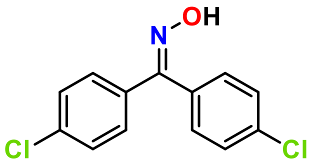

3x

Bis(4-chlorophenyl)methanone oxime: **3x** was isolated as a white solid, yield: 73%. ^{1}H NMR (400 MHz, $CDCl_3$) δ 7.44 (d, *J* = 8.6 Hz, 2H), 7.35 (d, *J* = 7.2 Hz, 4H), 7.30 (d, *J* = 8.7 Hz, 2H). ^{13}C NMR (100 MHz, $CDCl_3$) δ 156.1, 136.0, 135.5, 134.3, 130.9, 130.4, 129.2, 128.8, 128.7.

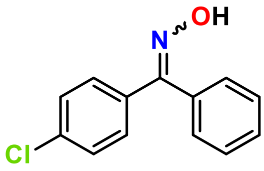

3y

(4-chlorophenyl)(phenyl)methanone oxime: **3y** was isolated as a white solid, yield: 91%. ^{1}H NMR (400 MHz, CDCl$_3$) δ 8.31 (s, 2H), 7.37 (dd, *J* = 7.7, 2.6 Hz, 2H), 7.34 (d, *J* = 4.3 Hz, 2H), 7.32 (t, *J* = 2.4 Hz, 3H), 7.29 (d, *J* = 2.4 Hz, 3H), 7.29 – 7.26 (m, 3H), 7.25 (s, 1H), 7.23 (d, *J* = 1.8 Hz, 1H), 7.21 (q, *J* = 1.8 Hz, 1H), 7.18 (d, *J* = 2.0 Hz, 1H). ^{13}C NMR (100 MHz, CDCl$_3$) δ 155.8, 155.7, 134.7, 134.6, 134.2, 133.6, 131.1, 129.9, 129.9, 128.7, 128.4, 128.2, 128.1, 127.6, 127.5, 127.3, 126.9.

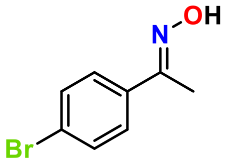

3z

(E)-1-(4-bromophenyl)ethan-1-one oxime: **3z** was isolated as a pale-yellow solid. yield: 90%. ^{1}H NMR (400 MHz, CDCl$_3$) δ 8.61 (s, 1H), 7.52 – 7.34 (m, 4H), 2.20 (s, 3H). ^{13}C NMR (100 MHz, CDCl$_3$) δ 155.2, 135.4, 131.7, 127.6, 123.6, 12.2.

2. General procedures for the synthesis of amides and lactams (4a – 4z)

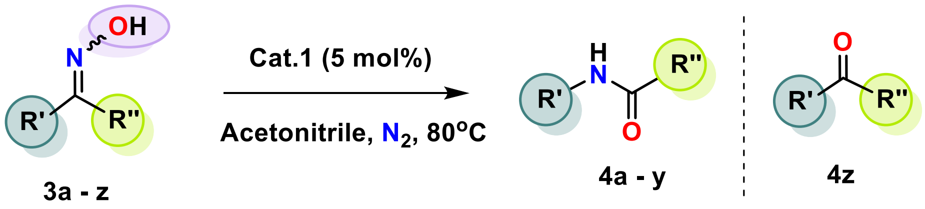

To an oven-dried round bottom flask charged with a solution of corresponding oximes (1 mmol in 5 mL of Acetonitrile) equipped with a reflux condenser and magnetic stirring bar in

the presence of nitrogen atmosphere, was added complex **1** (0.05 mmol, 5 mol%) After being stirred at 80 °C for 12 h, the reaction mixture was allowed to cool to room temperature. The completion of the reaction was identified by monitoring TLC, and then 5 mL of acetonitrile was added to dissolve the solid formed. The solvent was removed using a rotary evaporator, and the organic material was dissolved in dichloromethane (3×15 mL). The combined organic layer was washed with brine solution, dried over Na_2SO_4, and concentrated to dryness. The residue was purified using flash column chromatography over silica gel (60-120 mesh) with an ethyl acetate-petroleum ether eluent system to afford respective amides or lactams.

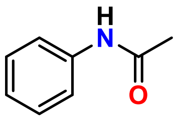

4a

N-Phenylacetamide: **4a** was isolated as a white solid, yield: 95%.^{1}H NMR (400 MHz, $CDCl_3$) δ 7.97 (s, 1H), 7.42 (d, J = 7.7 Hz, 2H), 7.22 – 7.18 (m, 2H), 7.00 (t, J = 7.5 Hz, 1H), 2.05 (s, 3H). ^{13}C NMR (100 MHz, $CDCl_3$) δ 169.0, 138.0, 128.9, 124.3, 120.1, 24.5.

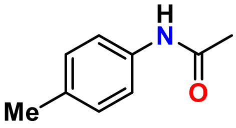

4b

N-p-Tolylacetamide: **4b** was isolated as a white solid, yield: 91%. ^{1}H NMR (400 MHz, $CDCl_3$) δ 7.88 (s, 1H), 7.29 (d, J = 8.6 Hz, 2H), 7.00 (d, J = 8.1 Hz, 2H), 2.21 (s, 3H), 2.04 (s, 3H). ^{13}C NMR (100 MHz, $CDCl_3$) δ 169.0, 135.5, 133.9, 129.4, 120.4, 24.3, 20.9.

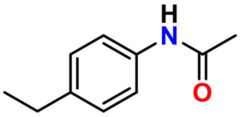

4c

N-(4-Ethylphenyl)acetamide: **4c** was isolated as a white solid, yield: 89%. ^{1}H NMR (400 MHz, DMSO) δ 9.84 (s, 1H), 7.49 (d, J = 8.4 Hz, 2H), 7.11 (d, J = 8.7 Hz, 2H), 2.53 (q, J =

7.2 Hz, 2H), 2.03 (s, 3H), 1.14 (t, J = 7.6 Hz, 3H). ^{13}C NMR (100 MHz, DMSO) δ 168.5, 138.8, 137.5, 128.3, 119.6, 28.1, 24.4, 16.2.

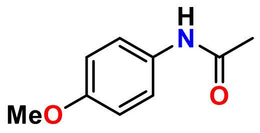

4d

(N-(4-methoxyphenyl)acetamide): 4d was isolated as an off-white solid, yield: 93%.^{1}H NMR (400 MHz, DMSO) δ 9.85 (s, 1H), 7.56 (d, *J* = 9.0 Hz, 2H), 6.94 (d, *J* = 9.0 Hz, 2H), 3.79 (s, 3H), 2.09 (s, 3H). ^{13}C NMR (100 MHz, DMSO) δ 168.2, 155.5, 133.0, 121.0, 114.2, 55.6, 24.2.

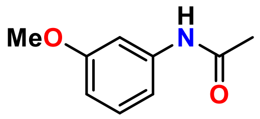

4e

***N*-(3-Methoxyphenyl)acetamide**: **4e** was isolated as a white solid, yield: 85%. ^{1}H NMR (400 MHz, $CDCl_3$) δ 7.85 (s, 1H), 7.18 (t, J = 3.0 Hz, 1H), 7.10 (t, J = 8.2 Hz, 1H), 6.91 (d, J = 8.1 Hz, 1H), 6.57 (m, 1H), 3.69 (s, 3H), 2.07 (s, 3H). ^{13}C NMR (100 MHz, $CDCl_3$) δ 168.9, 160.1, 139.2, 129.6, 112.2, 110.0, 105.9, 55.3, 24.6.

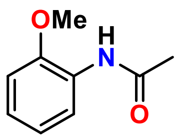

4f

N-(2-methoxyphenyl)acetamide: 4f was isolated as a white solid, yield: 81%.^{1}H NMR (400 MHz, DMSO) δ 9.11 (s, 1H), 7.94 (d, *J* = 8.7 Hz, 1H), 7.06 (m, 1H), 7.01 (m, 1H), 6.88 (td, *J* = 7.2, 1.8 Hz, 1H), 3.82 (s, 3H), 2.08 (s, 3H). ^{13}C NMR (100 MHz, DMSO) δ 168.9, 150.0, 127.9, 124.6, 122.4, 120.6, 111.5, 56.0, 24.3.

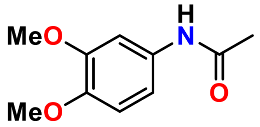

4g

N-(3,4-dimethoxyphenyl)acetamide: 4g was isolated as a white solid, yield: 96%. ^{1}H NMR (400 MHz, $CDCl_3$) δ 7.83 (s, 1H), 7.21 (s, 1H), 6.82 (d, *J* = 3.4 Hz, 1H), 6.69 (d, *J* = 6.2 Hz, 1H), 3.74 (d, *J* = 5.7 Hz, 6H), 2.06 (s, 3H).^{13}C NMR (100 MHz, $CDCl_3$) δ 168.8, 148.9, 145.8, 131.7, 112.2, 111.3, 105.2, 56.1, 55.8, 24.3.

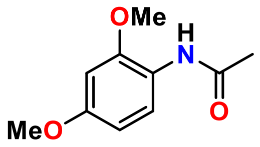

4h

N-(2,4-dimethoxyphenyl)acetamide: 4h was isolated as a brown solid, yield: 91%.^{1}H NMR (400 MHz, DMSO) δ 9.02 (s, 1H), 7.68 (d, J = 8.7 Hz, 1H), 6.59 (d, J = 2.7 Hz, 1H), 6.46 (dd, J = 8.7, 2.7 Hz, 1H), 3.79 (s, 3H), 3.73 (s, 3H), 2.05 (s, 3H). ^{13}C NMR (100 MHz, DMSO) δ 168.8, 157.2, 151.9, 124.4, 120.8, 104.4, 99.1, 56.0, 55.7, 23.9.

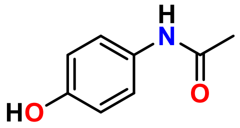

4i

N-(4-hydroxyphenyl)acetamide/Paracetamol: **4i** was isolated as a reddish-orange solid, yield: 90%. ^{1}H NMR (400 MHz, DMSO) δ 9.64 (s, 1H), 9.13 (s, 1H), 7.33 (d, J = 8.8 Hz, 2H), 6.67 (d, J = 8.9 Hz, 2H), 1.98 (s, 3H). ^{13}C NMR (100 MHz, DMSO) δ 168.1, 153.6, 131.4, 121.4, 115.5, 24.1.

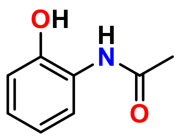

4j

N-(2-Hydroxyphenyl)acetamide: **4j** was isolated as a brown solid, yield: 78%. ^{1}H NMR (400 MHz, DMSO) δ 7.66 – 7.63 (m, 1H), 7.62 – 7.59 (m, 1H), 7.37 – 7.32 (m, 1H), 7.31 – 7.28 (m, 1H), 2.59 (s, 3H). ^{13}C NMR (100 MHz, DMSO) δ 169.7, 148.4, 126.8, 125.3, 122.9, 119.5, 116.5, 40.4, 40.2, 24.0.

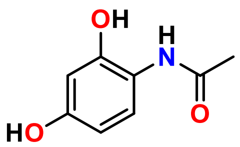

4k

N-(2,4-dihydroxyphenyl)acetamide: 4k was isolated as a reddish brown solid, yield: 71%. ^{1}H NMR (400 MHz, DMSO) δ 9.68 (s, 1H), 9.28 (s, 2H), 7.26 (d, *J* = 8.6 Hz, 1H), 6.36 (d, *J* = 2.7 Hz, 1H), 6.24 (dd, *J* = 8.6, 2.6 Hz, 1H), 2.08 (s, 3H).^{13}C NMR (100 MHz, DMSO) δ 169.4, 155.6, 150.3, 124.8, 118.4, 106.4, 103.8, 23.6.

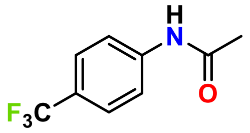

4l

N-(4-(trifluoromethyl)phenyl)acetamide: 4l was isolated as a white solid, yield: 92%. ^{1}H NMR (400 MHz, DMSO) δ 10.31 (s, 1H), 7.79 (d, *J* = 8.4 Hz, 2H), 7.65 (d, *J* = 8.6 Hz, 2H), 2.09 (s, 3H). ^{13}C NMR (100 MHz, DMSO) δ 169.5, 143.3, 126.5, 119.3, 24.6.

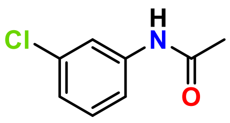

4m

***N*-(3-Chlorophenyl)acetamide: 4m** was isolated as a white solid, yield: 91%. ^{1}H NMR (400 MHz, DMSO) δ 10.07 (s, 1H), 7.75 (s, 1H), 7.36 (dd, *J* = 8.2, 1.1 Hz, 1H), 7.24 (t, *J* = 8.1 Hz, 1H), 7.01 (dd, *J* = 7.9, 1.1 Hz, 1H), 2.00 (s, 3H).^{13}C NMR (100 MHz, DMSO) δ 169.2, 141.6, 133.5, 130.8, 123.2, 118.9, 117.8, 24.5.

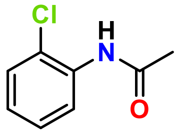

4n

N-(2-chlorophenyl)acetamide: 4n was isolated as a white solid, yield: 89%.^{1}H NMR (400 MHz, DMSO) δ 9.52 (s, 1H), 7.63 (dd, *J* = 47.6, 8.8 Hz, 1H), 7.41 (d, *J* = 16.8 Hz, 1H), 7.37 (m, 1H), 7.25 – 7.10 (m, 1H), 2.07 (d, *J* = 5.1 Hz, 3H).^{13}C NMR (100 MHz, DMSO) δ 170.7, 134.7, 129.9, 127.8, 127.2, 126.8, 122.9, 23.5.

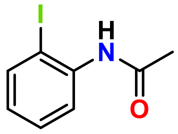

4o

N-(2-iodophenyl)acetamide: 4o was isolated as a white solid, yield: 79%. ^{1}H NMR (400 MHz, DMSO) δ 9.48 (s, 1H), 7.92 (d, *J* = 6.5 Hz, 1H), 7.46 (dd, *J* = 7.9, 2.1 Hz, 1H), 7.42 (t, *J* = 7.5 Hz, 1H), 7.03 (t, *J* = 6.4 Hz, 1H), 2.11 (s, 3H). ^{13}C NMR (100 MHz, DMSO) δ 168.9, 140.2, 139.3, 129.1, 128.1, 128.0, 97.1, 23.7.

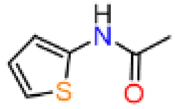

4p

(N-(thiophen-2-yl)acetamide): 4p was isolated as a pale brown solid, yield: 76%.^{1}H NMR (400 MHz, DMSO) δ 11.18 (s, 1H), 6.95 (d, J = 4.9 Hz, 1H), 6.88 (m, 1H), 6.67 (m, 1H), 2.10 (s, 3H). ^{13}C NMR (100 MHz, DMSO) δ 166.7, 140.3, 124.3, 117.1, 110.7, 40.6, 40.3, 40.1, 39.9, 39.7, 39.5, 39.3, 23.0.

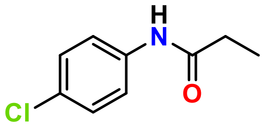

4q

N-(4-Chlorophenyl)propionamide: **4q** was isolated as a yellow solid, yield: 87%. ^{1}H NMR (400 MHz, $CDCl_3$) δ 7.58 (s, 1H), 7.39 (d, J = 8.8 Hz, 2H), 7.21 – 7.14 (m, 2H), 2.30 (q, J = 7.6 Hz, 2H), 1.15 (t, J = 7.5 Hz, 3H). ^{13}C NMR (100 MHz, $CDCl_3$) δ 172.5, 136.6, 129.0, 121.2, 30.6, 9.6.

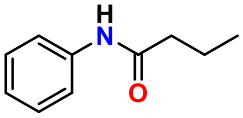

4r

N-Phenylbutyramide: **4r** was isolated as a white solid, yield: 72%. ^{1}H NMR (400 MHz, $CDCl_3$) δ 7.71 (s, 1H), 7.44 (d, J = 7.5 Hz, 2H), 7.21 (t, J = 7.9 Hz, 2H), 7.01 (t, J = 7.5 Hz, 1H), 2.24 (t, J = 7.5 Hz, 2H), 1.66 (m, 2H), 0.90 (t, J = 7.4 Hz, 3H). ^{13}C NMR (100 MHz, $CDCl_3$) δ 171.9, 138.0, 129.0, 124.3, 120.2, 39.6, 19.2, 13.8.

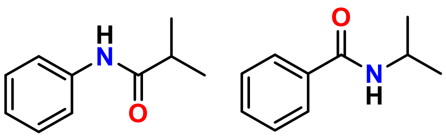

4s

N-Phenylisobutyramide and N-Isopropylbenzamide: (1:2.2), **4s** was isolated as a white solid, yield: 83%. ^{1}H NMR (400 MHz, DMSO) δ 9.79 (s, 1H), 8.20 (s, 0.45H), 7.83 (d, *J* = 7.0 Hz, 1H), 7.60 (d, *J* = 8.1 Hz, 2H), 7.48 (t, *J* = 7.2 Hz, 1H), 7.41 (t, *J* = 7.2 Hz, 1H), 7.26 (t, *J* = 7.9 Hz, 2H), 6.99 (t, *J* = 7.4 Hz, 1H), 4.15-4.05 (m, 0.57H), 2.63-2.52 (m, 1H), 1.15 (d, *J* = 6.6 Hz, 3H), 1.08 (d, *J* = 6.8 Hz, 6H). ^{13}C NMR (100 MHz, DMSO) δ 175.7, 165.9, 139.9, 135.3, 131.4, 129.1, 128.6, 127.7, 123.4, 119.6, 41.5, 35.4, 22.8, 20.0.

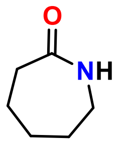

4t

Azepan-2-one: **4t** was isolated as an off-white solid, yield: 92%. ^{1}H NMR (400 MHz, $CDCl_3$) δ 6.82 (s, 1H), 3.16 – 3.12 (m, 2H), 2.39 – 2.33 (m, 2H), 1.69 – 1.66 (m, 2H), 1.62 – 1.57 (m, 4H). ^{13}C NMR (100 MHz, $CDCl_3$) δ 178.5, 41.8, 35.7, 29.6, 28.7, 22.2.

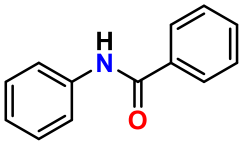

4u

N-Phenylbenzamide: **4u** was isolated as a white solid, yield: 97%. ^{1}H NMR (400 MHz, DMSO) δ 10.26 (s, 1H), 7.98 – 7.95 (m, 2H), 7.80 (d, J = 7.3 Hz, 2H), 7.61 – 7.58 (m, 1H), 7.54 (t, J = 7.2 Hz, 2H), 7.38 – 7.34 (m, 2H), 7.11 (t, J = 7.4 Hz, 1H). ^{13}C NMR (100 MHz, DMSO) δ 166.0, 139.7, 135.5, 132.0, 129.1, 128.8, 128.1, 124.1, 120.8.

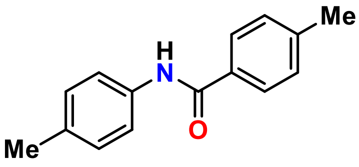

4v

(4-methyl-*N*-*p*-tolylbenzamide): 4v was isolated as an off-white solid, yield: 94%. ^{1}H NMR (400 MHz, DMSO) δ 10.01 (s, 1H), 7.80 (d, *J* = 8.3 Hz, 2H), 7.60 (d, *J* = 8.4 Hz, 2H), 7.24 (d, *J* = 7.9 Hz, 2H), 7.07 (d, *J* = 8.1 Hz, 2H), 2.30 (s, 3H), 2.20 (s, 3H). ^{13}C NMR (100 MHz, DMSO) δ 165.6, 141.9, 137.2, 132.9, 132.6, 129.4, 129.3, 128.1, 120.9, 21.5, 21.0.

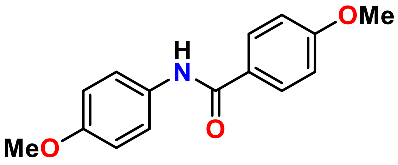

4w

4-Methoxy-N-(4-methoxyphenyl)benzamide: 4w was isolated as a pale yellow solid, yield: 95%. ^{1}H NMR (400 MHz, DMSO) δ 10.03 (s, 1H), 7.99 (d, *J* = 8.8 Hz, 2H), 7.70 (d, *J* = 9.0 Hz, 2H), 7.09 (d, *J* = 8.8 Hz, 2H), 6.96 (d, *J* = 9.2 Hz, 2H), 3.87 (s, 3H), 3.78 (s, 3H). ^{13}C NMR (100 MHz, DMSO) δ 165.0, 162.2, 155.9, 132.8, 129.9, 127.5, 122.5, 114.2, 114.0, 55.9, 55.6.

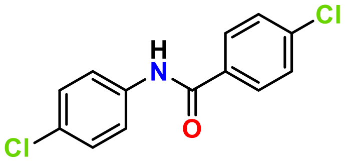

4x

4-chloro-N-(4-chlorophenyl)benzamide: 4x was isolated as a white solid, yield: 89%. ^{1}H NMR (400 MHz, DMSO) δ 10.45 (s, 1H), 8.00 (d, *J* = 8.6 Hz, 2H), 7.83 (d, *J* = 8.9 Hz, 2H), 7.61 (d, *J* = 8.7 Hz, 2H), 7.42 (d, *J* = 8.9 Hz, 2H). ^{13}C NMR (100 MHz, DMSO) δ 165.0, 138.4, 137.1, 133.8, 130.1, 129.0, 128.9, 128.0, 122.4.

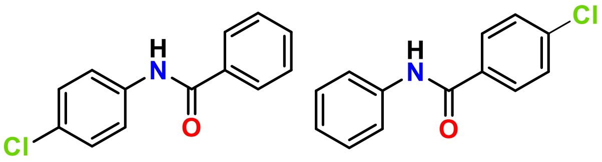

4y

N-(4-chlorophenyl)benzamide and 4-chloro-N-phenylbenzamide: (1:1.4), **4y** was isolated as a white solid, yield: 84%. ^{1}H NMR (400 MHz, $CDCl_3$+DMSO) δ 9.46 (s, 1H), 9.36 (s, 1H), 7.95 (s, 1H), 7.93 (d, J = 2.6 Hz, 2H), 7.91 (s, 1H), 7.75 – 7.71 (m, 3H), 7.57 – 7.50 (m, 1H), 7.48 (d, J = 7.8 Hz, 1H), 7.43 (d, J = 8.7 Hz, 2H), 7.36 (d, J = 7.5 Hz, 2H), 7.30 (d, J = 8.9 Hz, 1H), 7.13 (t, J = 7.4 Hz, 1H). ^{13}C NMR (100 MHz, $CDCl_3$+DMSO) δ 161.5, 160.4, 133.6, 133.0, 132.5, 130.2, 128.8, 127.0, 124.3, 124.1, 124.0, 123.9, 123.8, 122.8, 122.7, 119.7, 117.0, 115.9.

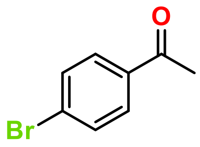

4z

1-(4-bromophenyl)ethan-1-one: **4z** was isolated as an off-white solid, yield: 99%. ^{1}H NMR (400 MHz, DMSO) δ 7.87 (d, J = 8.6 Hz, 2H), 7.71 (d, J = 8.6 Hz, 2H), 2.56 (s, 3H). ^{13}C NMR (100 MHz, DMSO) δ 197.6, 136.2, 132.2, 130.6, 127.8, 27.1.

3. NMR Spectra

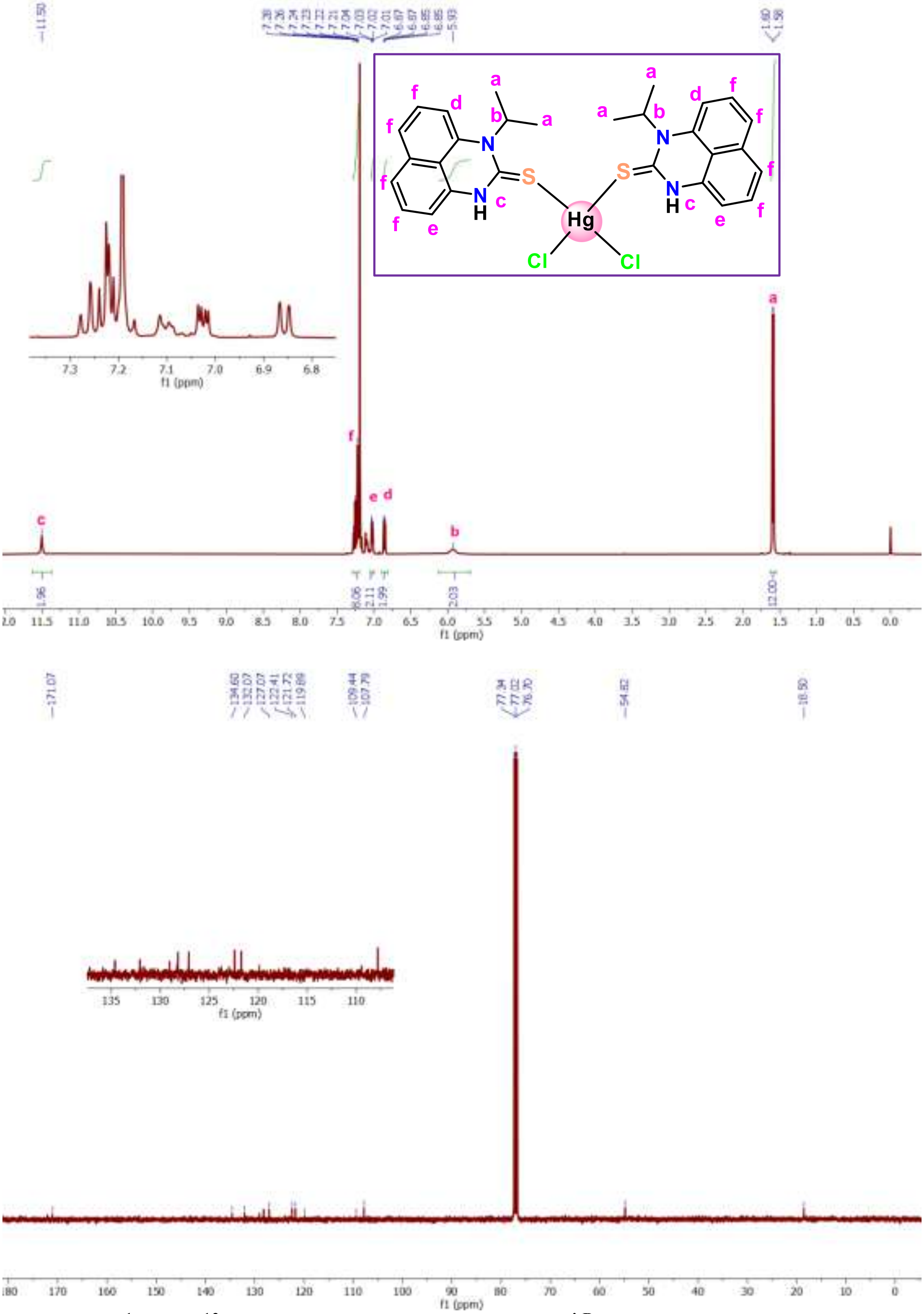

Figure S1: ^{1}H and ^{13}C NMR Spectrum of Hg(II)Cl_2(**N^{i-Pr}PmT**)$_2$ complex (**1**).

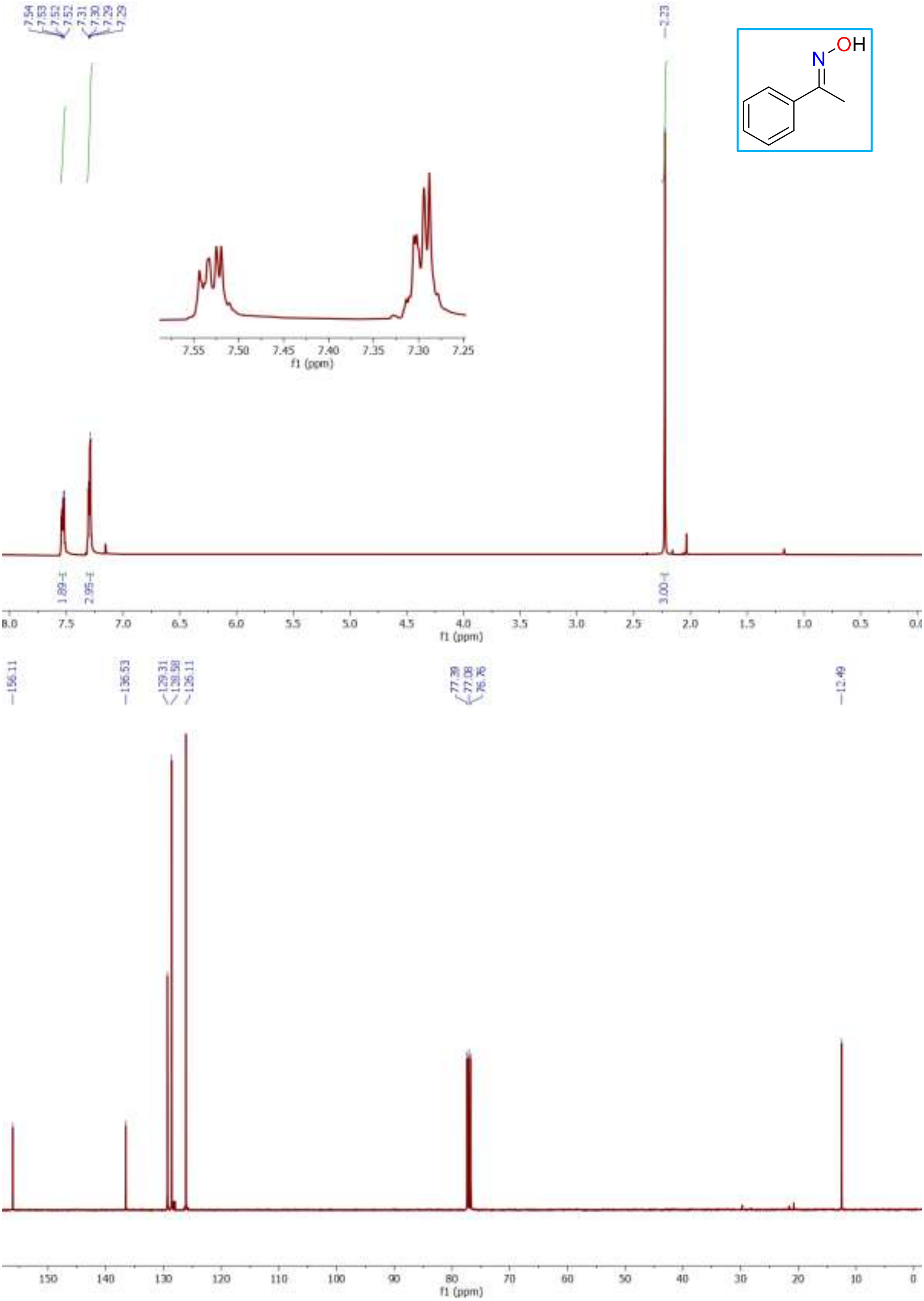

Figure S2: ^{1}H and ^{13}C NMR Spectrum of (E)-1-phenylethan-1-one oxime (**3a**).

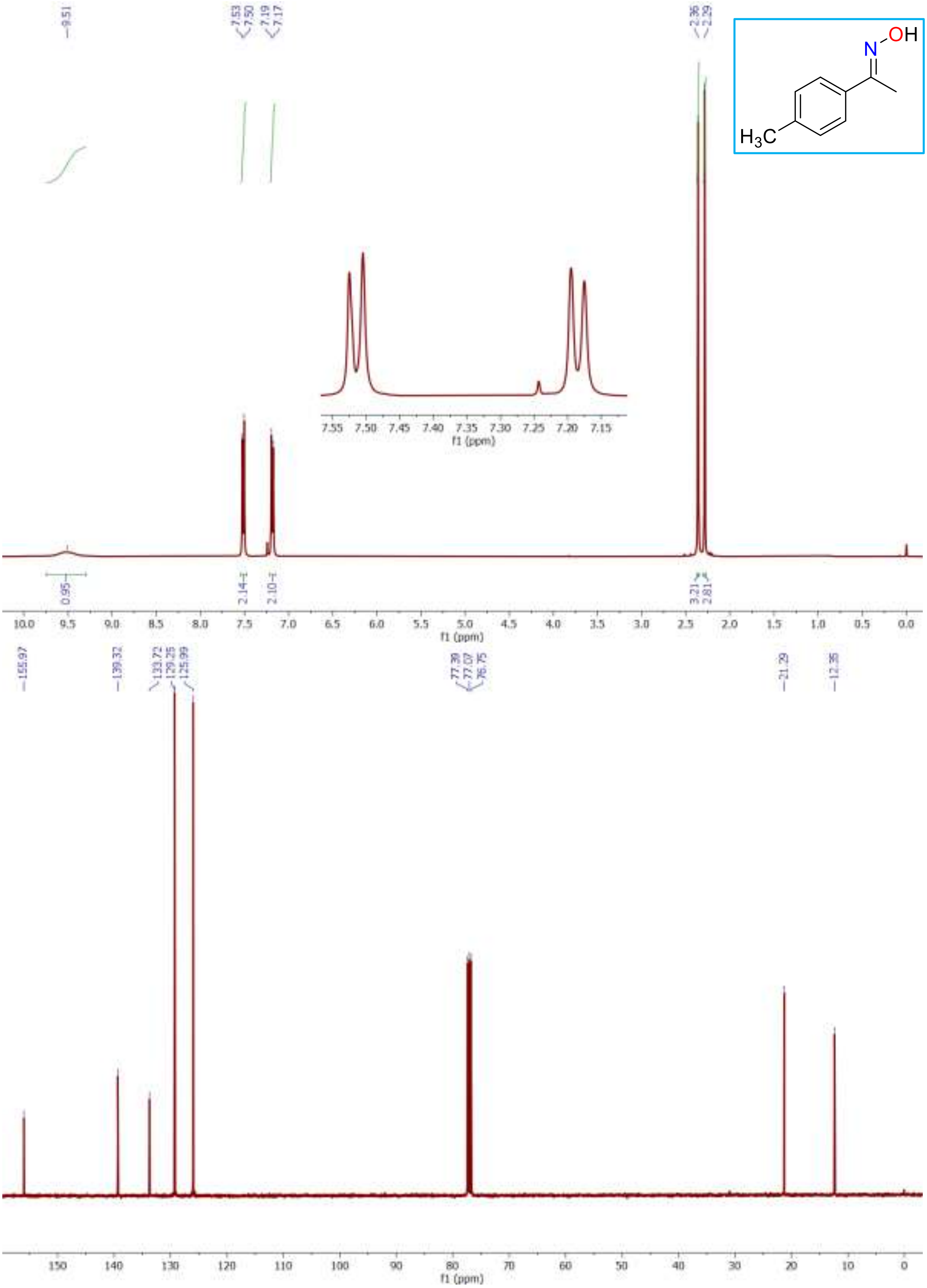

Figure S3: ^{1}H and ^{13}C NMR Spectrum of (E)-1-(p-tolyl)ethan-1-one oxime (**3b**).

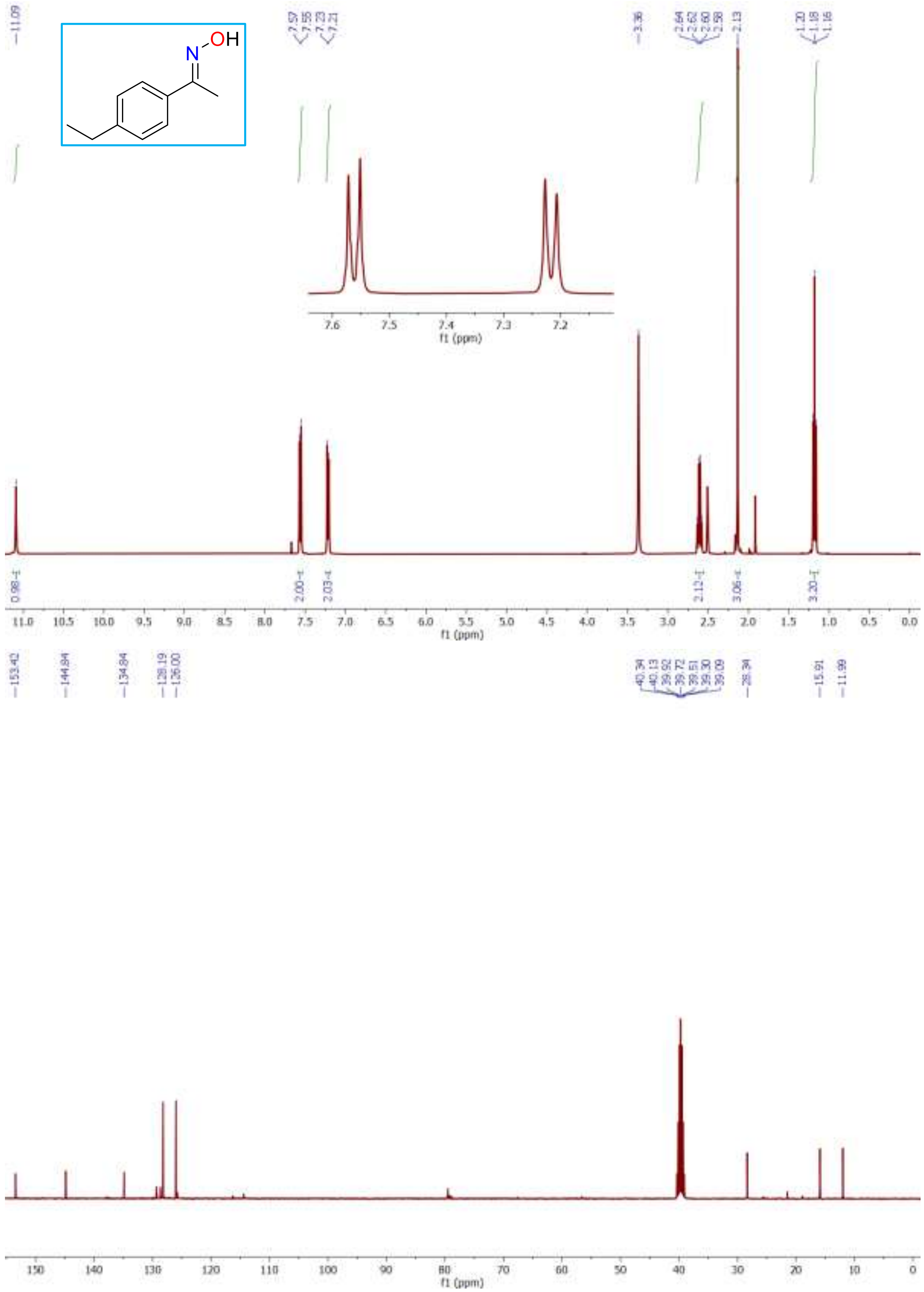

Figure S4: ^{1}H and ^{13}C NMR Spectrum of (E)-1-(4-ethylphenyl)ethan-1-one oxime (**3c**).

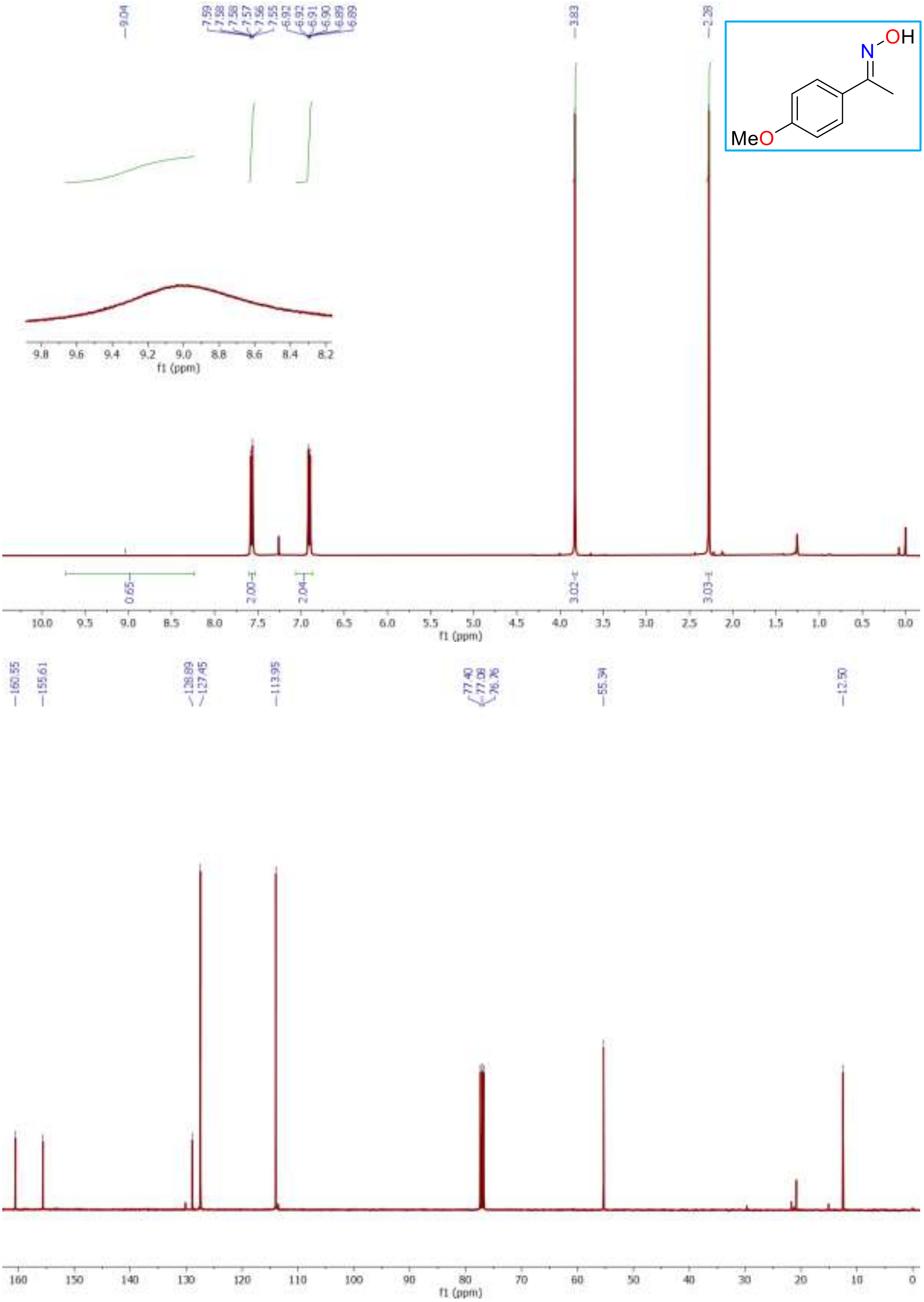

Figure S5: ^{1}H and ^{13}C NMR Spectrum of (E)-1-(4-methoxyphenyl)ethan-1-one oxime (**3d**).

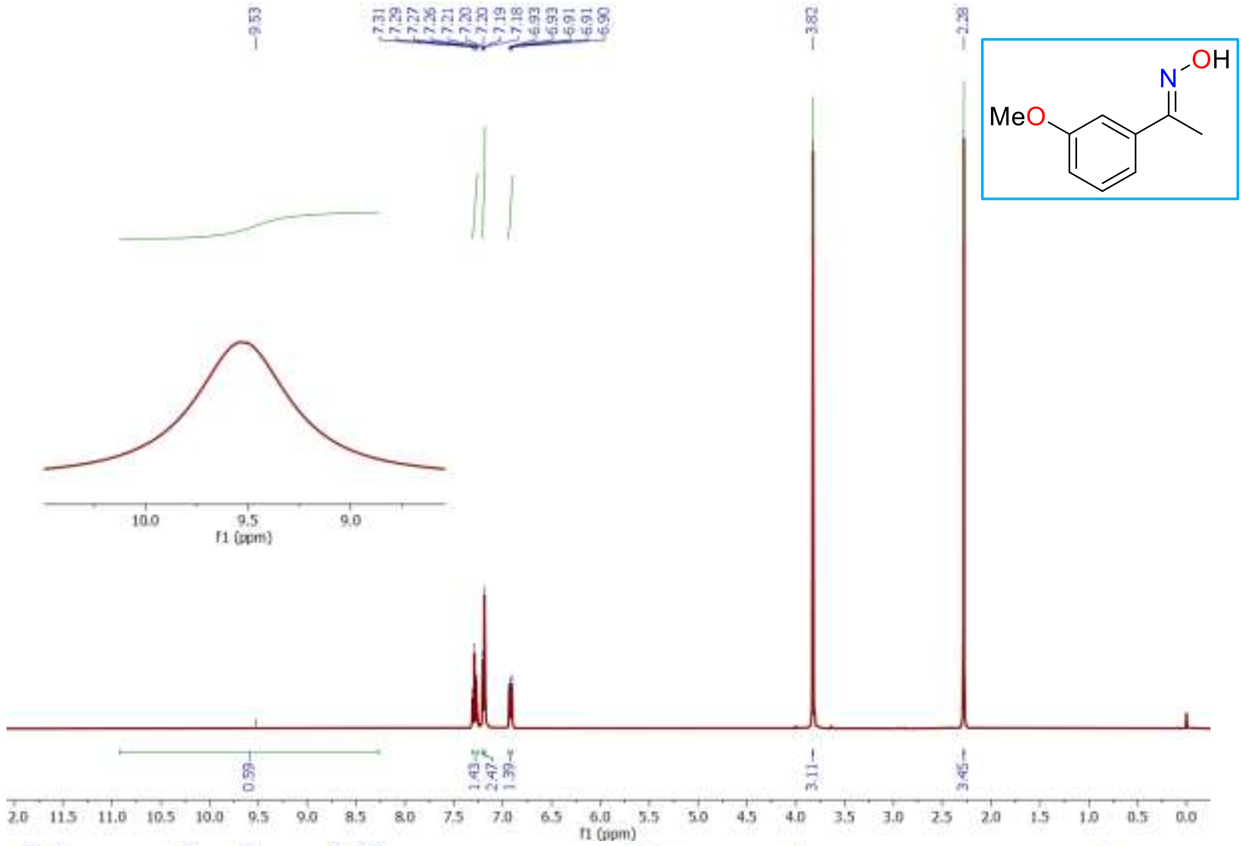

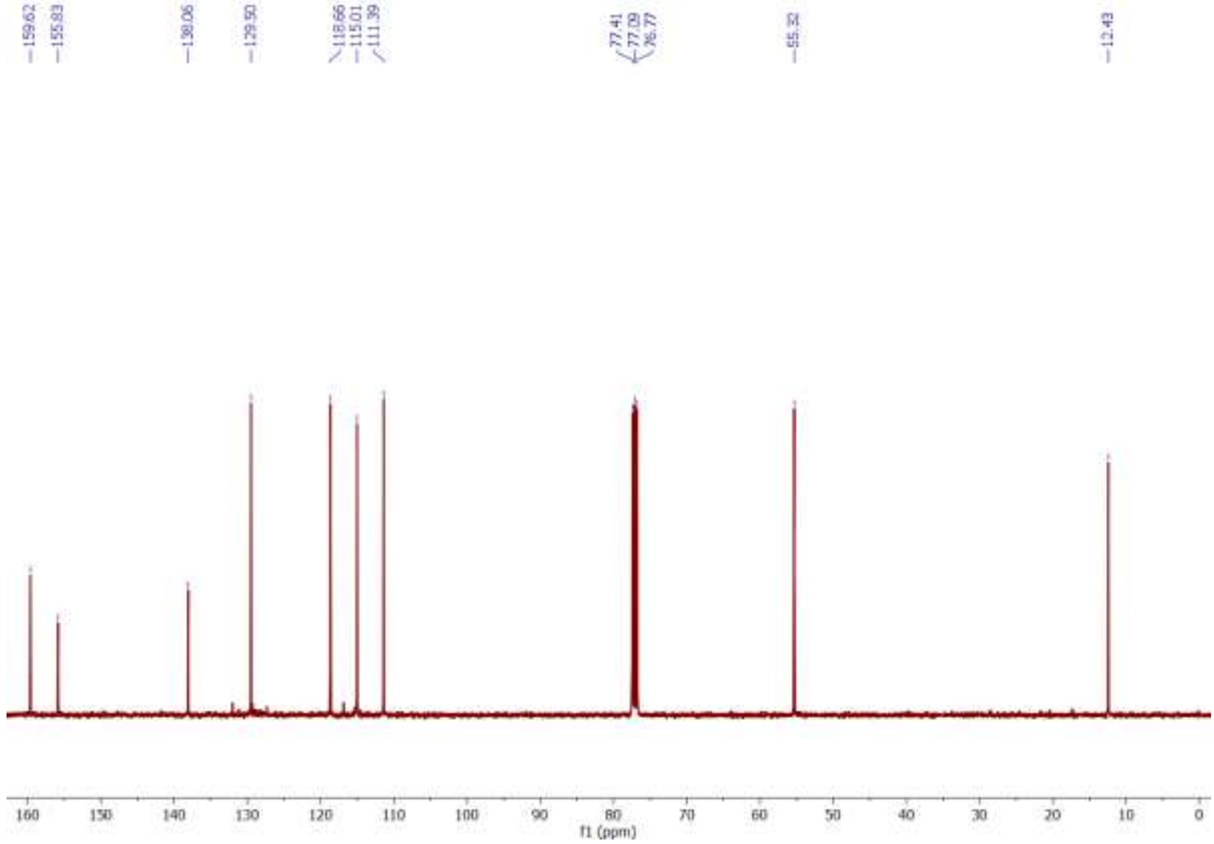

Figure S6: ^{1}H and ^{13}C NMR Spectrum of (E)-1-(3-methoxyphenyl)ethan-1-one oxime (**3e**).

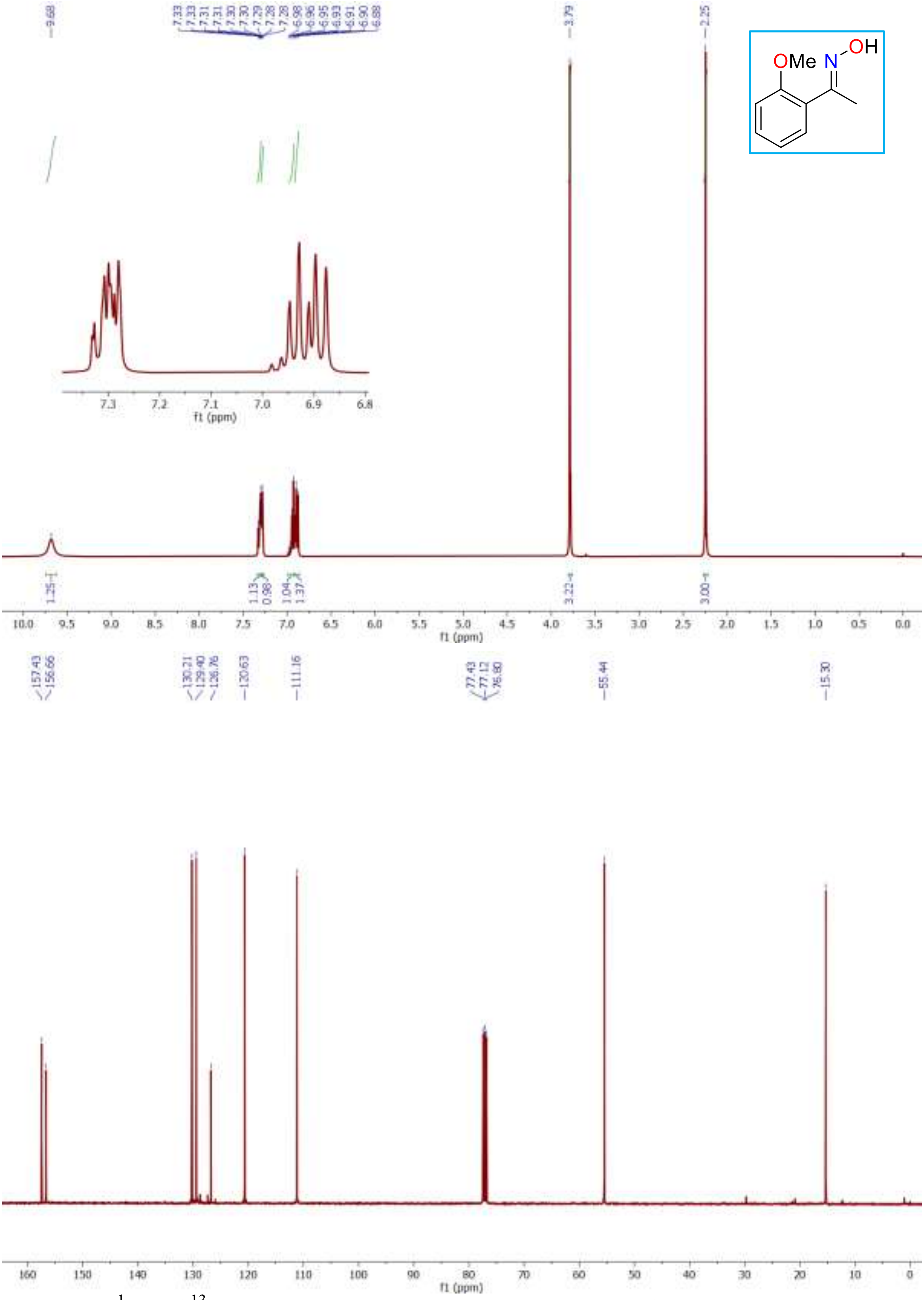

Figure S7: ^{1}H and ^{13}C NMR Spectrum of (E)-1-(2-methoxyphenyl)ethan-1-one oxime (**3f**).

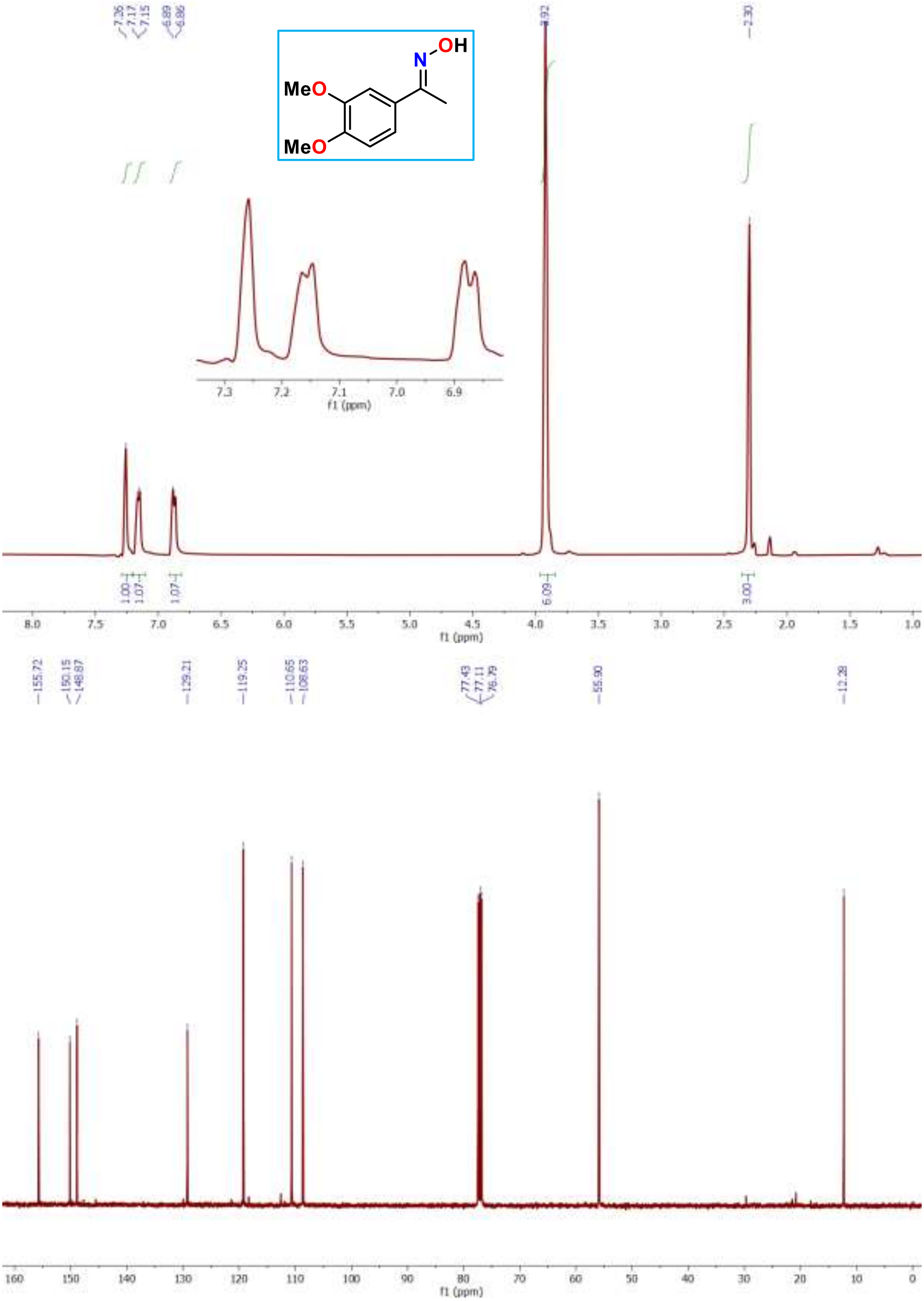

Figure S8: ^{1}H and ^{13}C NMR Spectrum of (E)-1-(3,4-dimethoxyphenyl)ethan-1-one oxime (**3g**).

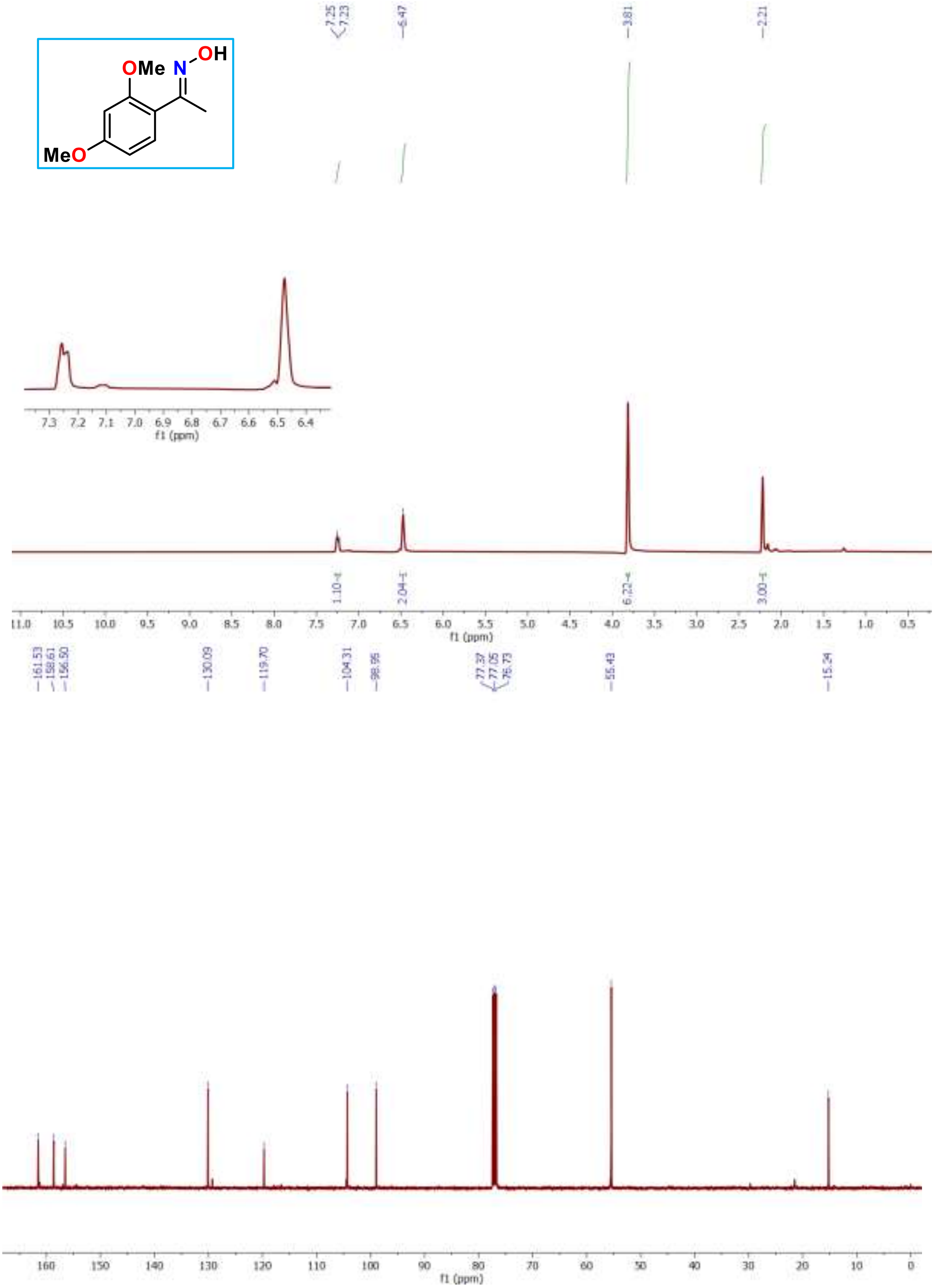

Figure S9: ^{1}H and ^{13}C NMR Spectrum of (E)-1-(2,4-dimethoxyphenyl)ethan-1-one oxime (**3h**).

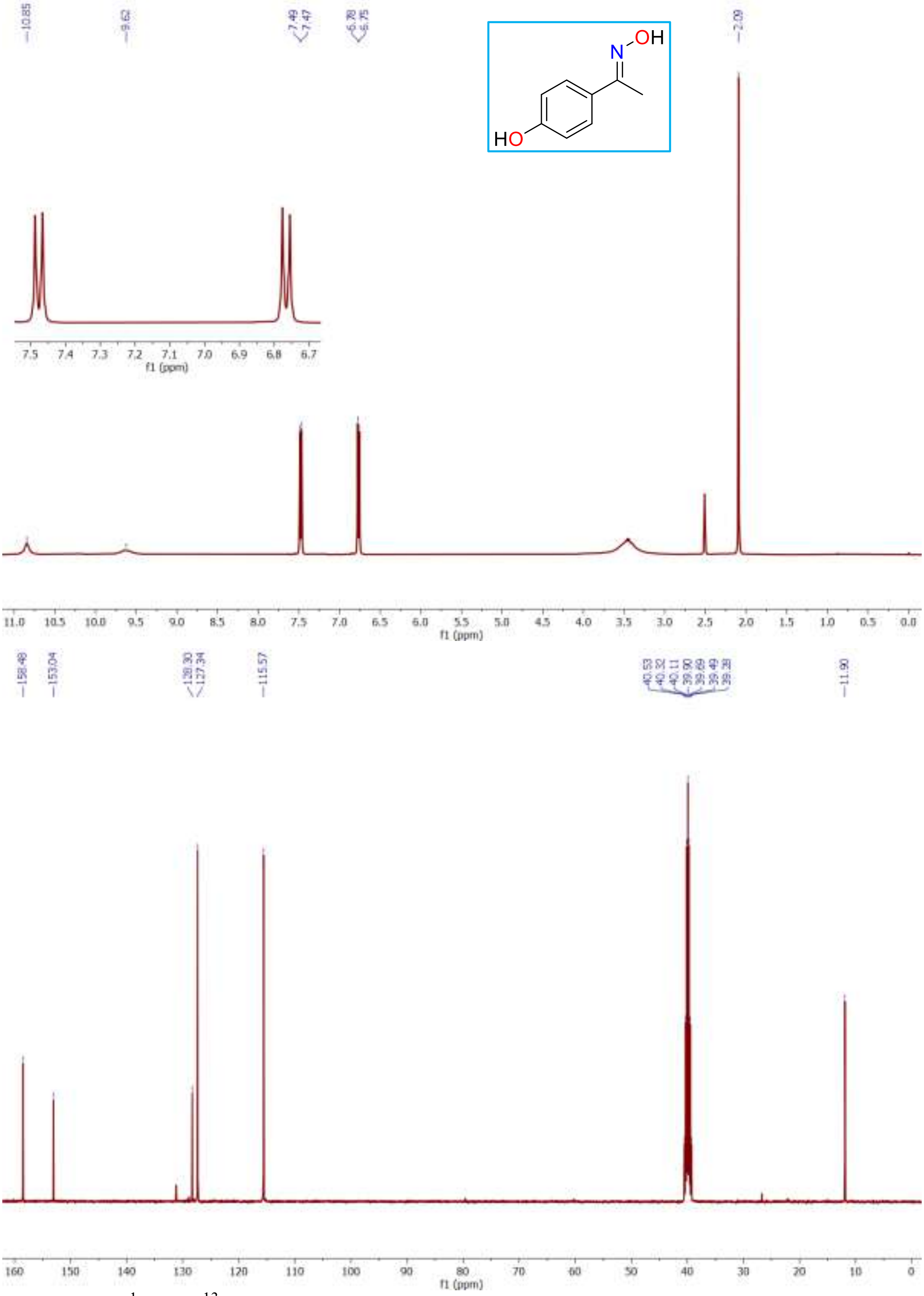

Figure S10: ^{1}H and ^{13}C NMR Spectrum of (E)-1-(4-hydroxyphenyl)ethan-1-one oxime (**3i**).

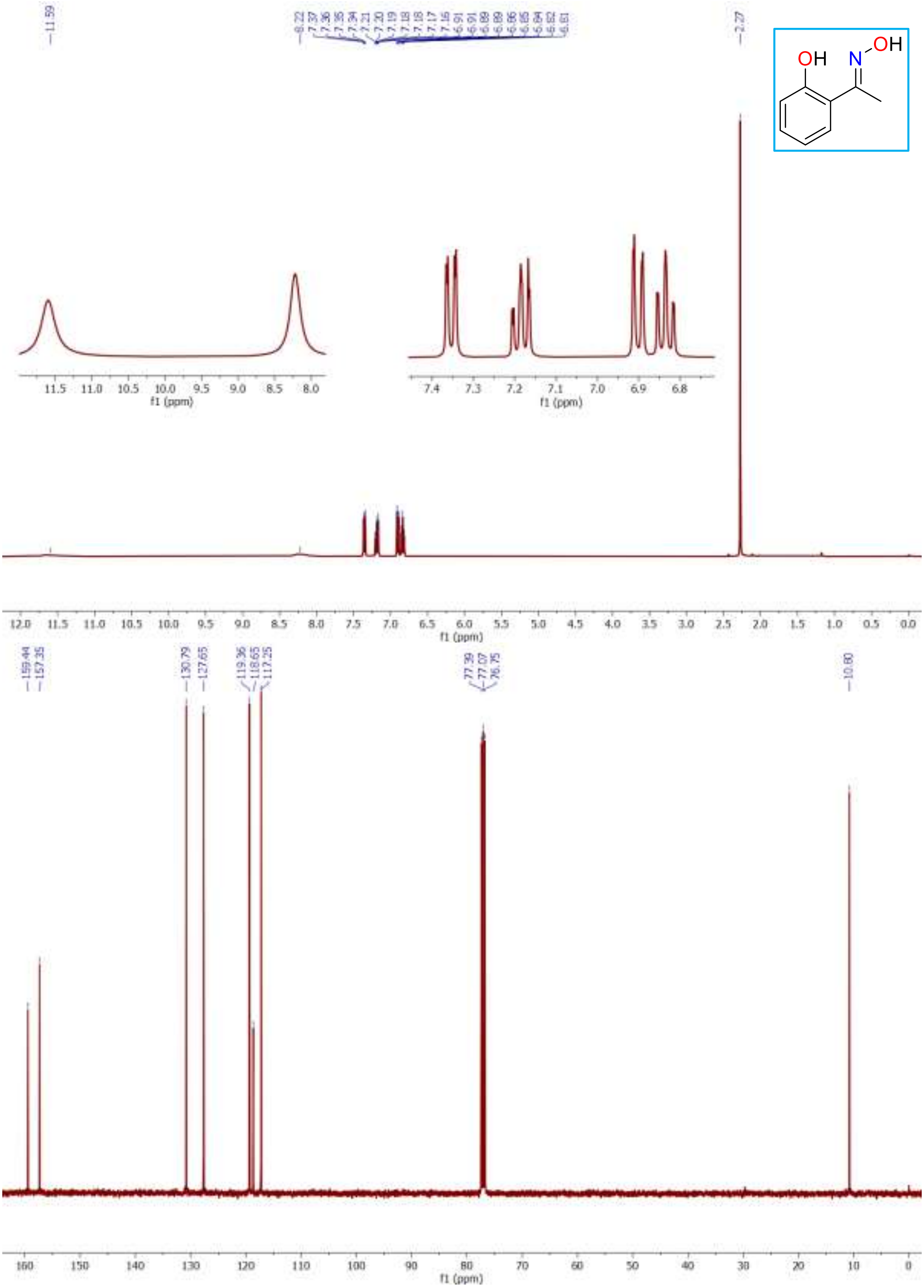

Figure S11: ^{1}H and ^{13}C NMR Spectrum of (E)-1-(2-hydroxyphenyl)ethan-1-one oxime (**3j**).

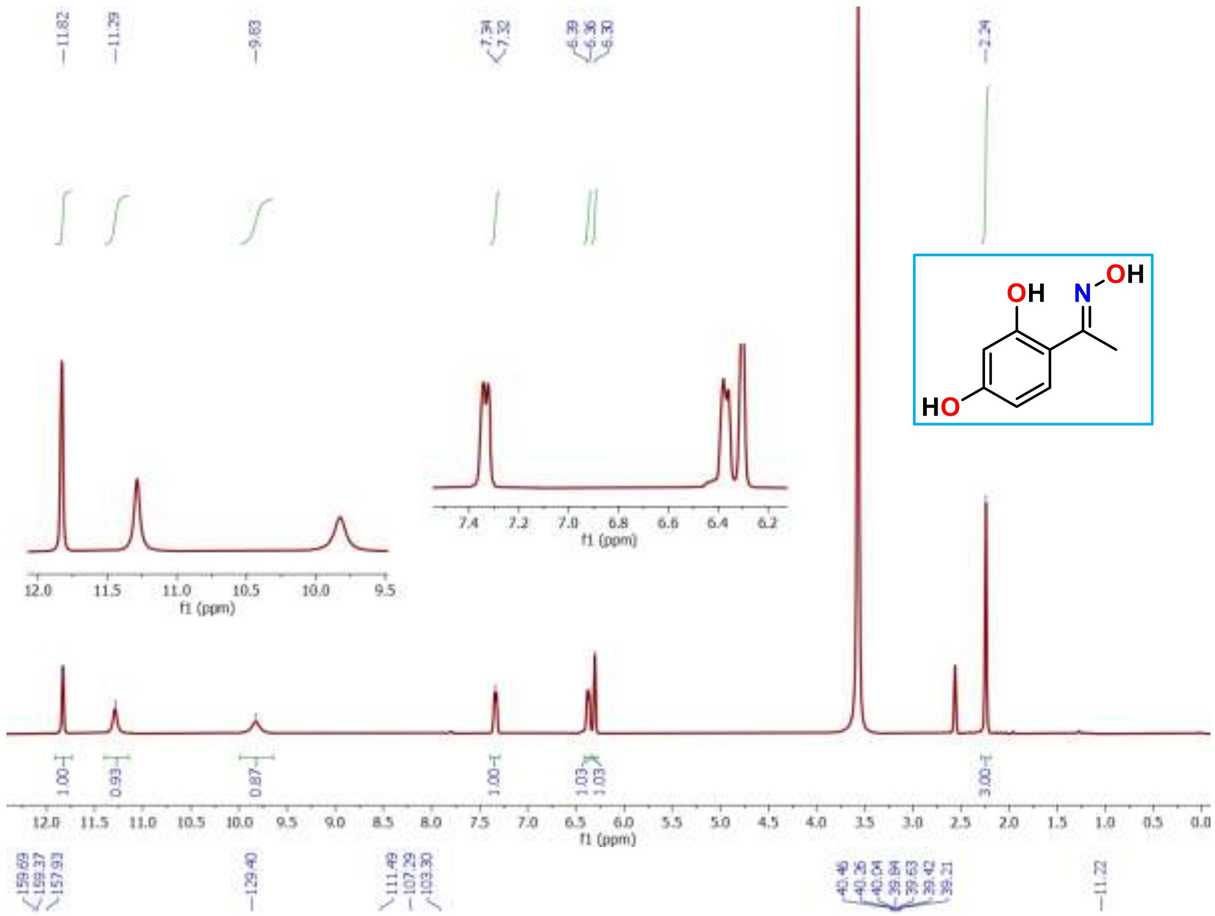
11.82
11.29
9.83
7.34
7.32
6.39
6.36
6.30
2.24
OH
N
OH
HO
12.0
11.5
11.0
10.5
10.0
9.5
f1 (ppm)
7.4
7.2
7.0
6.8
6.6
6.4
6.2
f1 (ppm)
1.00
0.93
0.87
1.00
1.03
1.03
3.00
12.0 11.5 11.0 10.5 10.0 9.5 9.0 8.5 8.0 7.5 7.0 6.5 6.0 5.5 5.0 4.5 4.0 3.5 3.0 2.5 2.0 1.5 1.0 0.5 0.0
f1 (ppm)

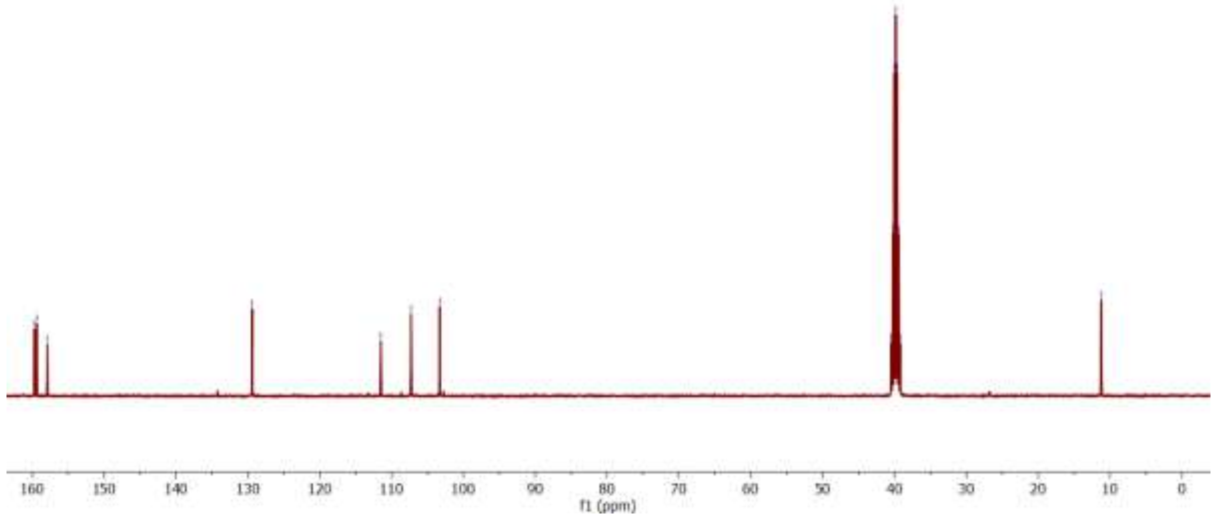
159.69
159.37
157.93
129.40
111.49
107.29
103.30
40.46
40.26
40.04
39.84
39.63
39.42
39.21
11.22
160 150 140 130 120 110 100 90 80 70 60 50 40 30 20 10 0
f1 (ppm)

Figure S12: ^{1}H and ^{13}C NMR Spectrum of (E)-1-(2,4-dihydroxyphenyl)ethan-1-one oxime (**3k**).

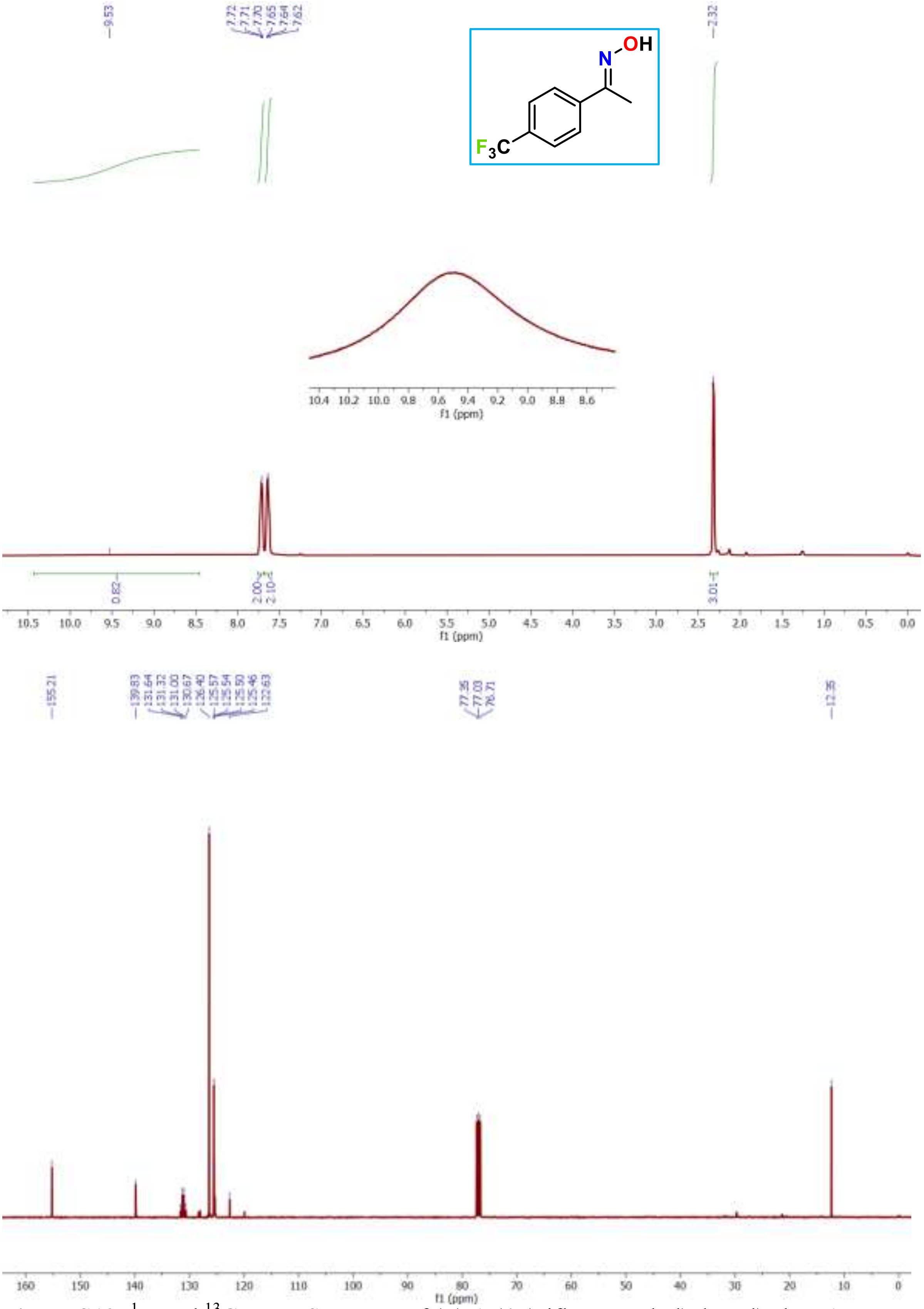

Figure S13: ^{1}H and ^{13}C NMR Spectrum of (E)-1-(4-(trifluoromethyl)phenyl)ethan-1-one oxime(**3l**).

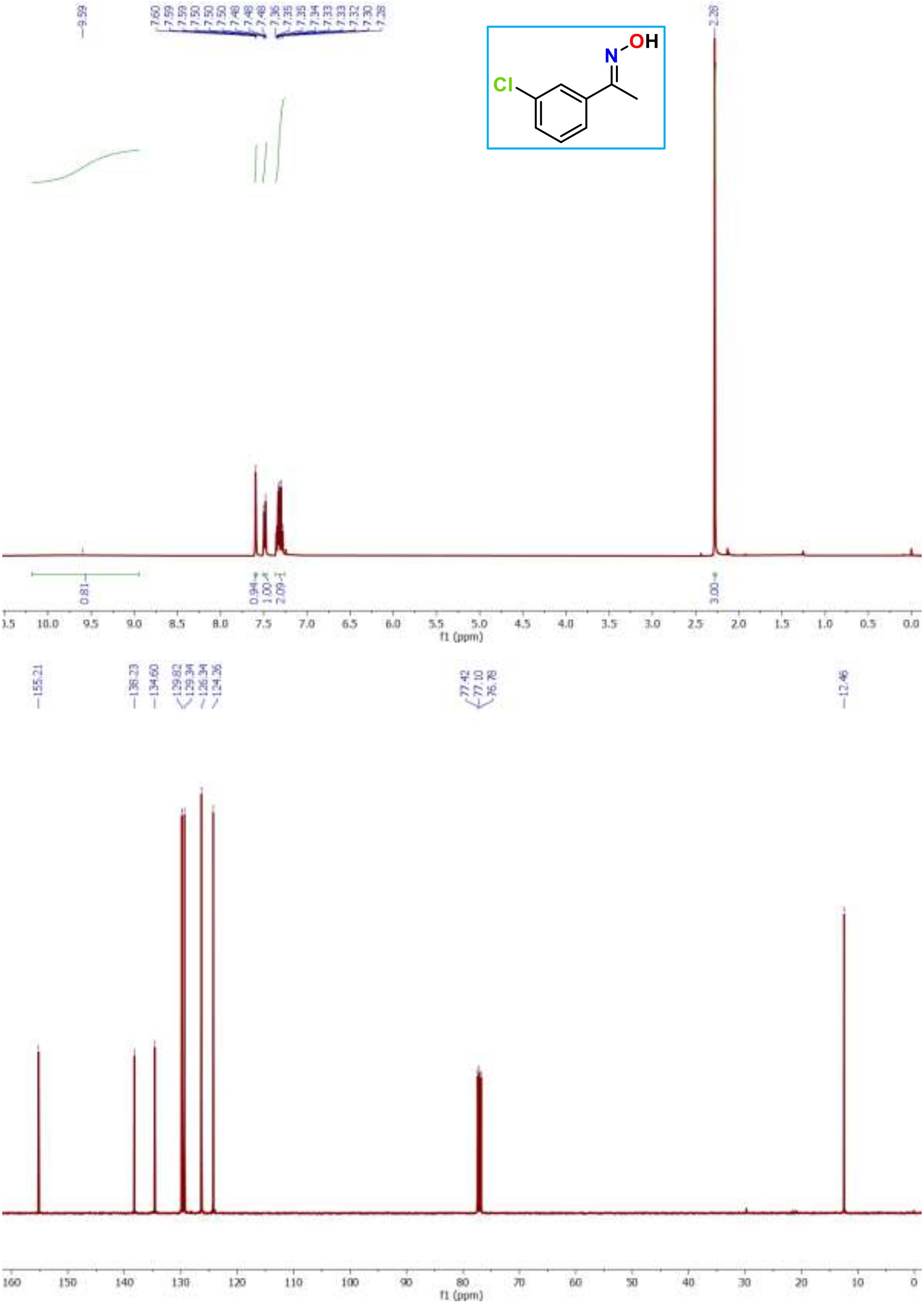

Figure S14: ^{1}H and ^{13}C NMR Spectrum of (E)-1-(3-chlorophenyl)ethan-1-one oxime (**3m**).

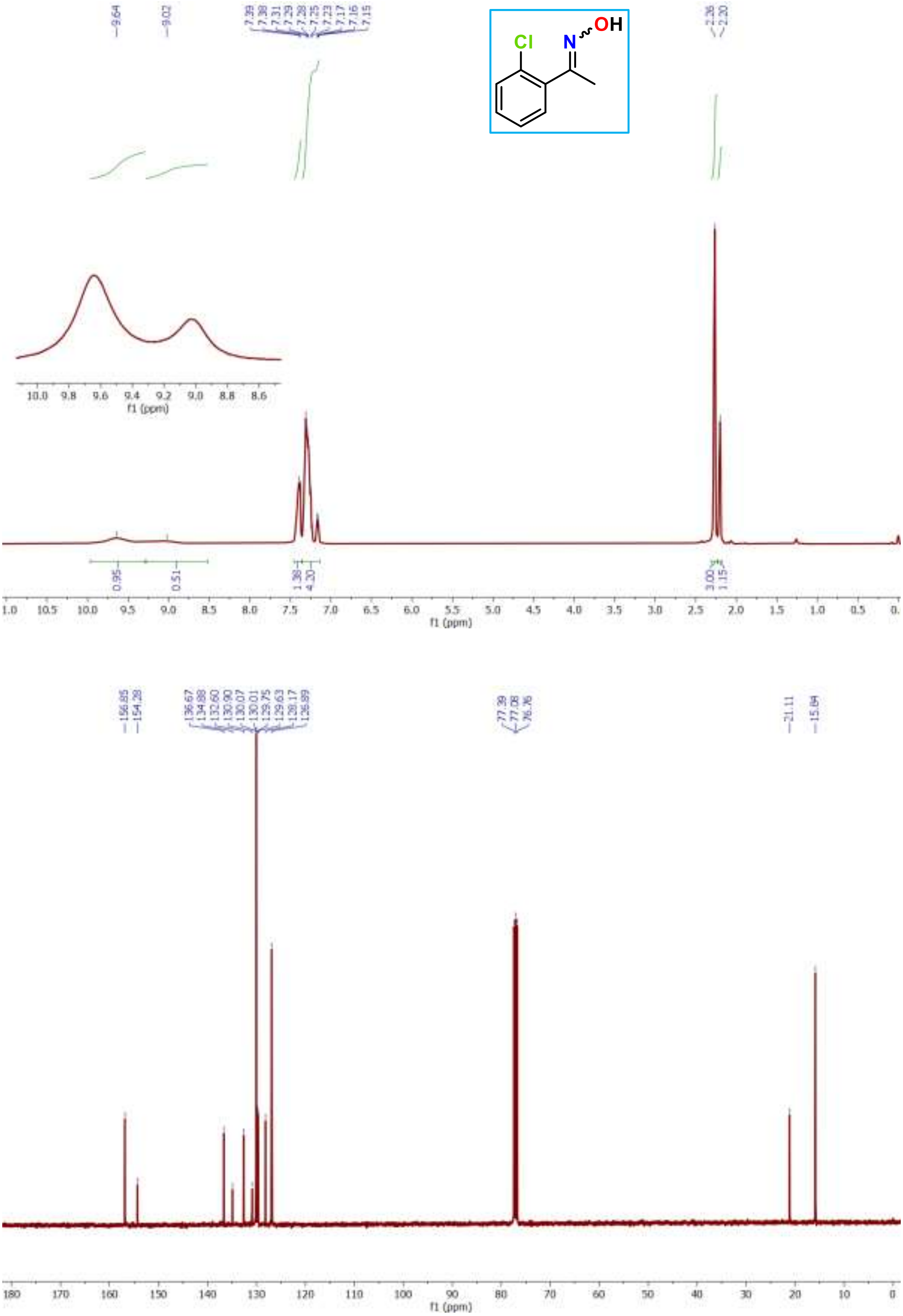

Figure S15: ^{1}H and ^{13}C NMR Spectrum of 1-(2-chlorophenyl)ethan-1-one oxime (**3n**).

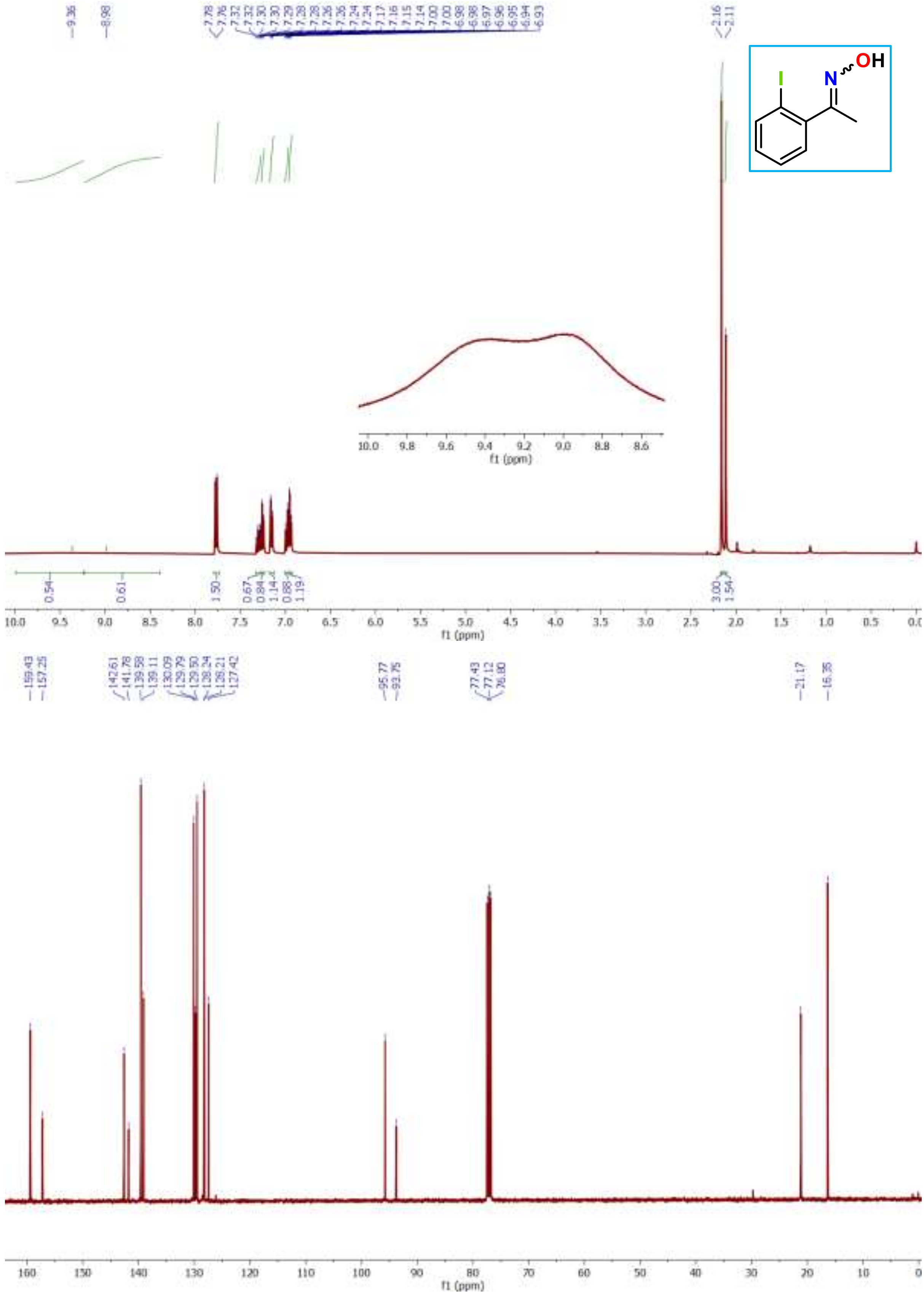

Figure S16: ^{1}H and ^{13}C NMR Spectrum of 1-(2-iodophenyl)ethan-1-one oxime (**3o**).

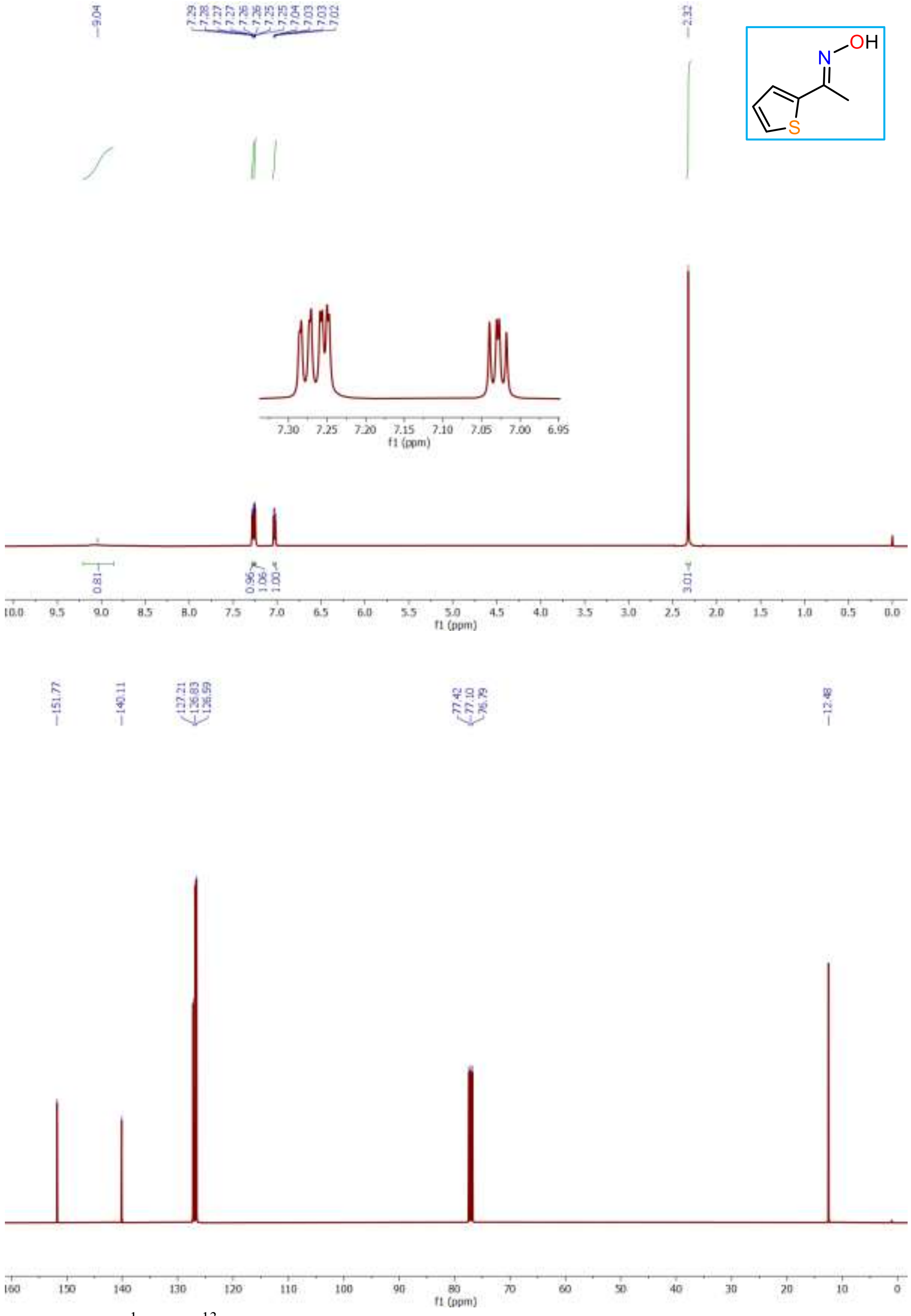

Figure S17: ^{1}H and ^{13}C NMR Spectrum of (E)-1-(thiophen-2-yl)ethan-1-one oxime (**3p'**).

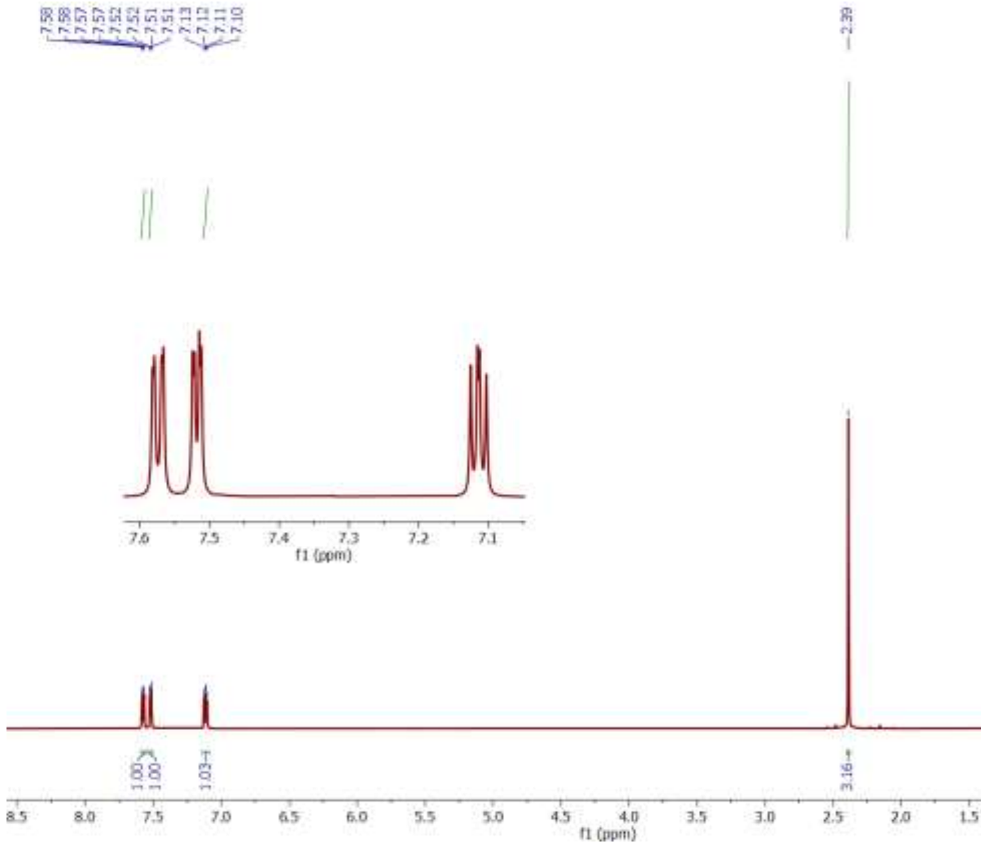

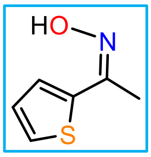

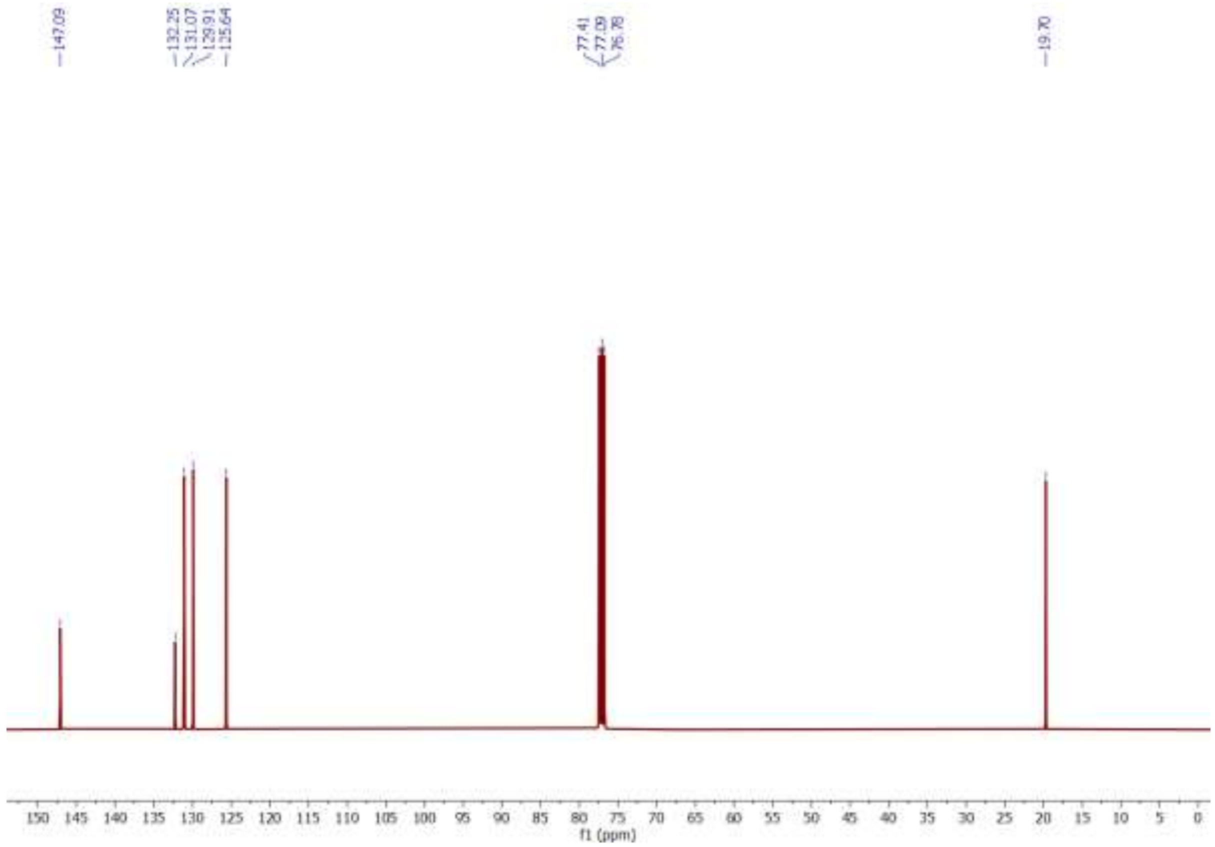

Figure S18: ^{1}H and ^{13}C NMR Spectrum of (Z)-1-(thiophen-2-yl)ethan-1-one oxime (**3p”**).

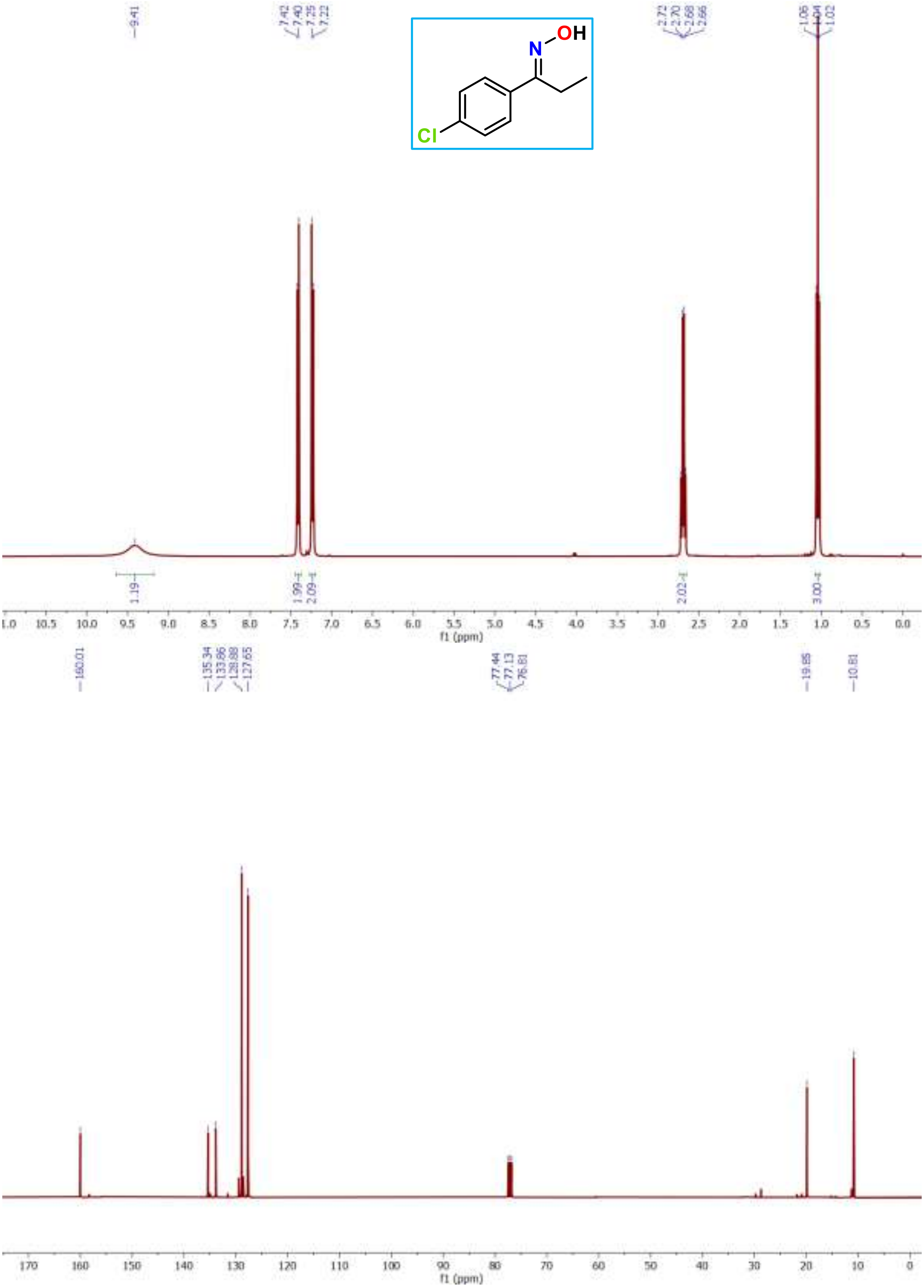

Figure S19: ^{1}H and ^{13}C NMR Spectrum of (E)-1-(4-chlorophenyl)propan-1-one oxime (**3q**).

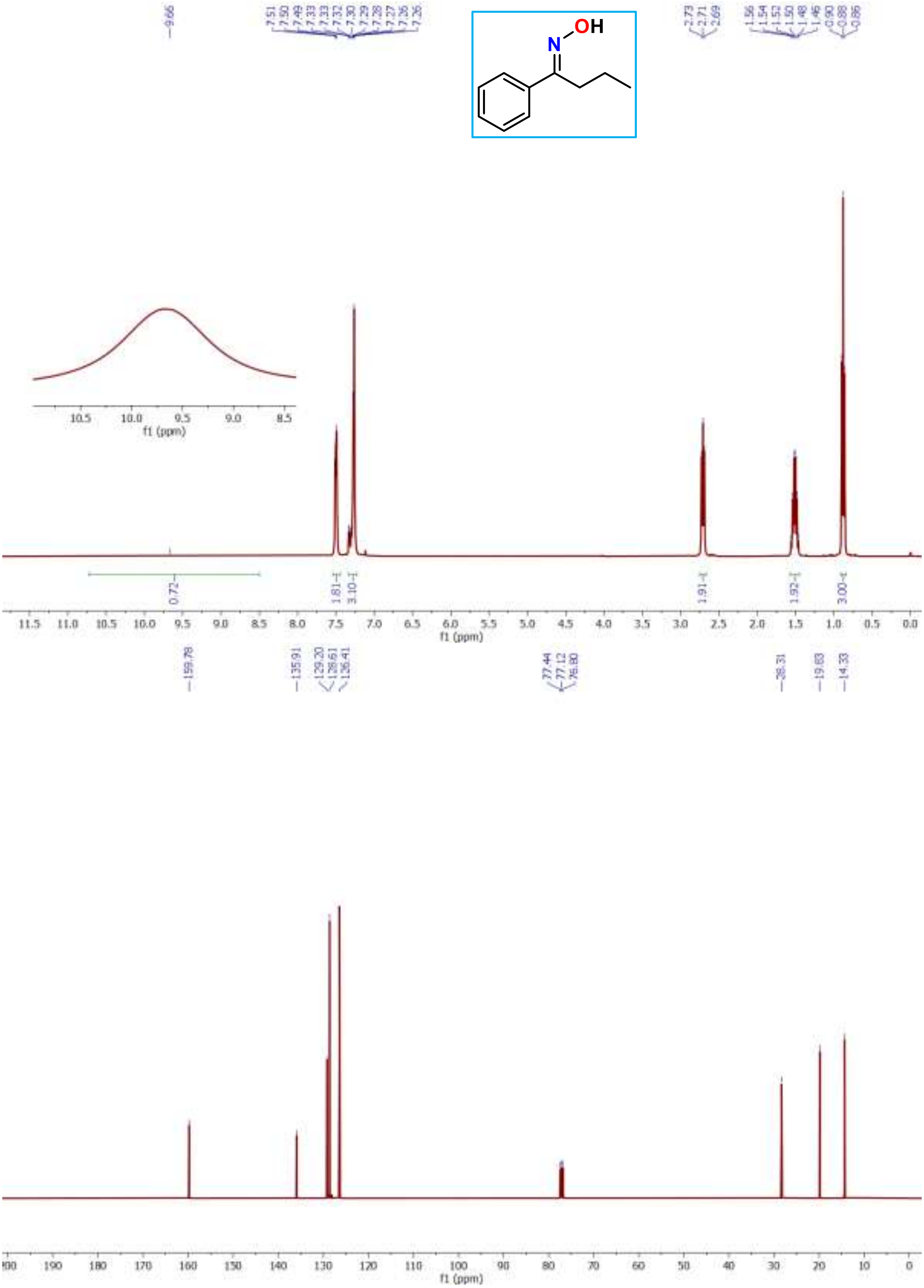

Figure S20: ^{1}H and ^{13}C NMR Spectrum of (E)-1-Phenylbutan-1-one oxime (**3r**).

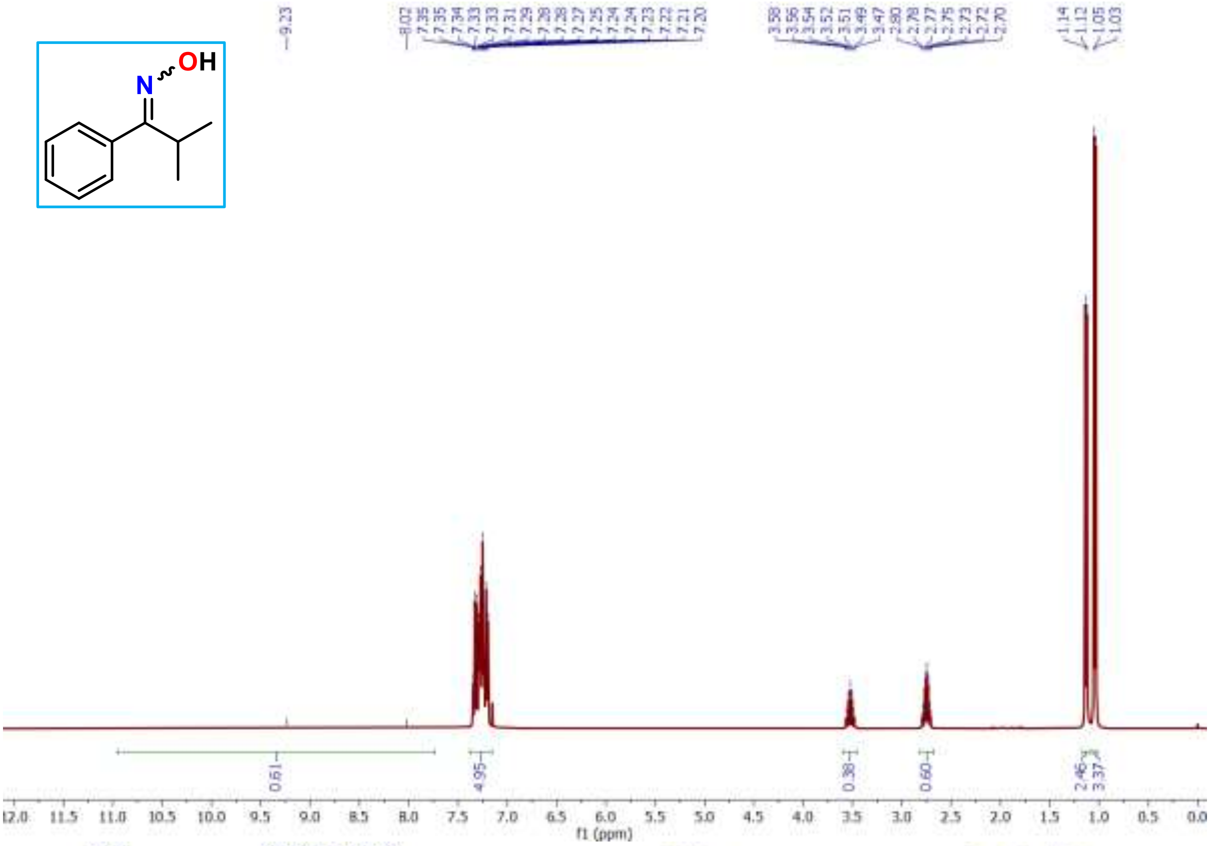

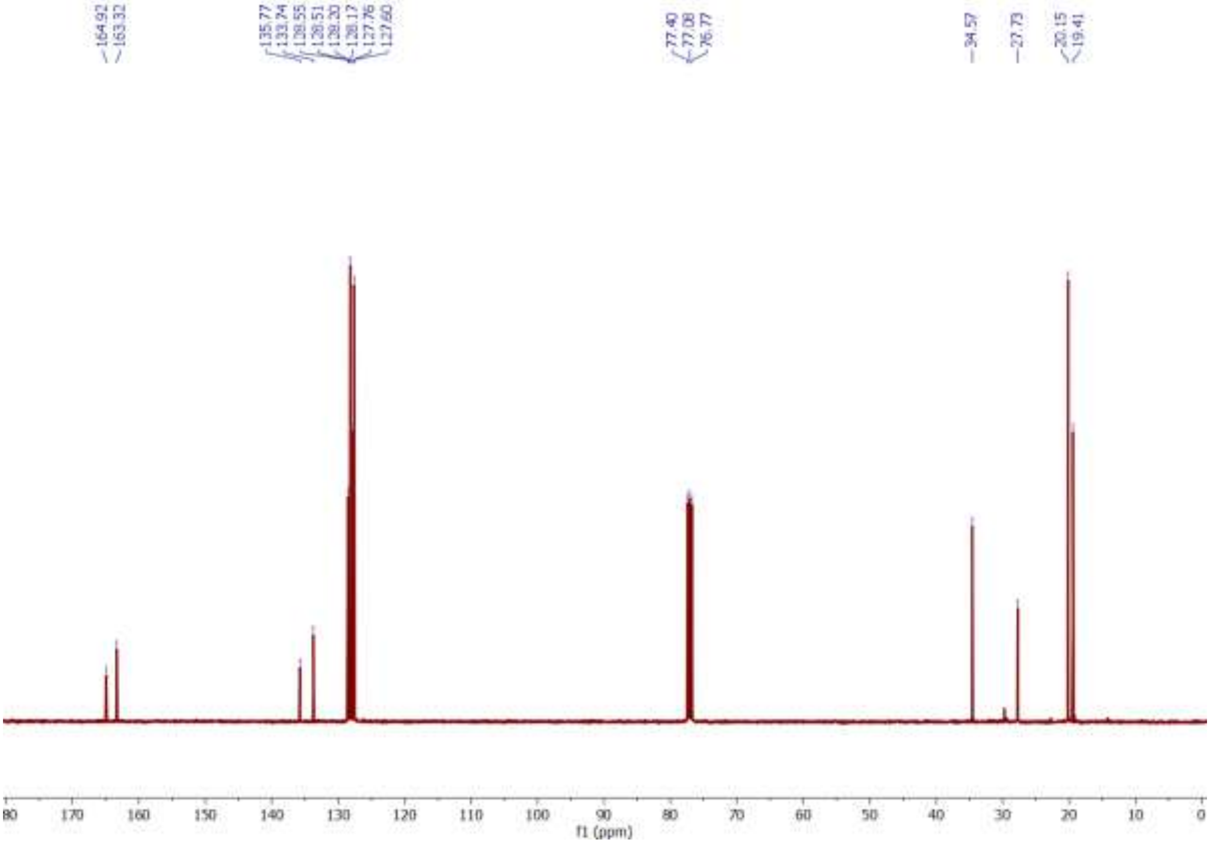

Figure S21: ^{1}H and ^{13}C NMR Spectrum of 2-Methyl-1-phenylpropan-1-one oxime (**3s**).

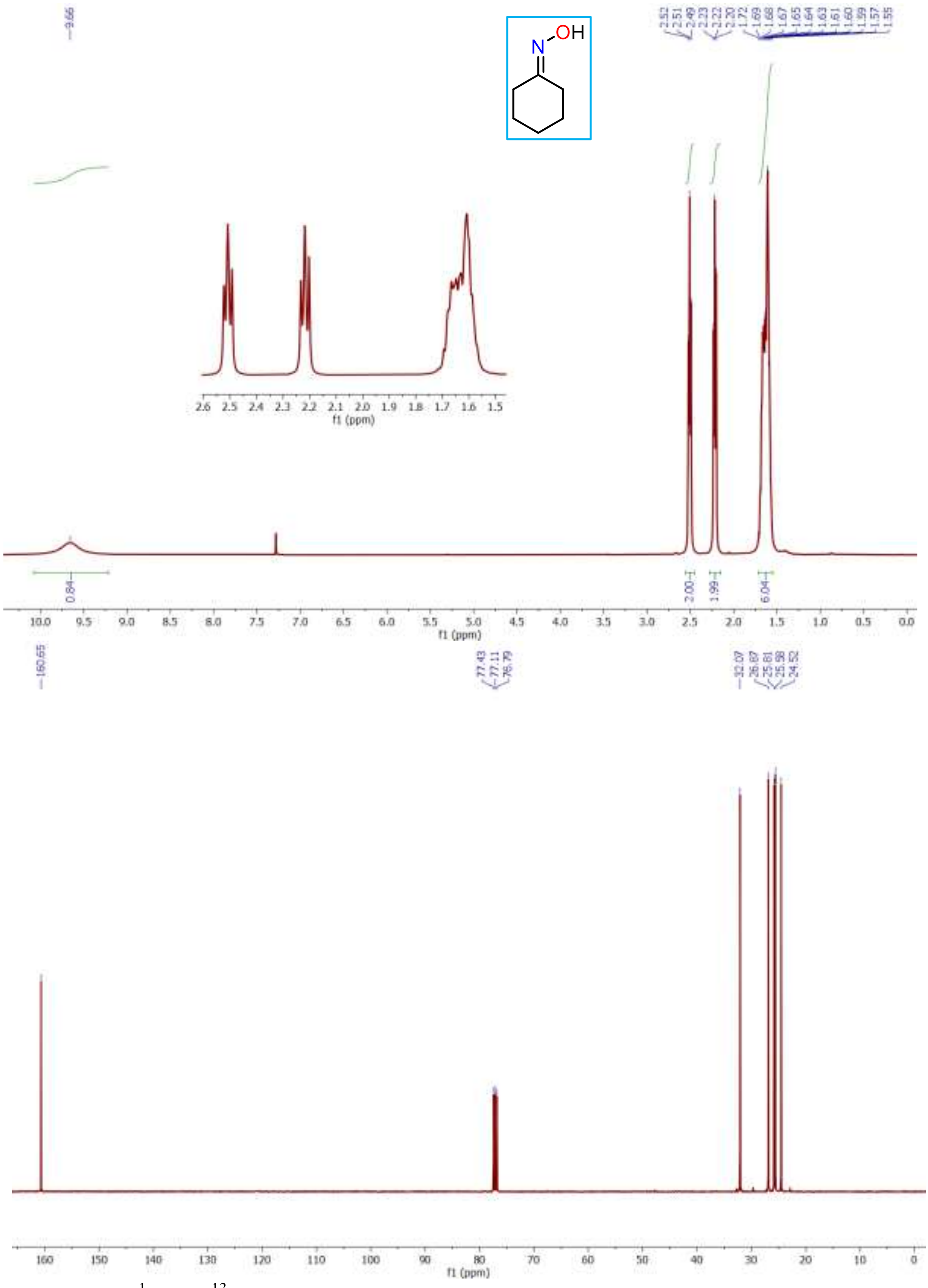

Figure S22: ^{1}H and ^{13}C NMR Spectrum of cyclohexanone oxime (**3t**).

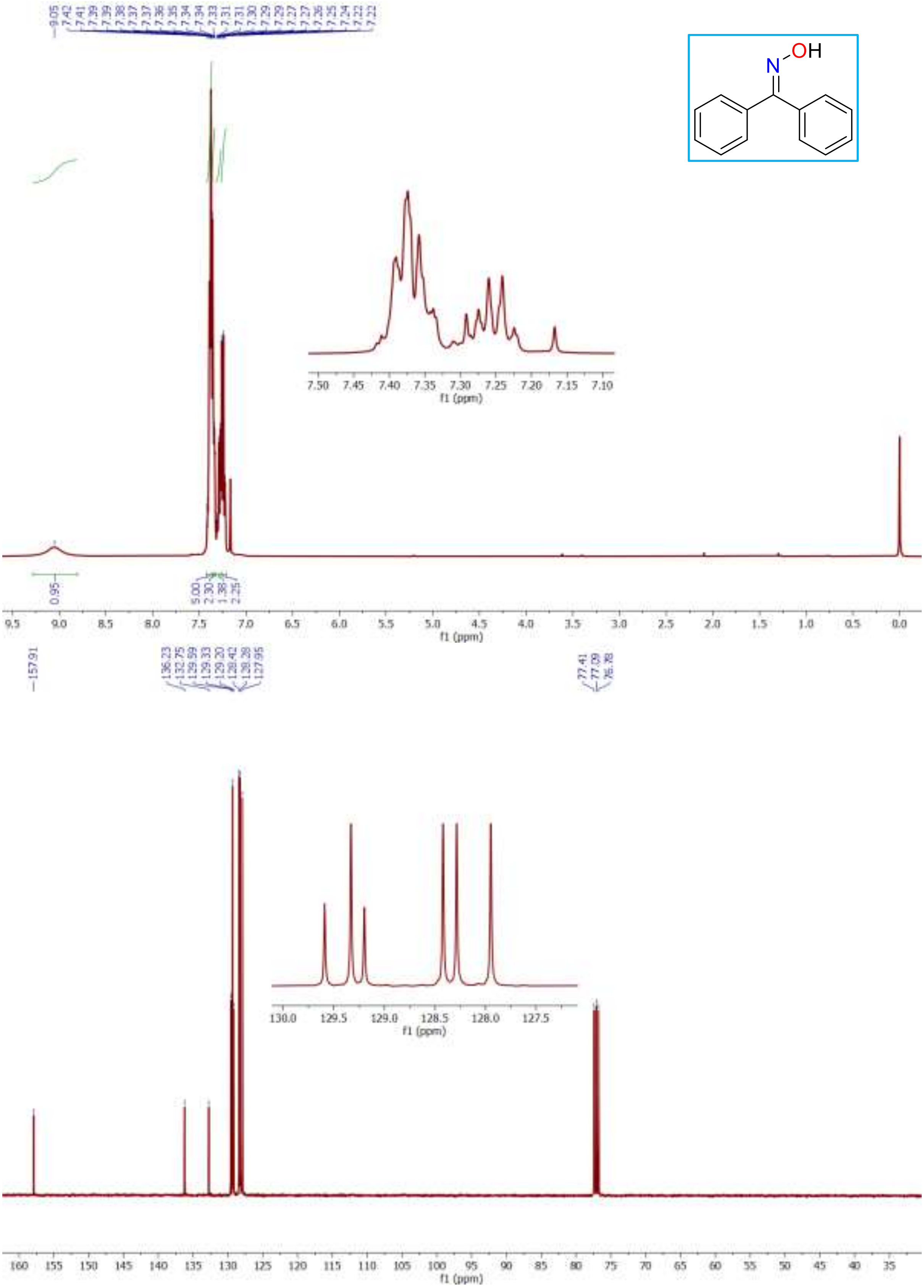

Figure S23: ^{1}H and ^{13}C NMR Spectrum of diphenylmethanone oxime (**3u**).

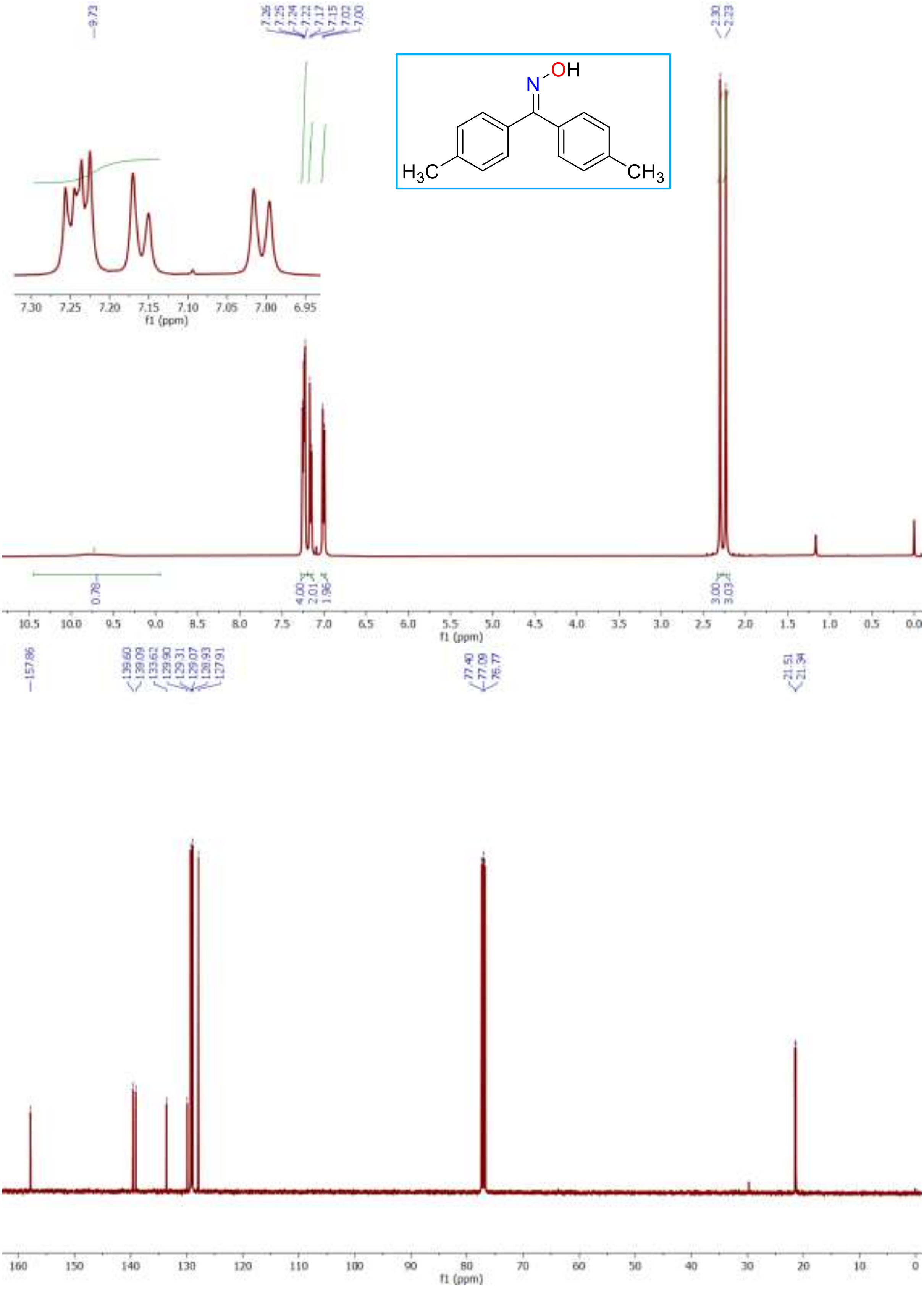

Figure S24: ^{1}H and ^{13}C NMR Spectrum of di-p-tolylmethanone oxime (**3v**).

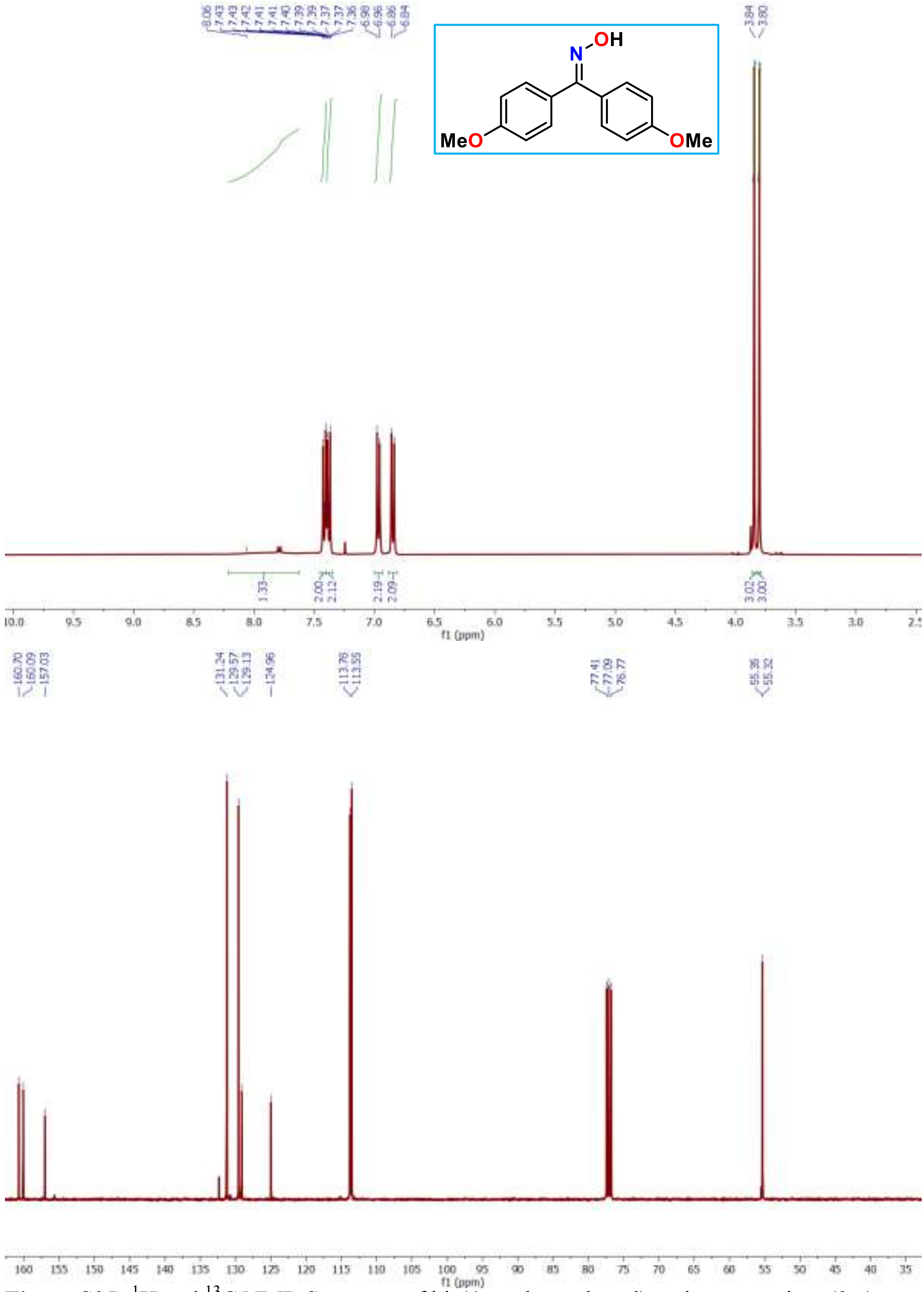

Figure S25: ^{1}H and ^{13}C NMR Spectrum of bis(4-methoxyphenyl)methanone oxime (**3w**).

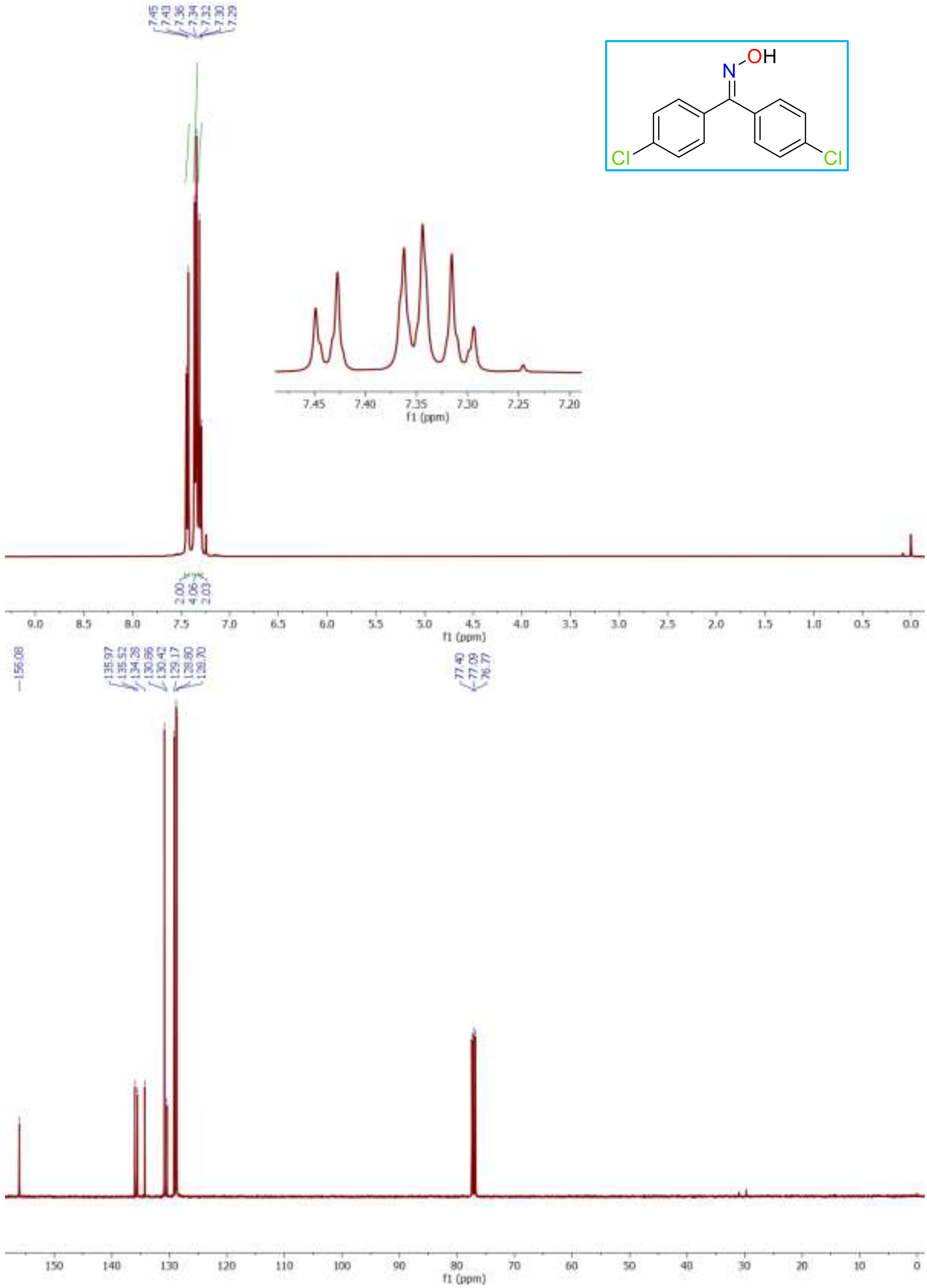

Figure S26: ^{1}H and ^{13}C NMR Spectrum of bis(4-chlorophenyl)methanone oxime (**3x**).

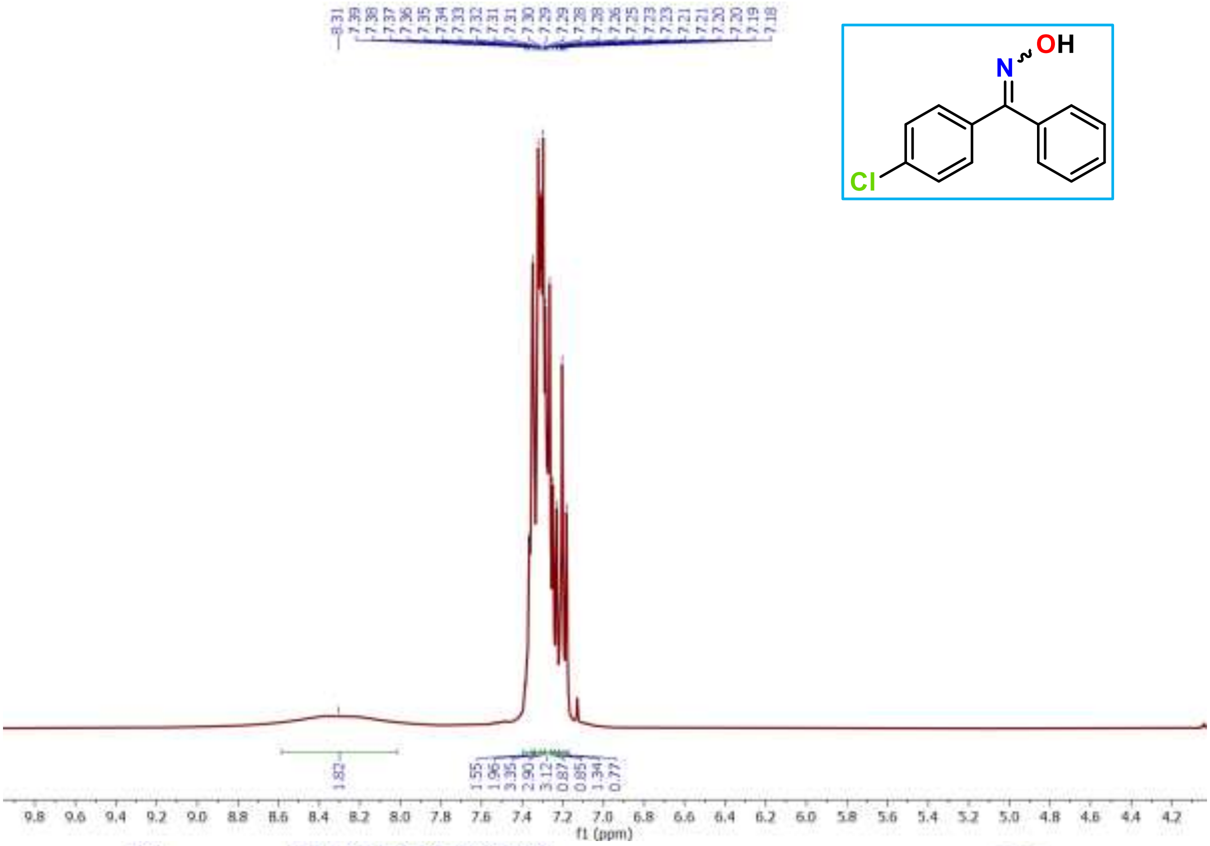

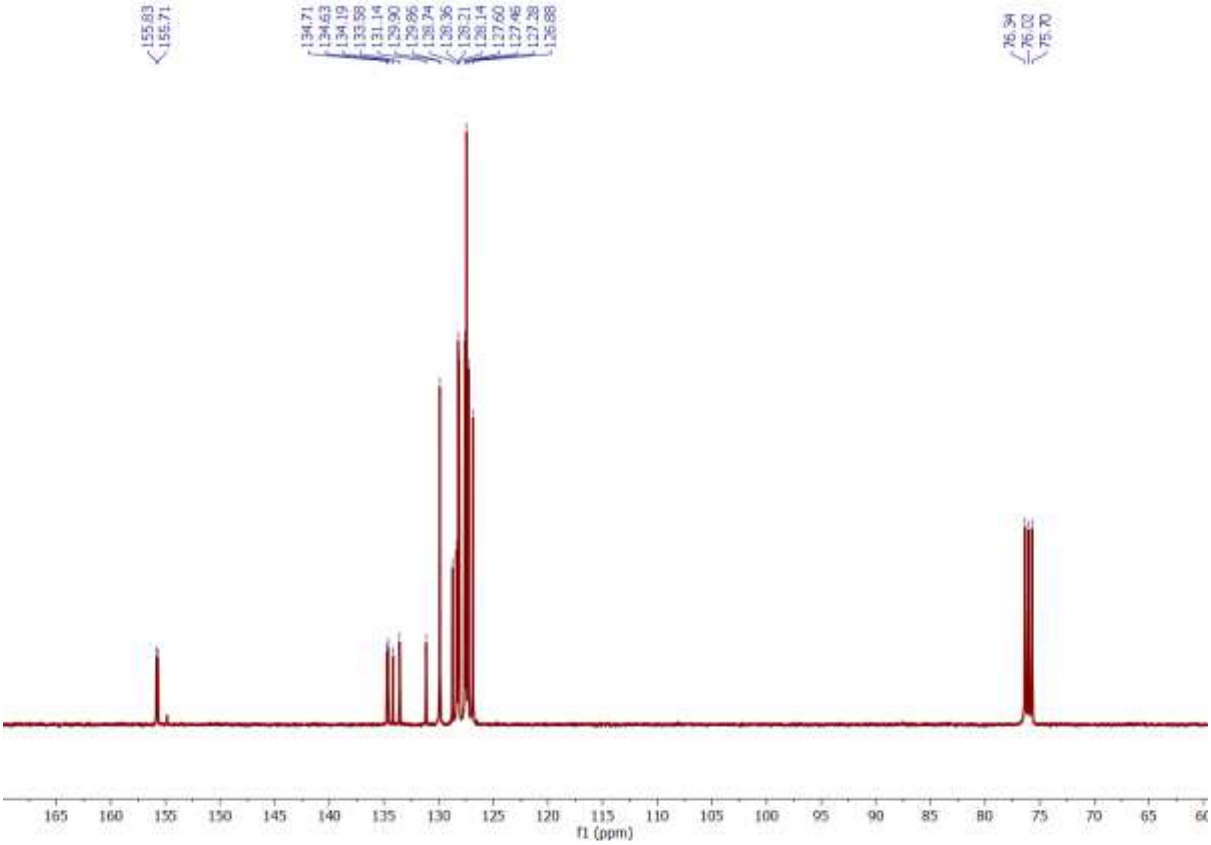

Figure S27: ^{1}H and ^{13}C NMR Spectrum of (4-chlorophenyl)(phenyl)methanone oxime (**3y**).

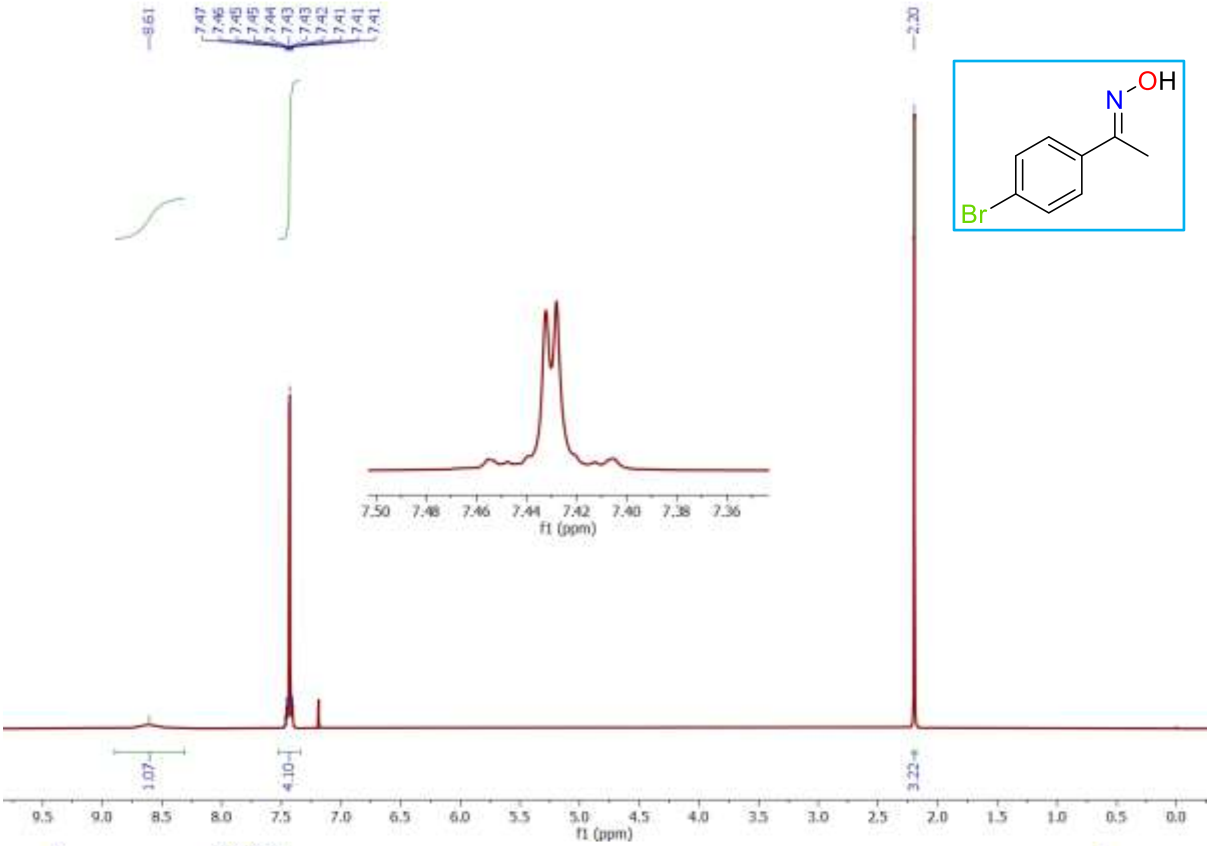

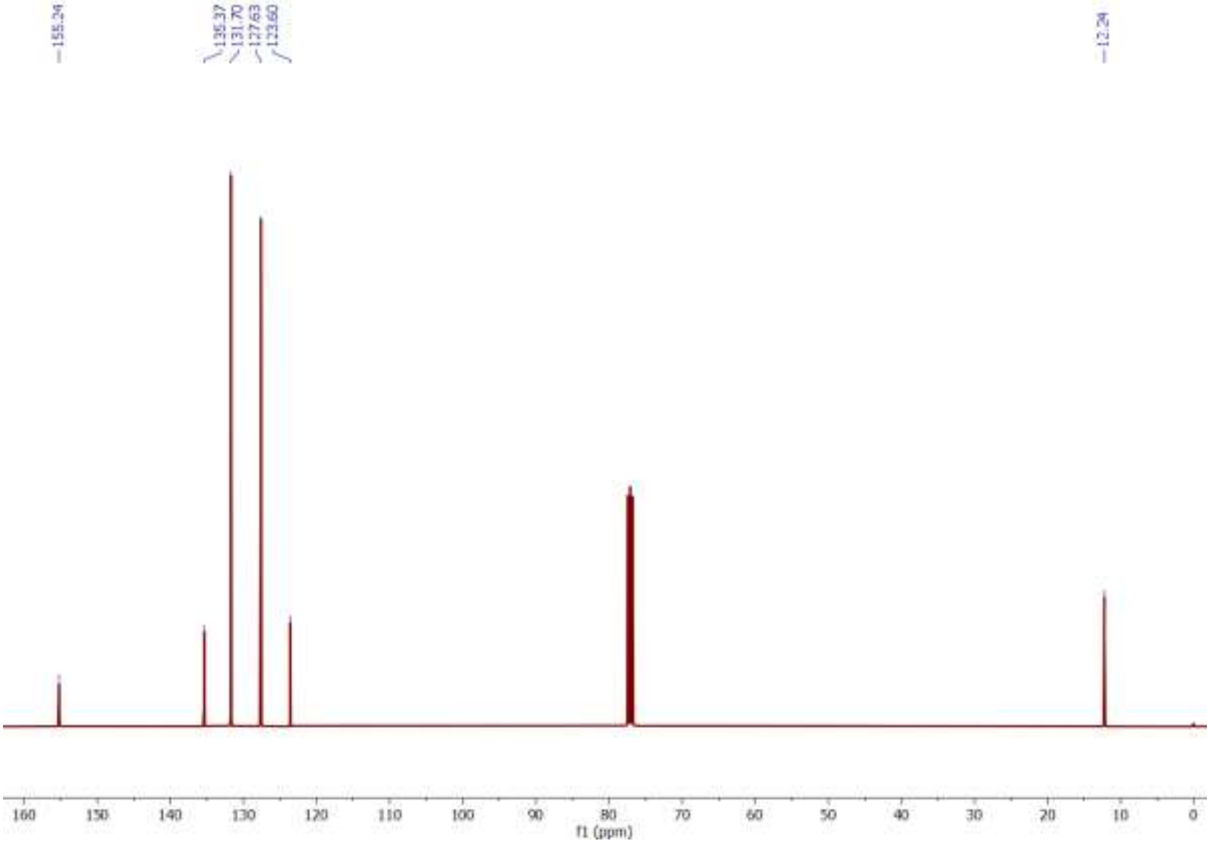

Figure S28: ^{1}H and ^{13}C NMR Spectrum of (E)-1-(4-bromophenyl)ethan-1-one oxime (**3z**).

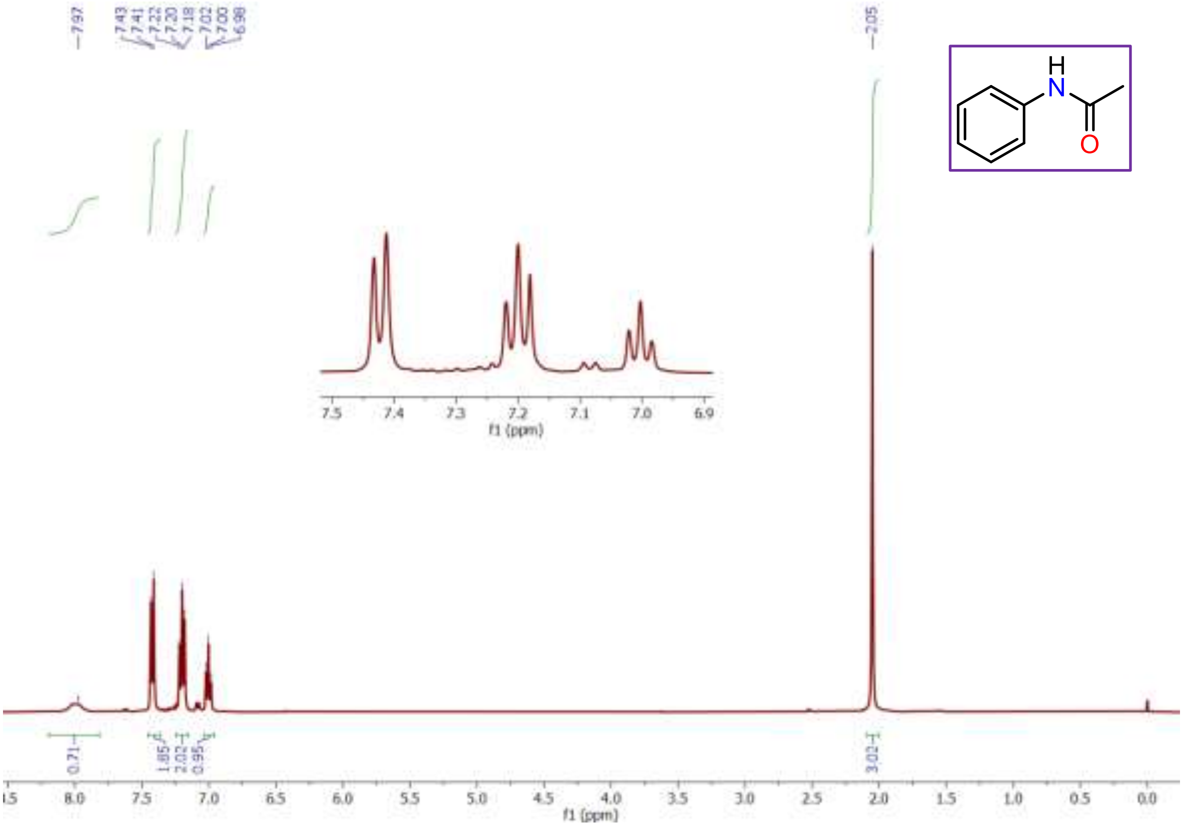

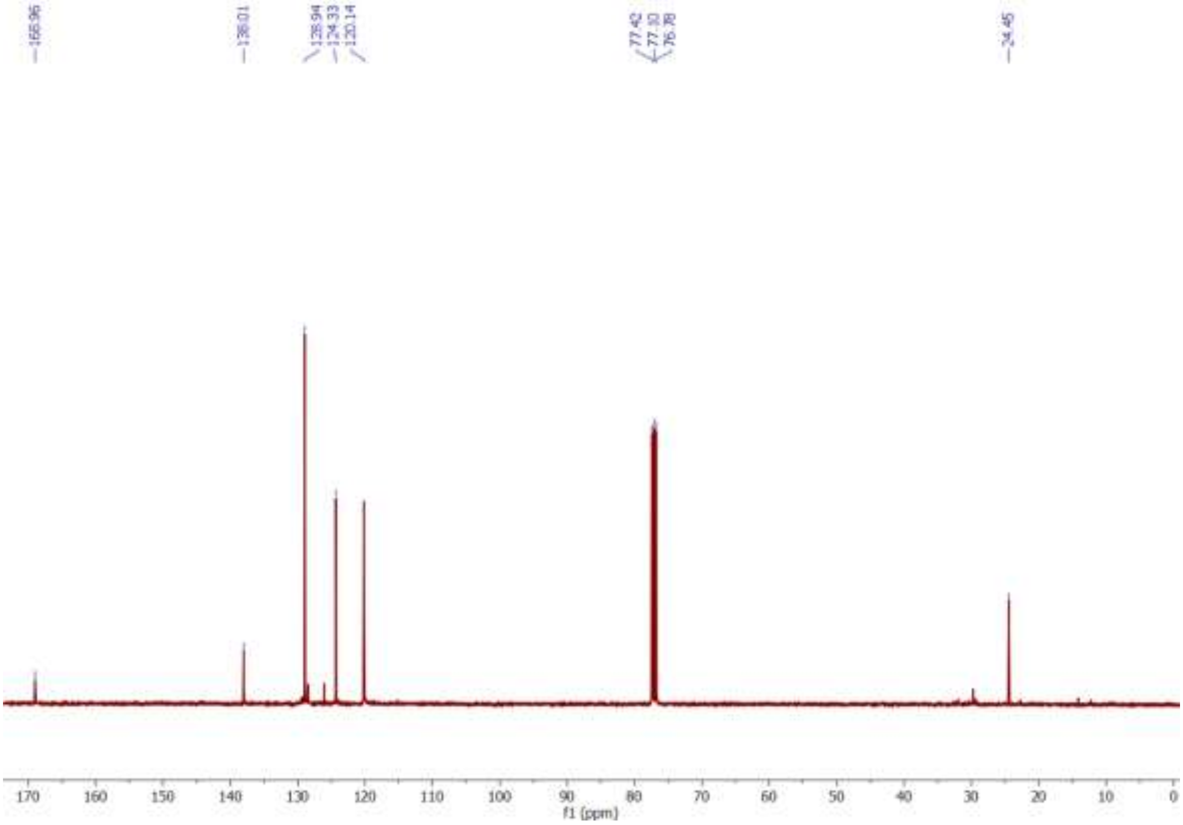

Figure S29: ^{1}H and ^{13}C NMR Spectrum of N-Phenylacetamide (**4a**).

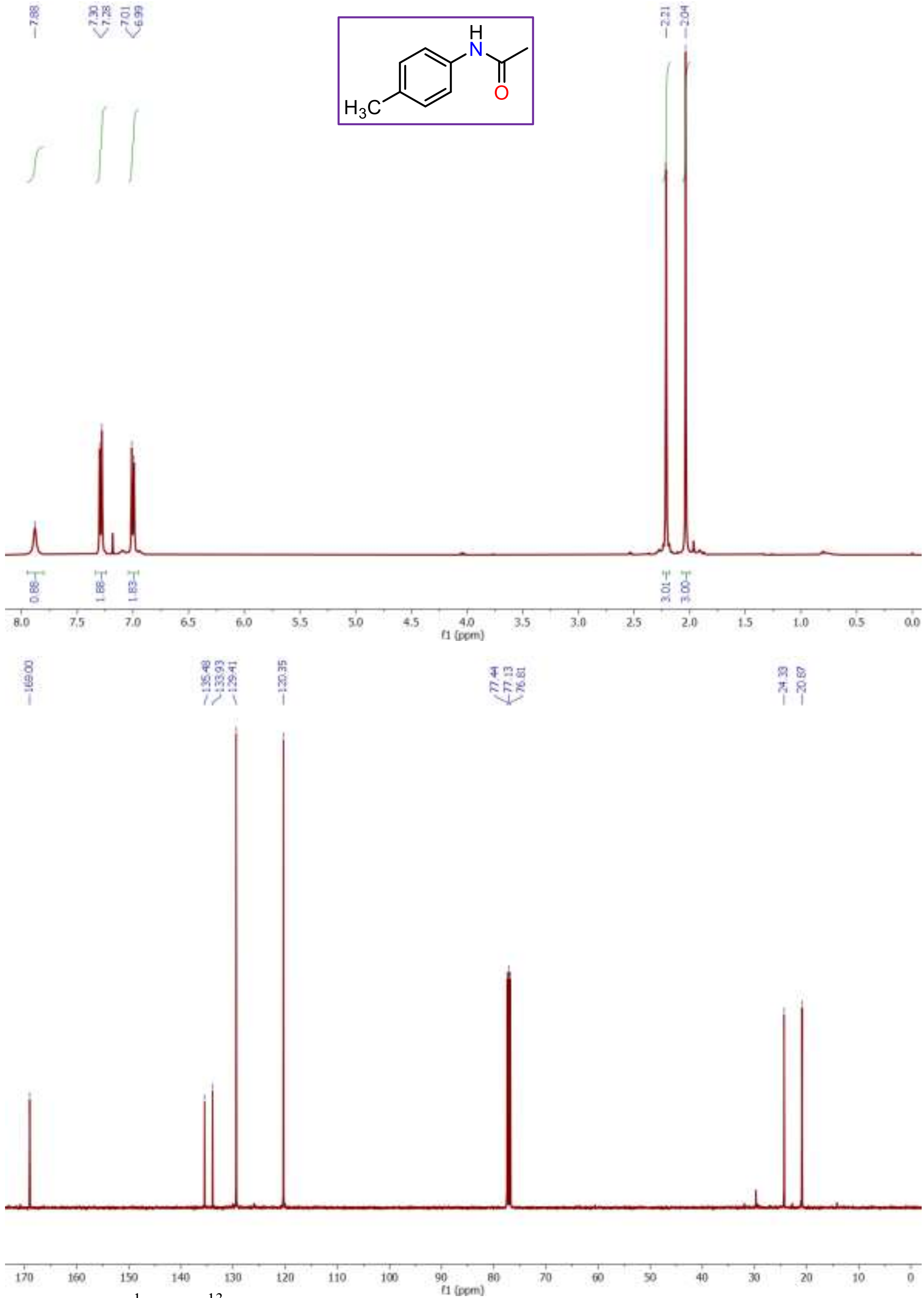

Figure S30: ^{1}H and ^{13}C NMR Spectrum of N-p-Tolylacetamide (**4b**).

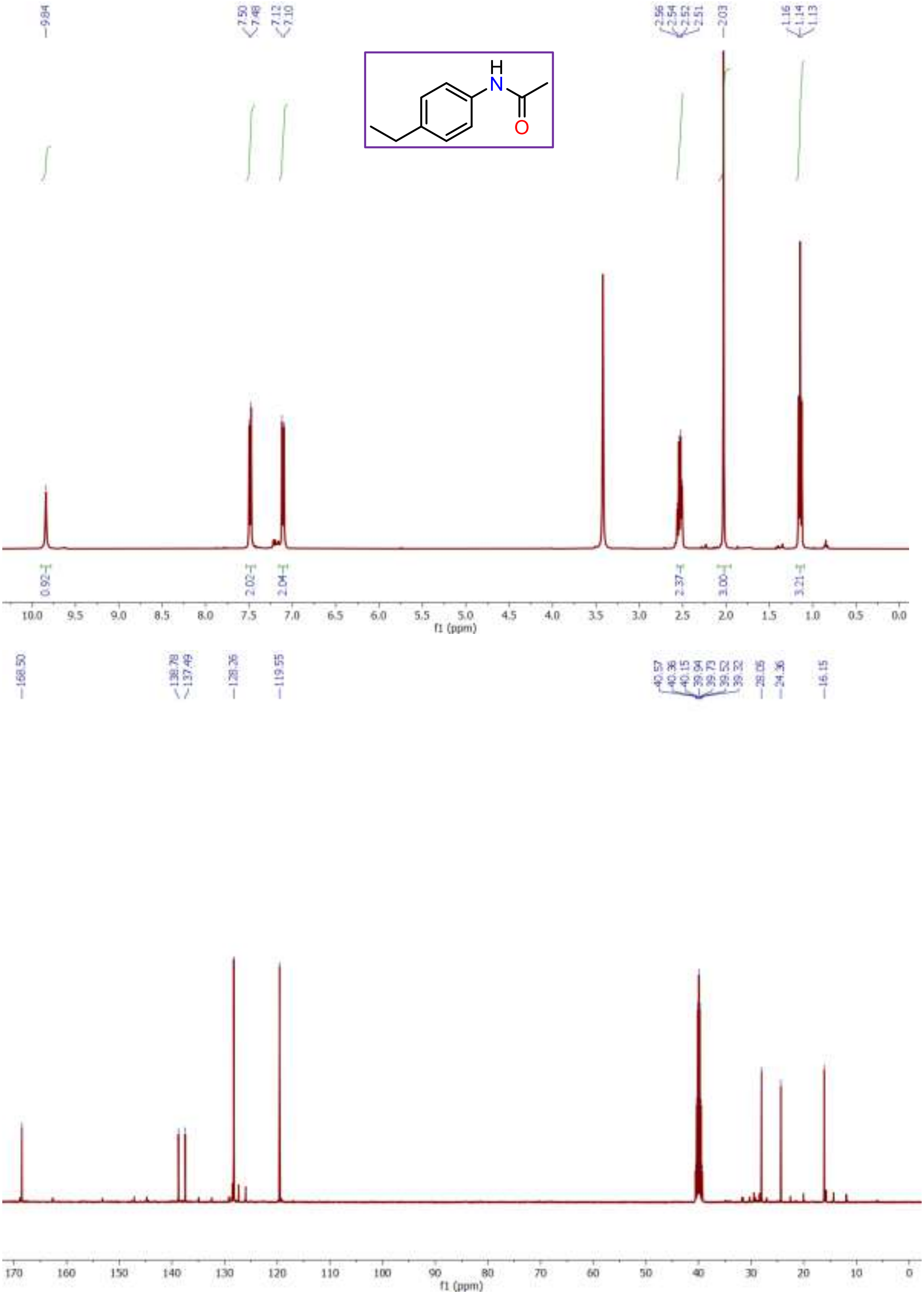

Figure S31: ^{1}H and ^{13}C NMR Spectrum of N-(4-Ethylphenyl)acetamide (**4c**).

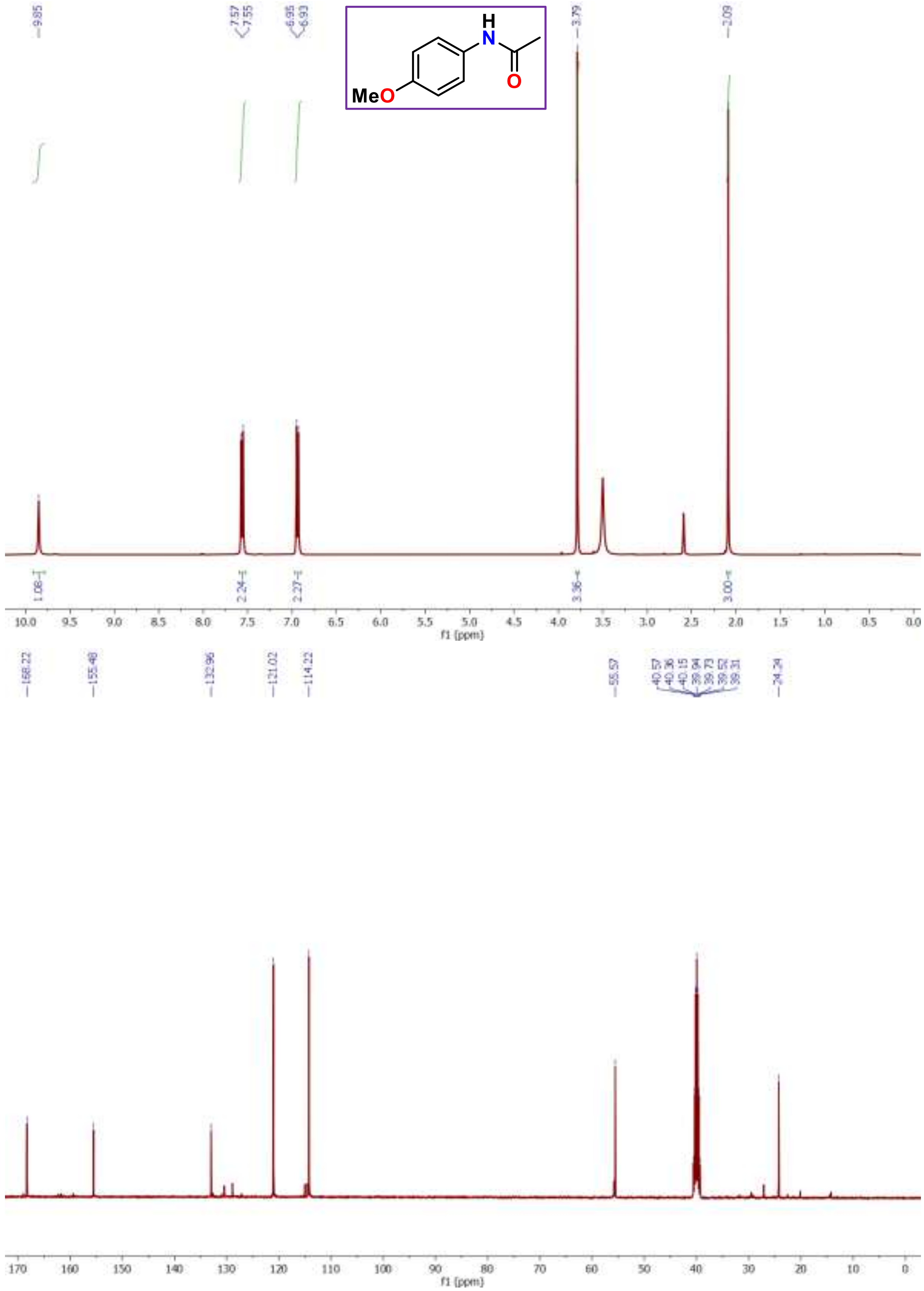

Figure S32: ^{1}H and ^{13}C NMR Spectrum of (N-(4-methoxyphenyl)acetamide) (**4d**).

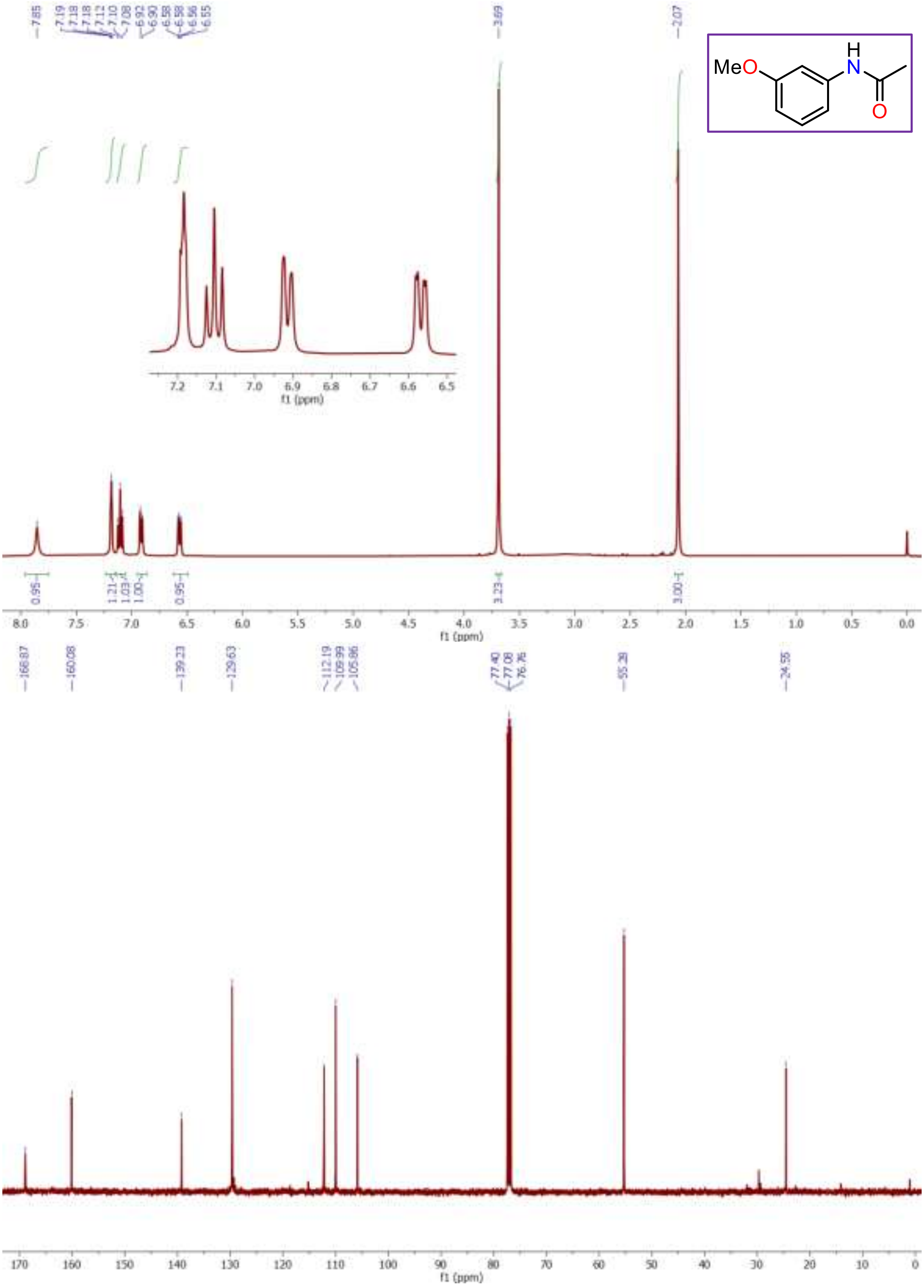

Figure S33: ^{1}H and ^{13}C NMR Spectrum of *N*-(3-Methoxyphenyl)acetamide (**4e**).

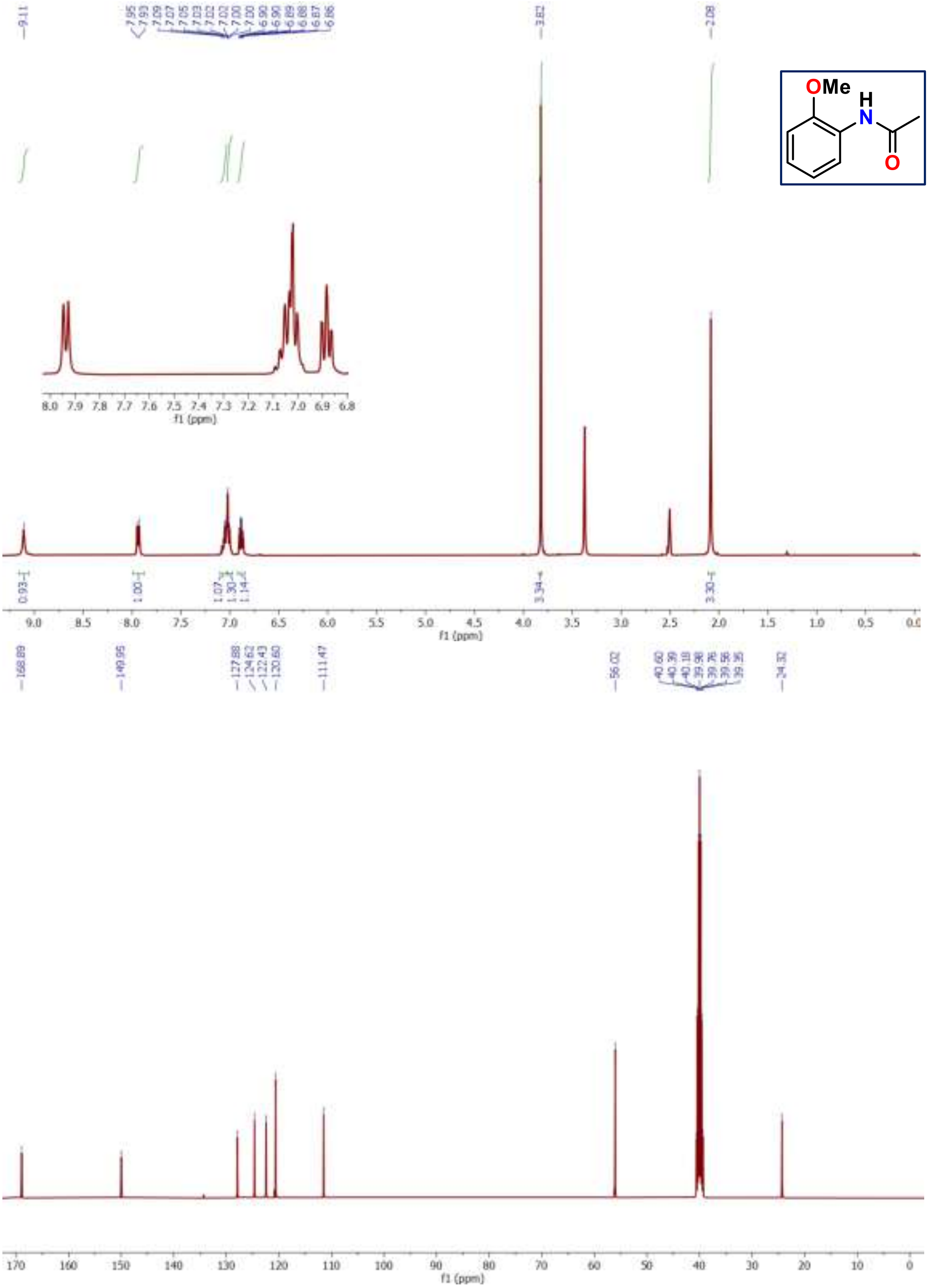

Figure S34: ^{1}H and ^{13}C NMR Spectrum of N-(2-methoxyphenyl)acetamide (**4f**).

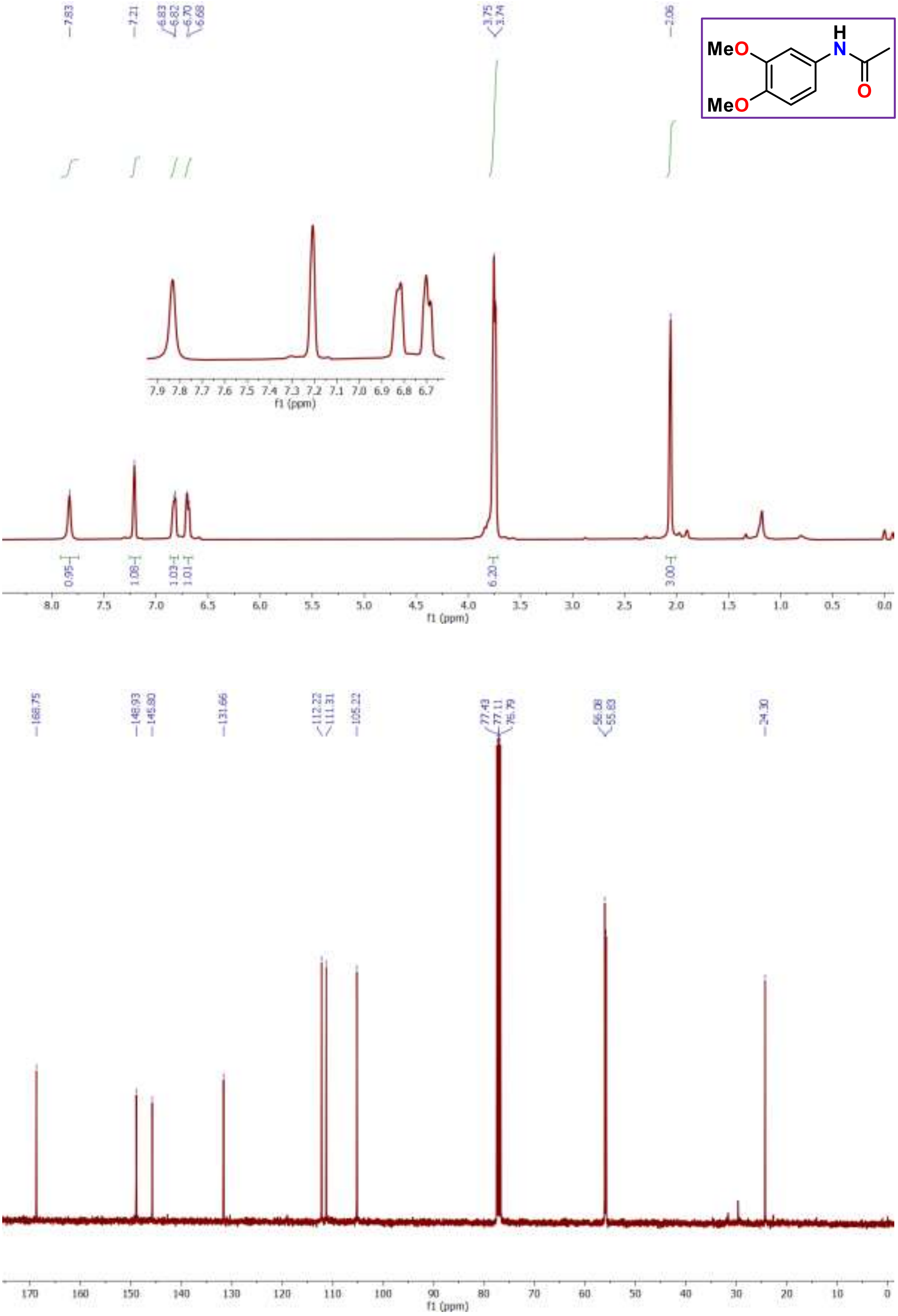

Figure S35: ^{1}H and ^{13}C NMR Spectrum of N-(3,4-dimethoxyphenyl)acetamide (**4g**).

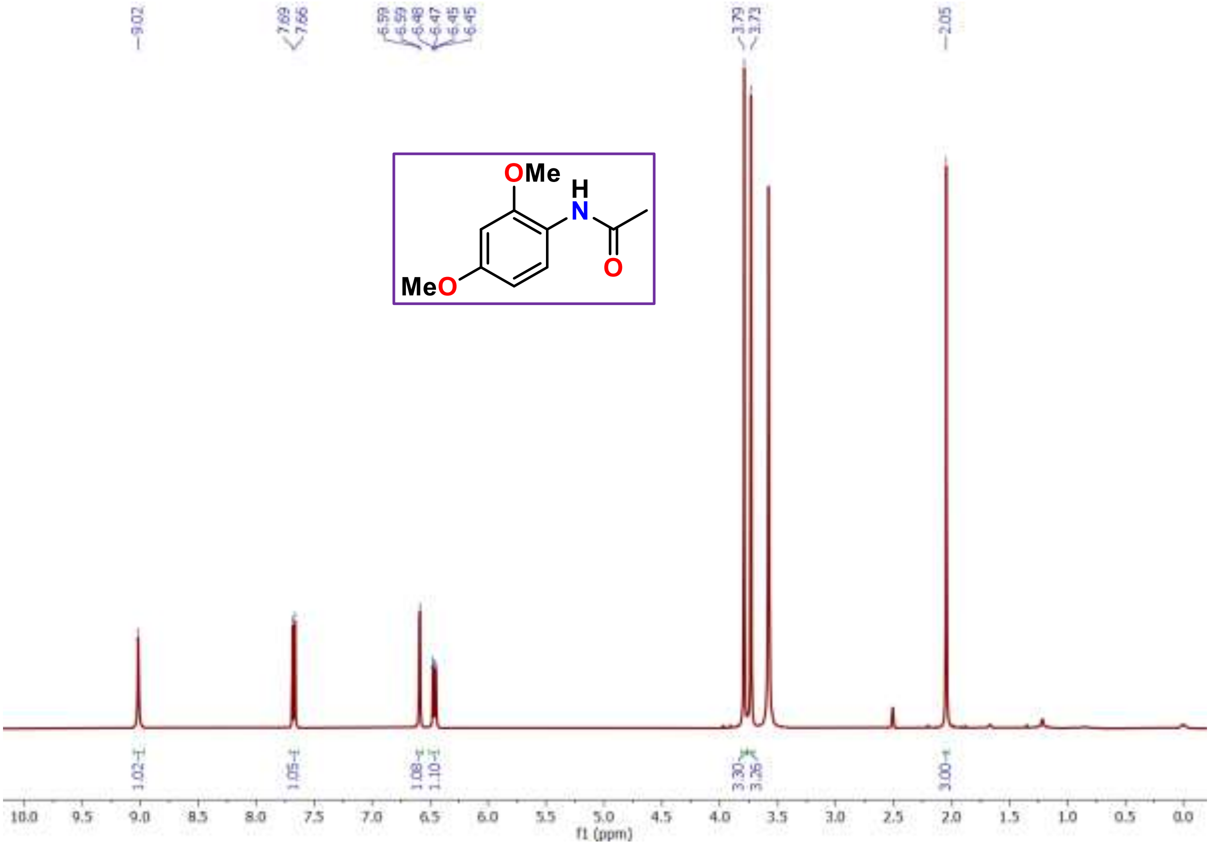
9.02
7.69
7.66
6.59
6.59
6.48
6.47
6.45
6.45
3.79
3.73
2.05
OMe
H
N
O
MeO
1.02
1.05
1.08
1.10
3.30
3.26
3.00
f1 (ppm)

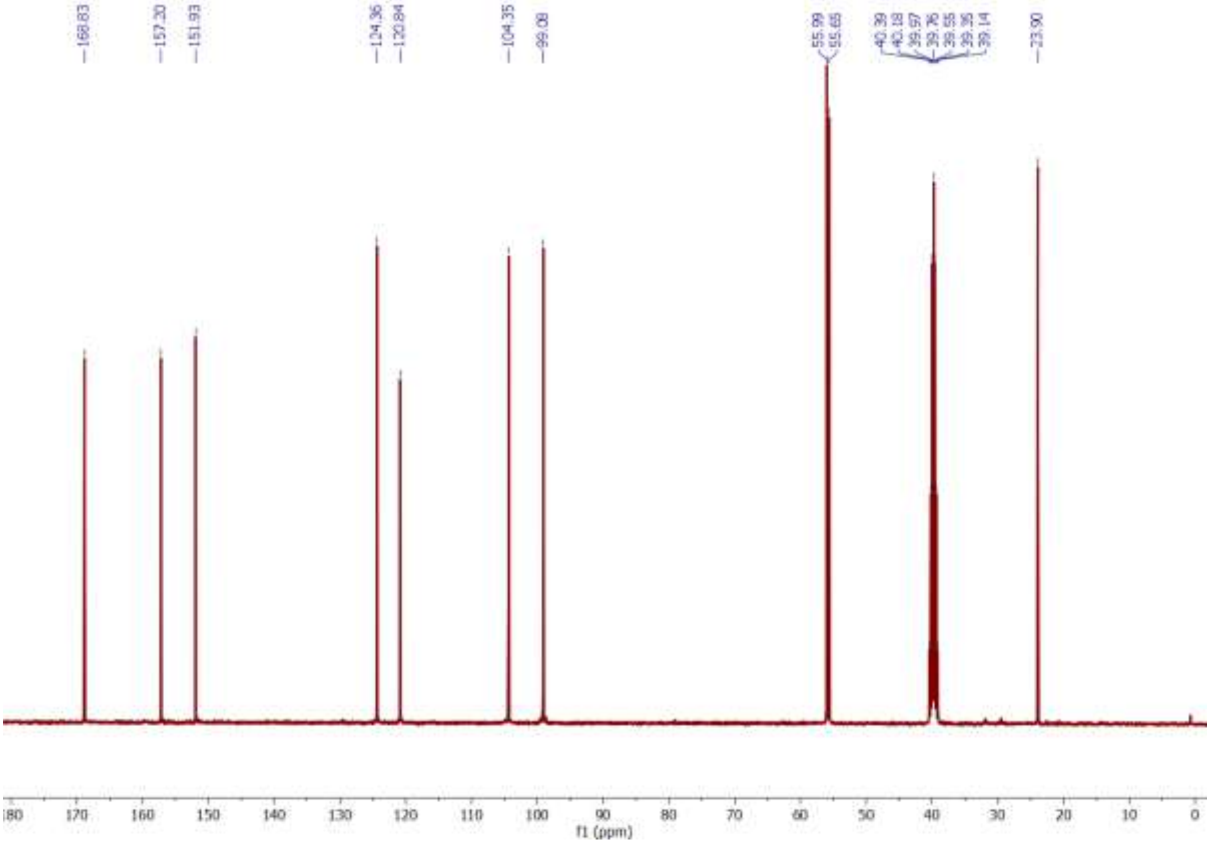
168.83
157.20
151.93
124.36
120.84
104.35
99.08
55.99
55.65
40.39
40.18
39.97
39.76
39.55
39.35
39.14
23.90
f1 (ppm)

Figure S36: ^{1}H and ^{13}C NMR Spectrum of N-(2,4-dimethoxyphenyl)acetamide (**4h**).

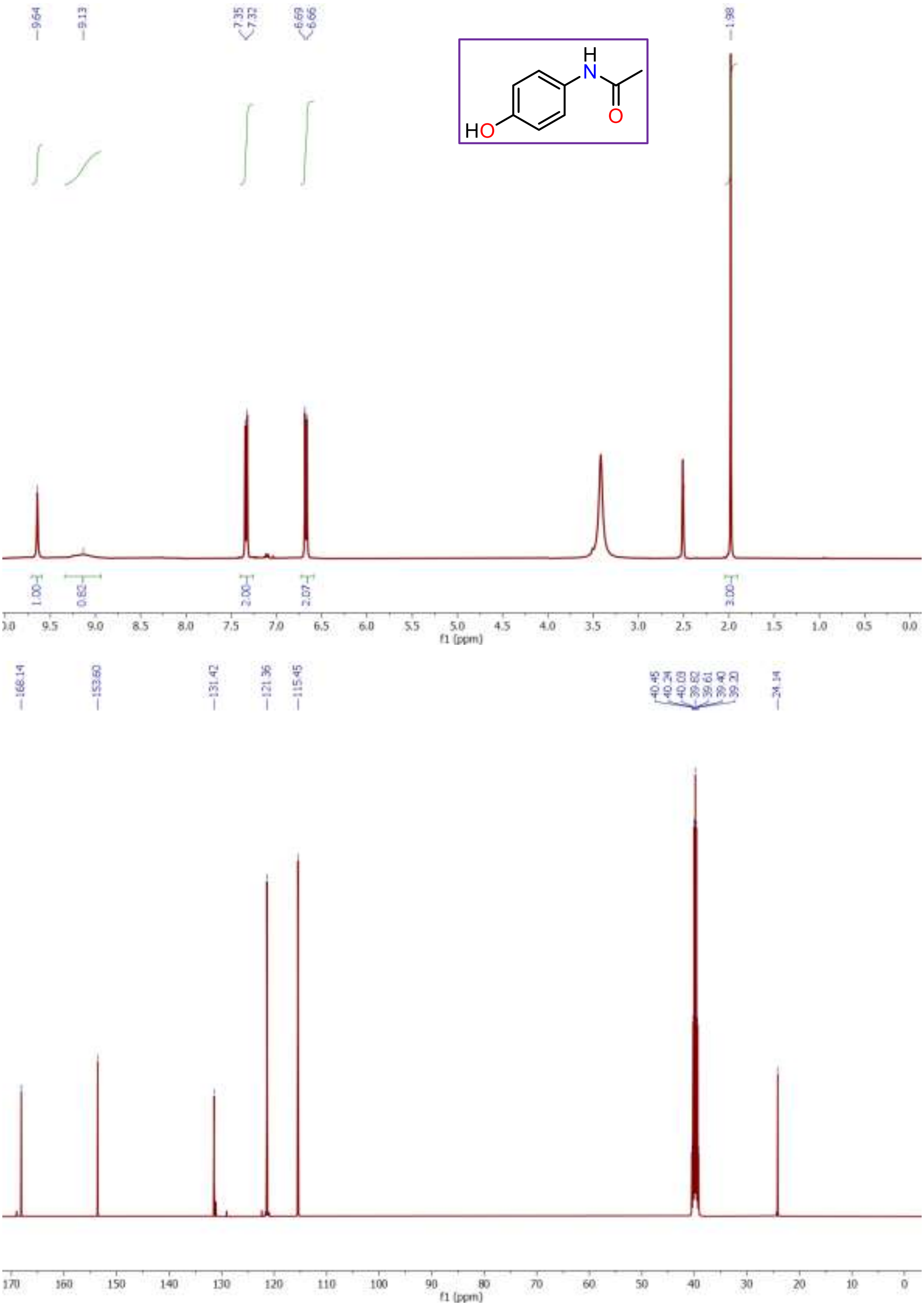

Figure S37: ^{1}H and ^{13}C NMR Spectrum of N-(4-hydroxyphenyl)acetamide (**4i**).

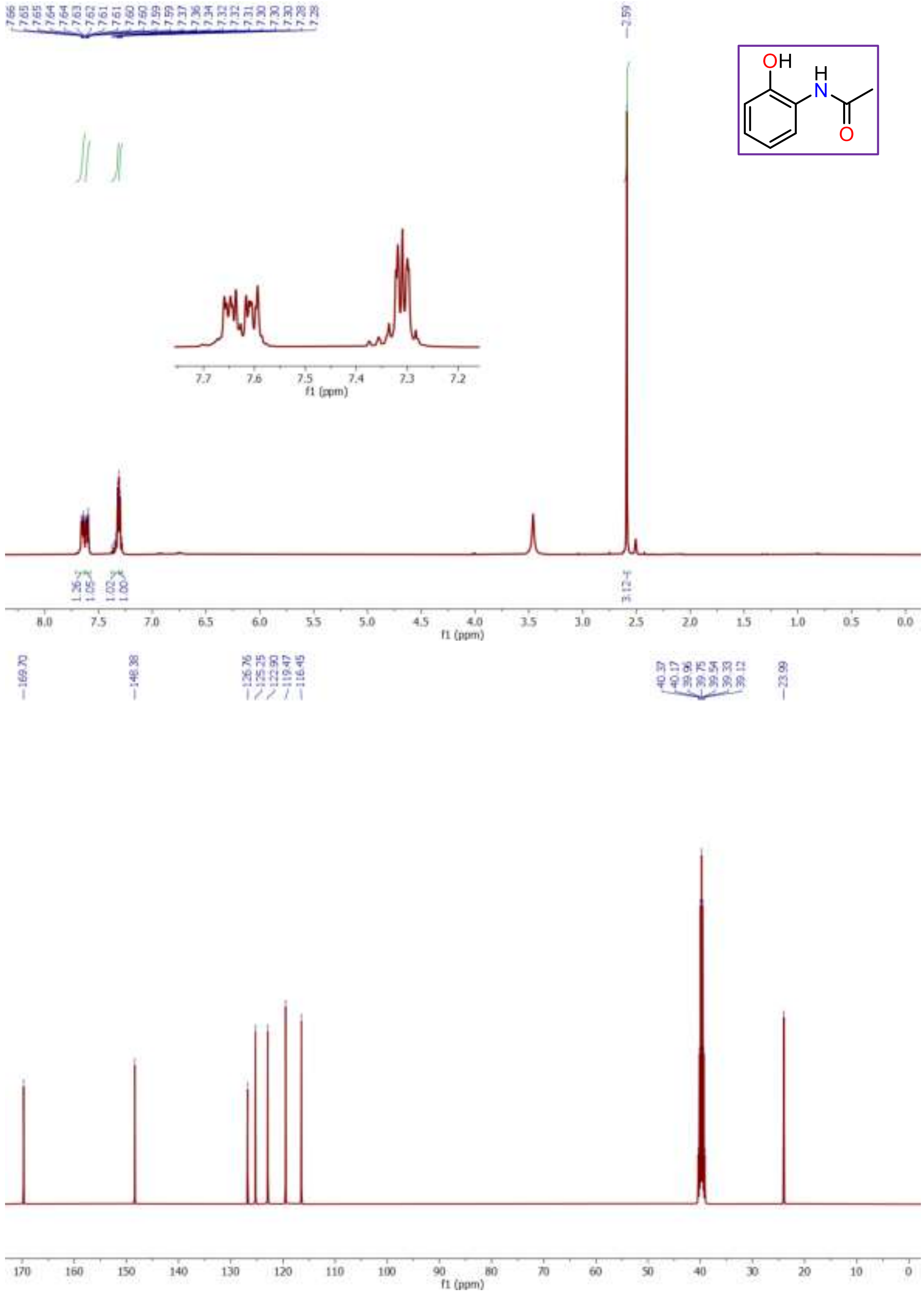

Figure S38: ^{1}H and ^{13}C NMR Spectrum of N-(2-Hydroxyphenyl)acetamide (**4j**).

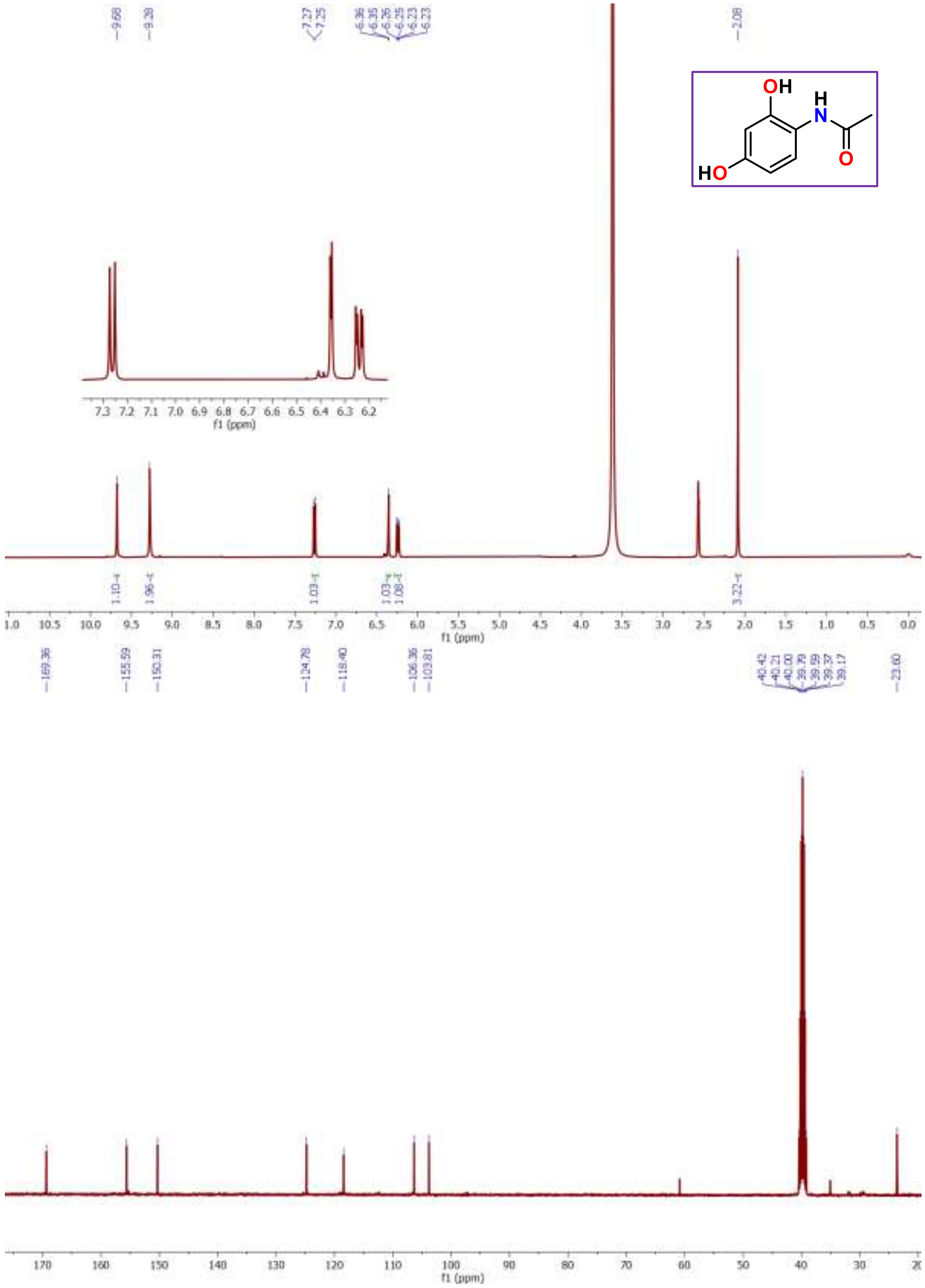

Figure S39: ^{1}H and ^{13}C NMR Spectrum of N-(2,4-dihydroxyphenyl)acetamide (**4k**).

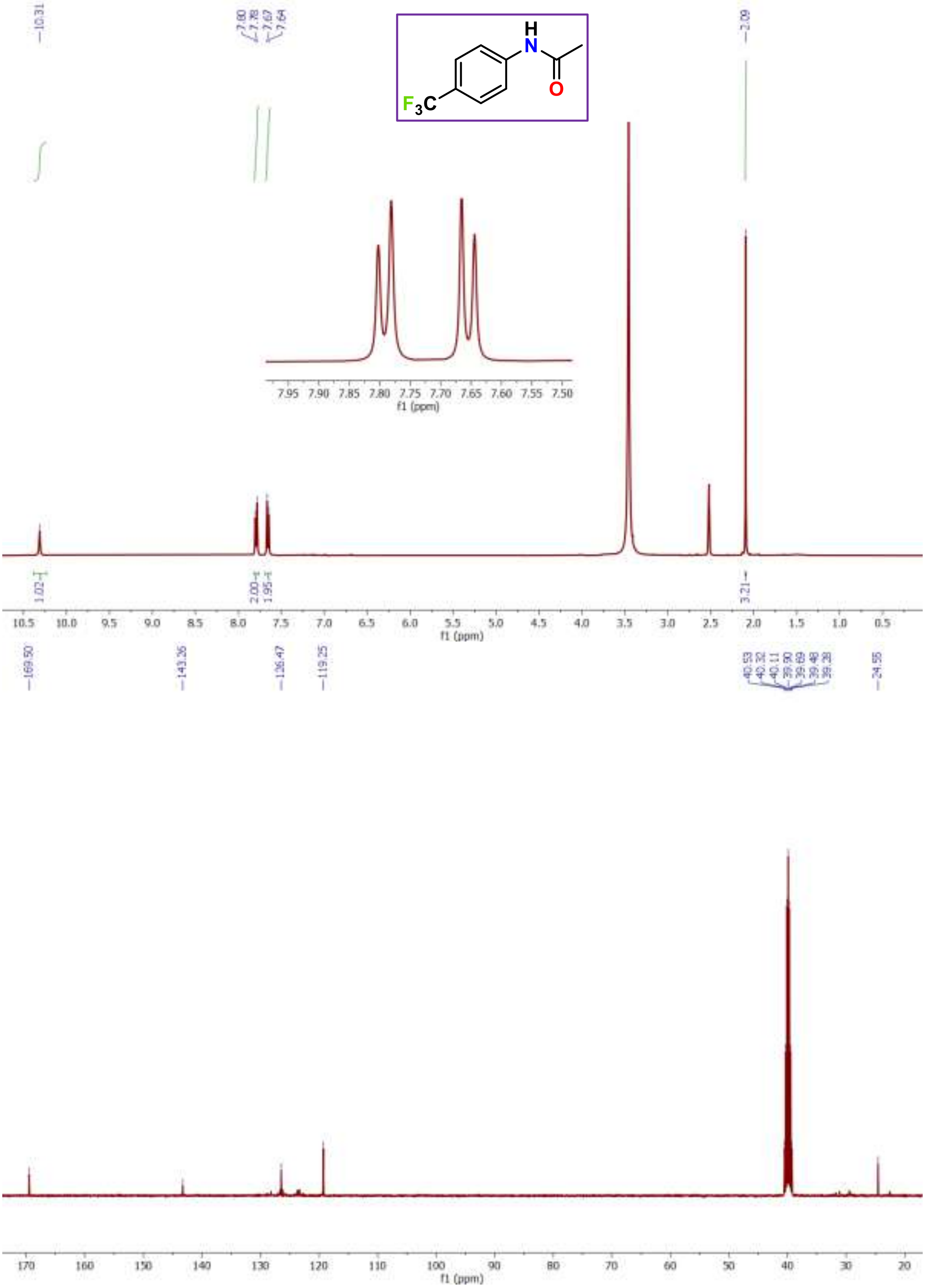

Figure S40: ^{1}H and ^{13}C NMR Spectrum of N-(4-(trifluoromethyl)phenyl)acetamide (**4l**).

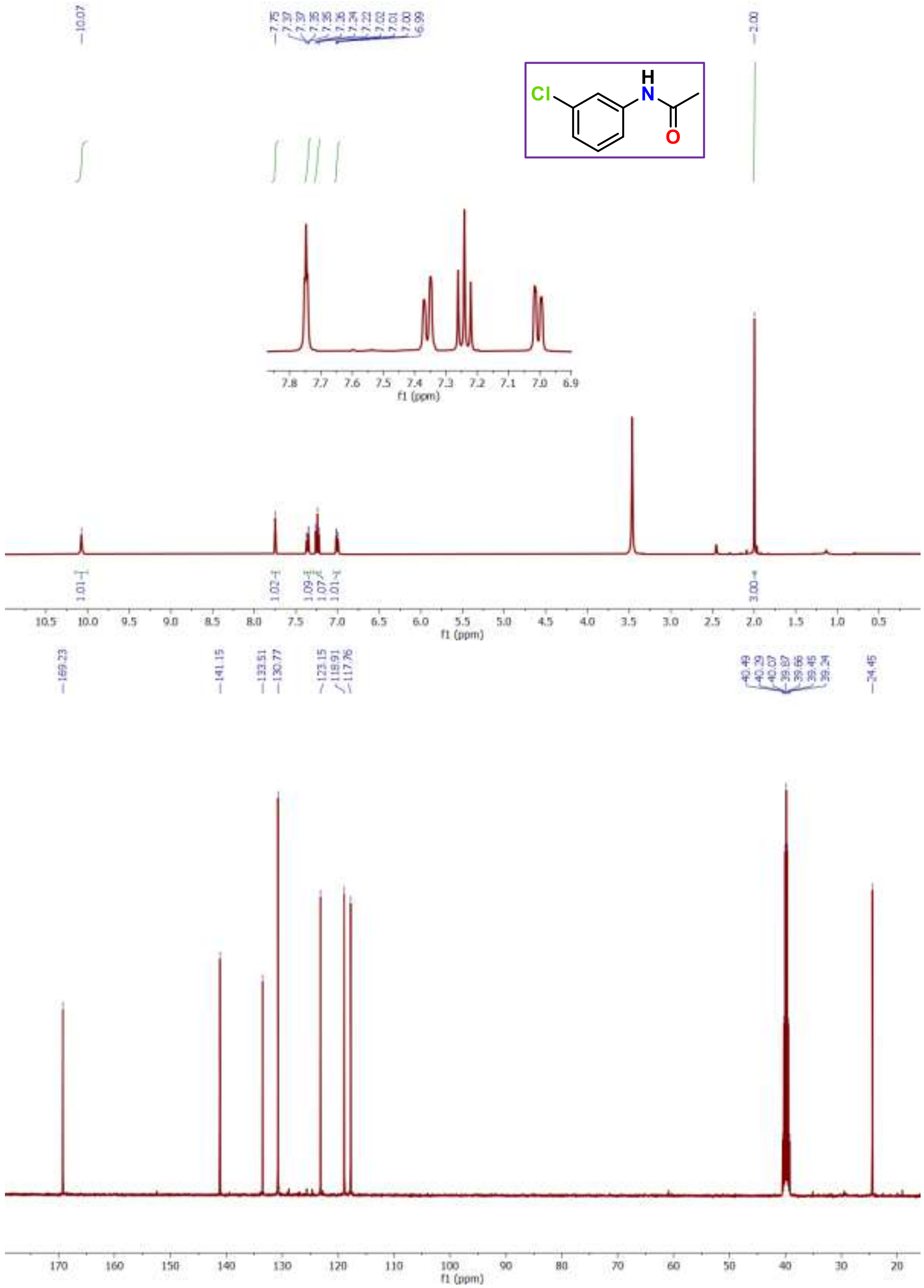

Figure S41: ^{1}H and ^{13}C NMR Spectrum of N-(3-chlorophenyl)acetamide (**4m**).

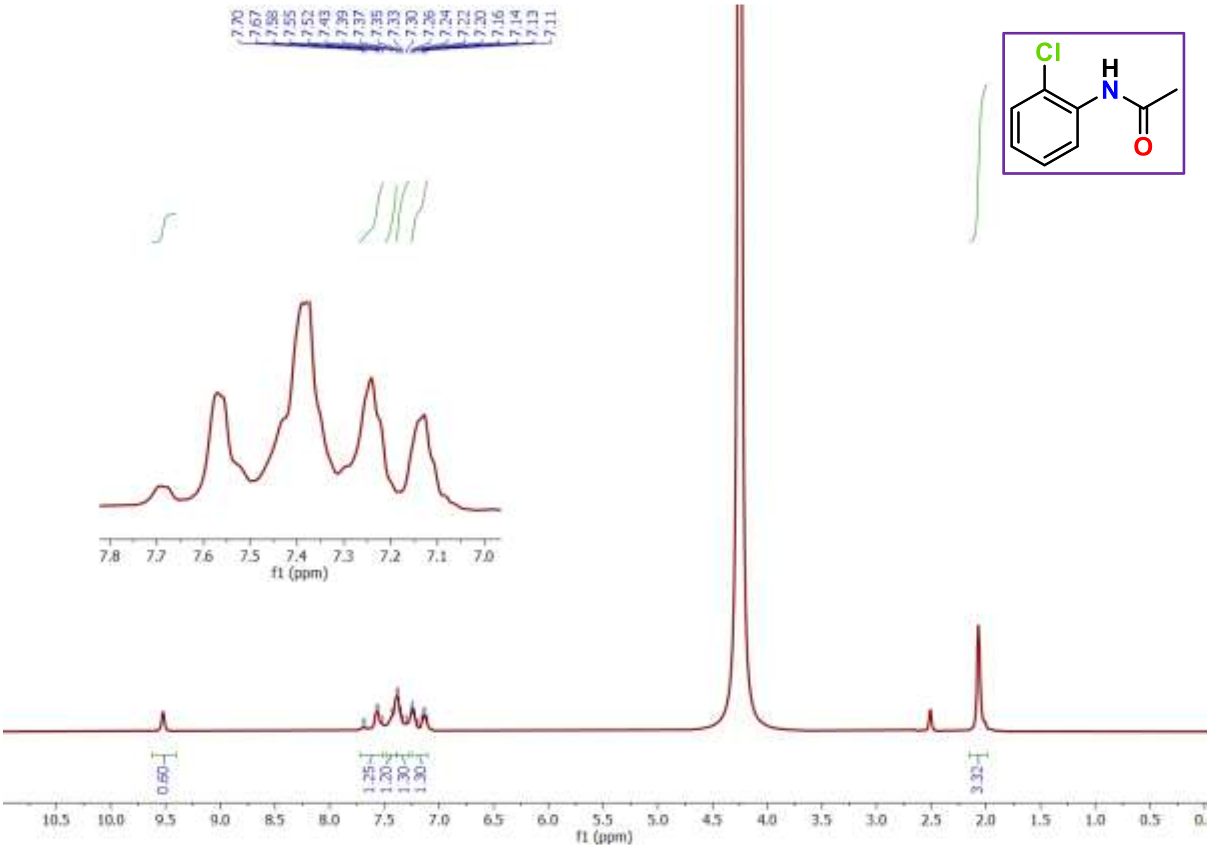

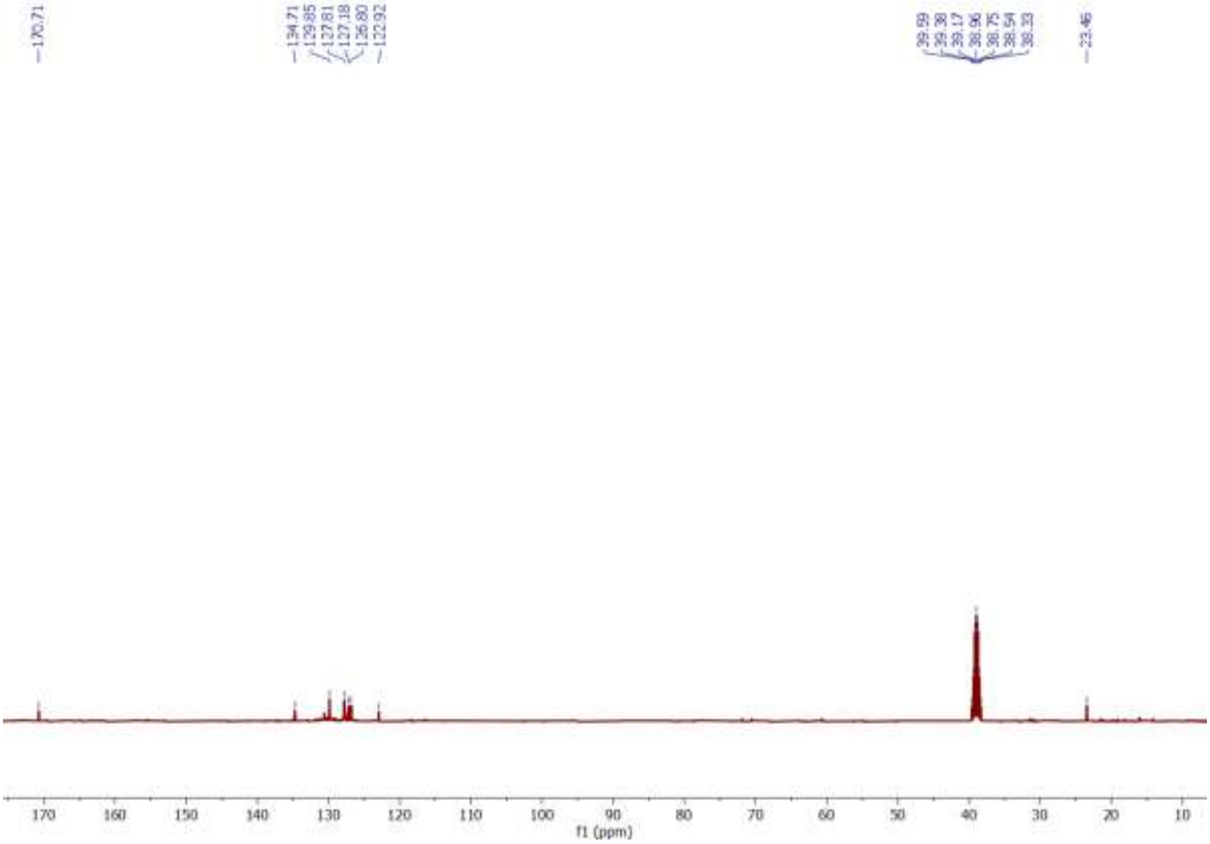

Figure S42: ^{1}H and ^{13}C NMR Spectrum of N-(2-chlorophenyl)acetamide (**4n**).

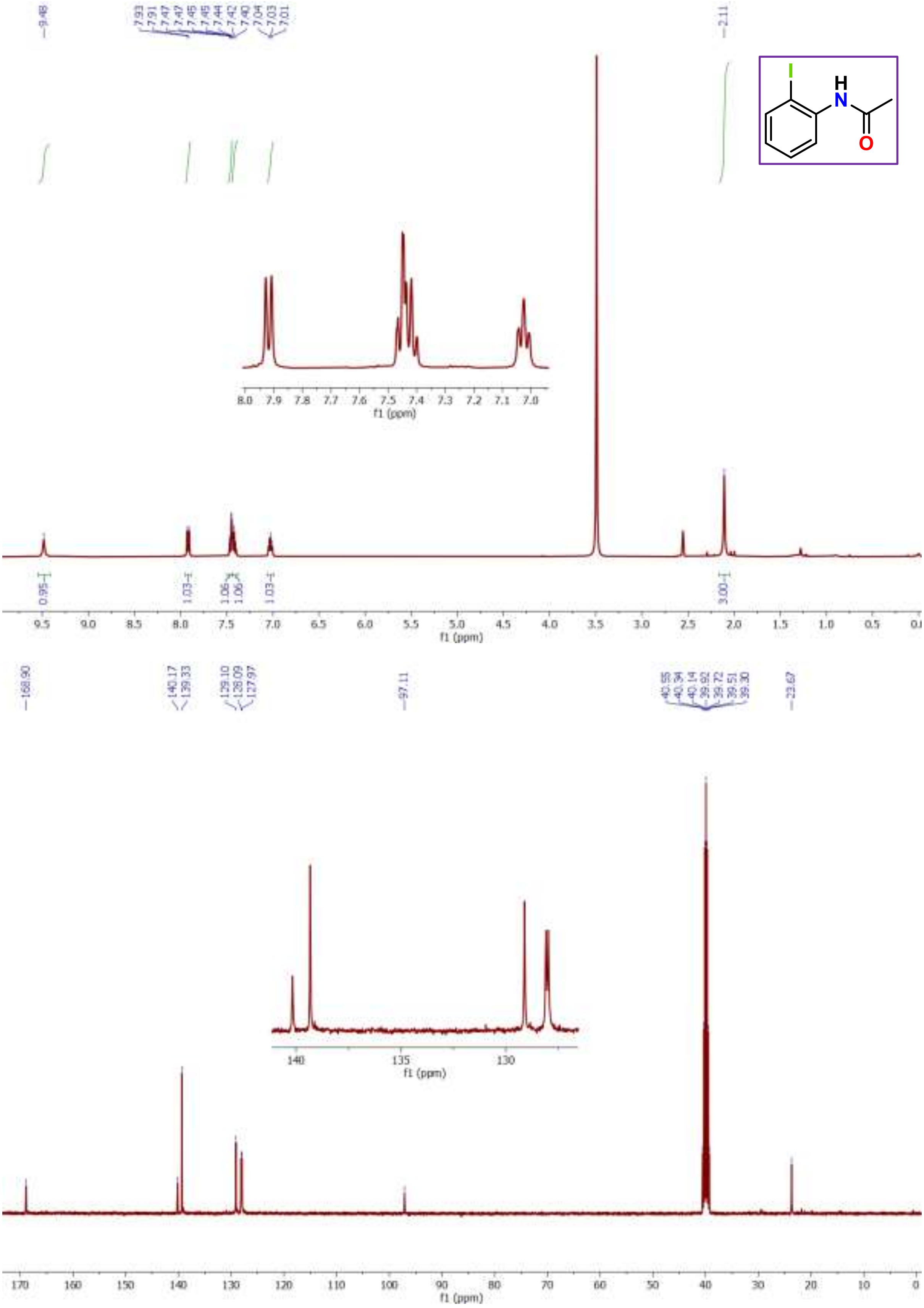

Figure S43: ^{1}H and ^{13}C NMR Spectrum of N-(2-iodophenyl)acetamide (**4o**).

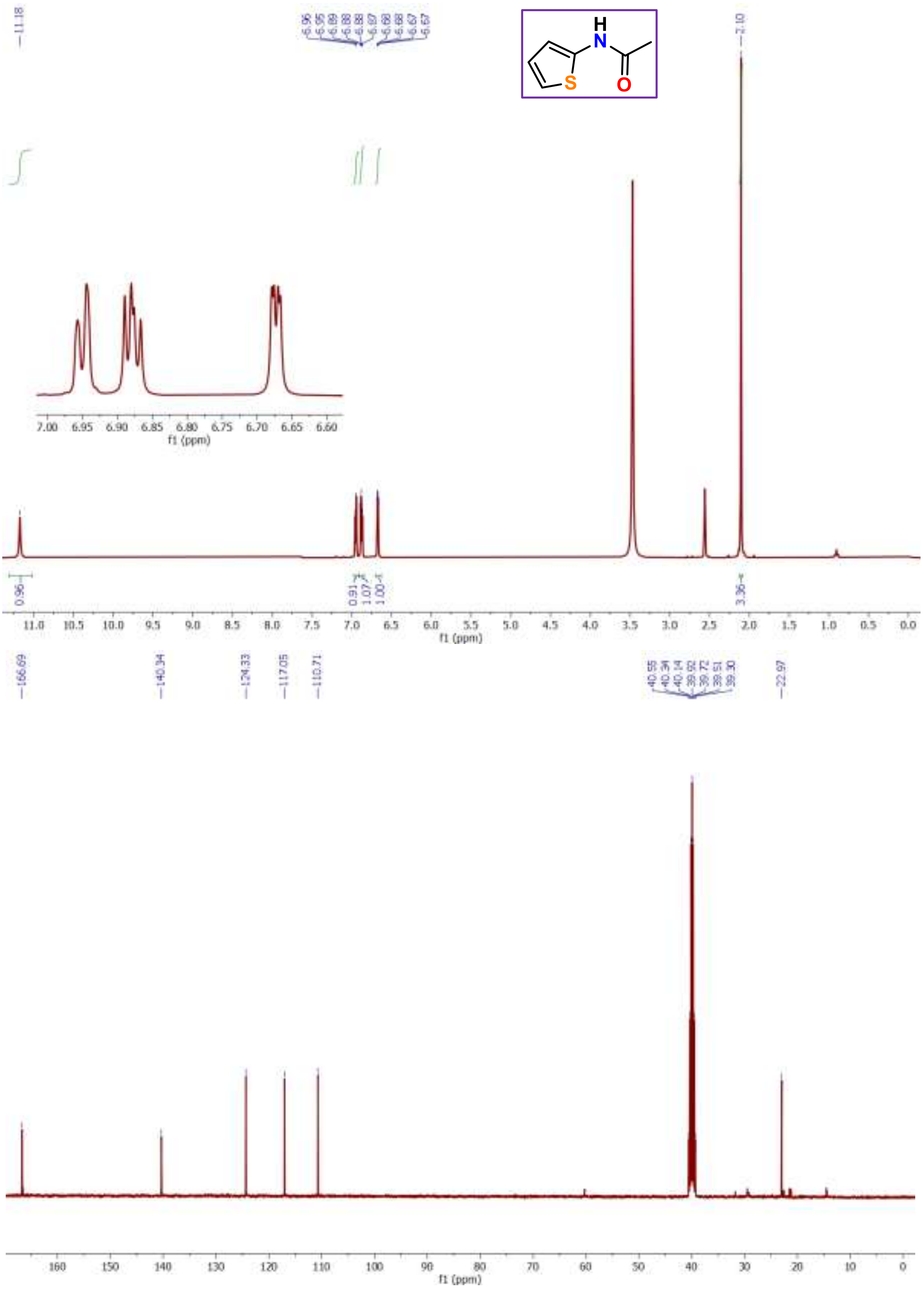

Figure S44: ^{1}H and ^{13}C NMR Spectrum of (N-(thiophen-2-yl)acetamide) (**4p**).

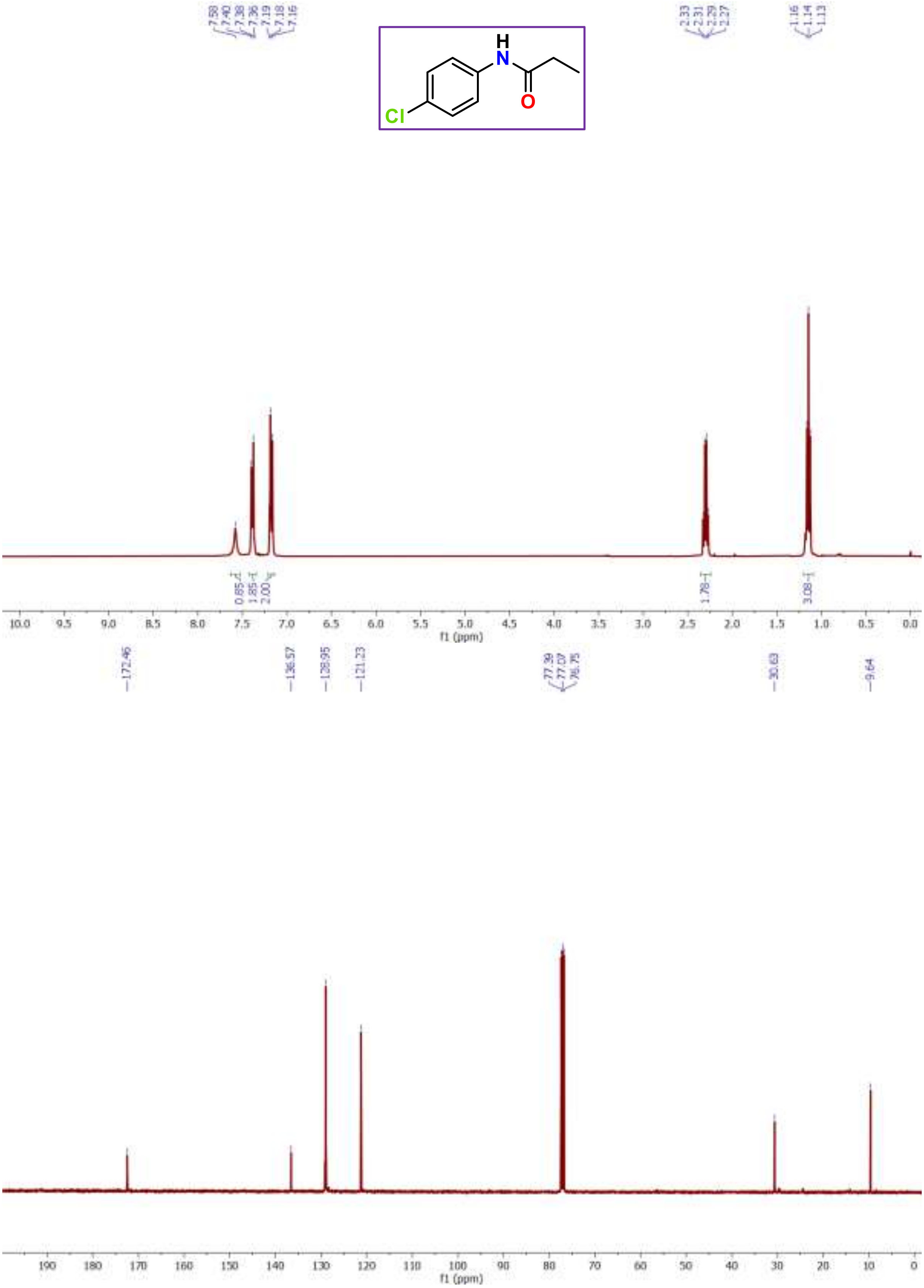

Figure S45: ^{1}H and ^{13}C NMR Spectrum of N-(4-chlorophenyl)propionamide (**4q**).

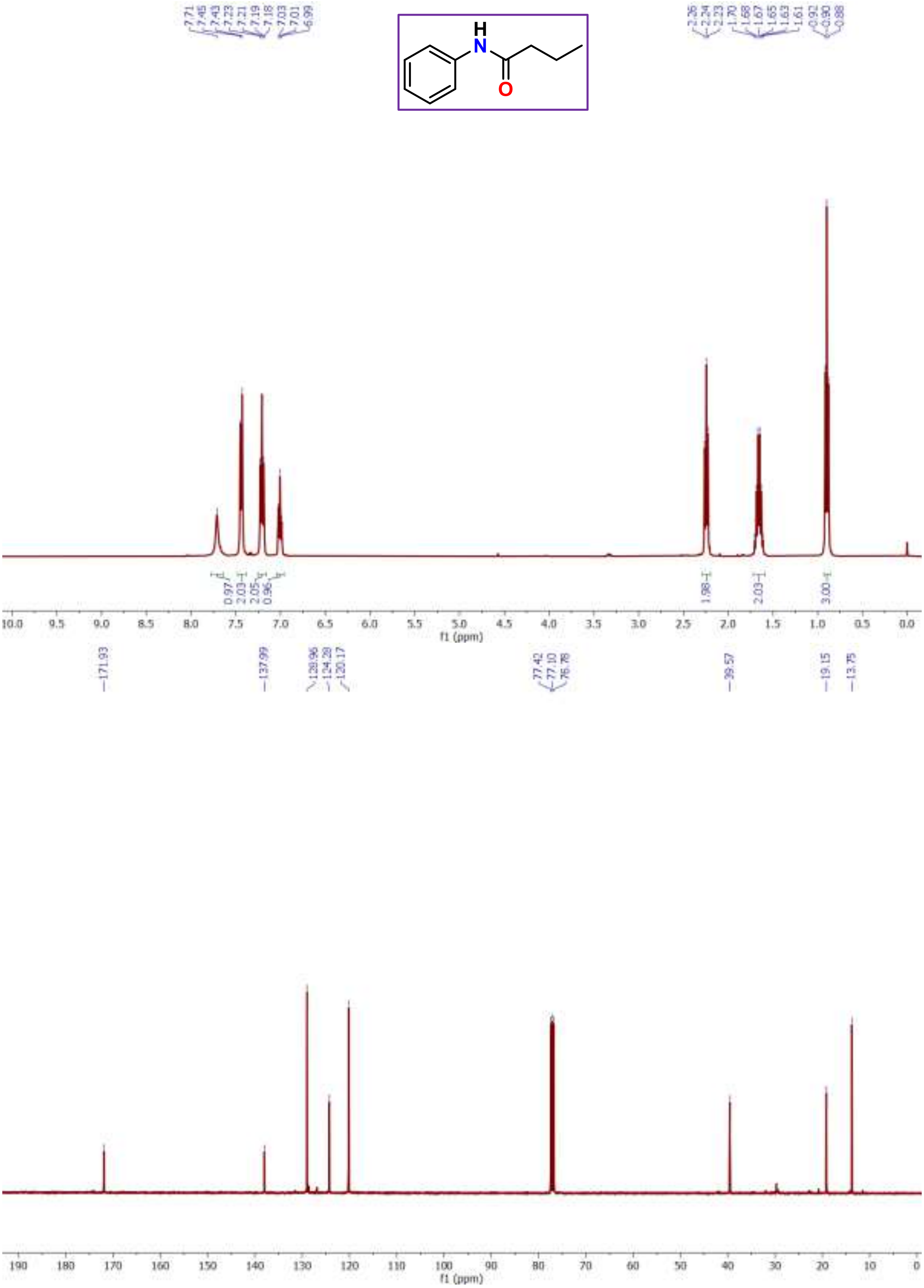

Figure S46: ^{1}H and ^{13}C NMR Spectrum of N-Phenylbutyramide (**4r**).

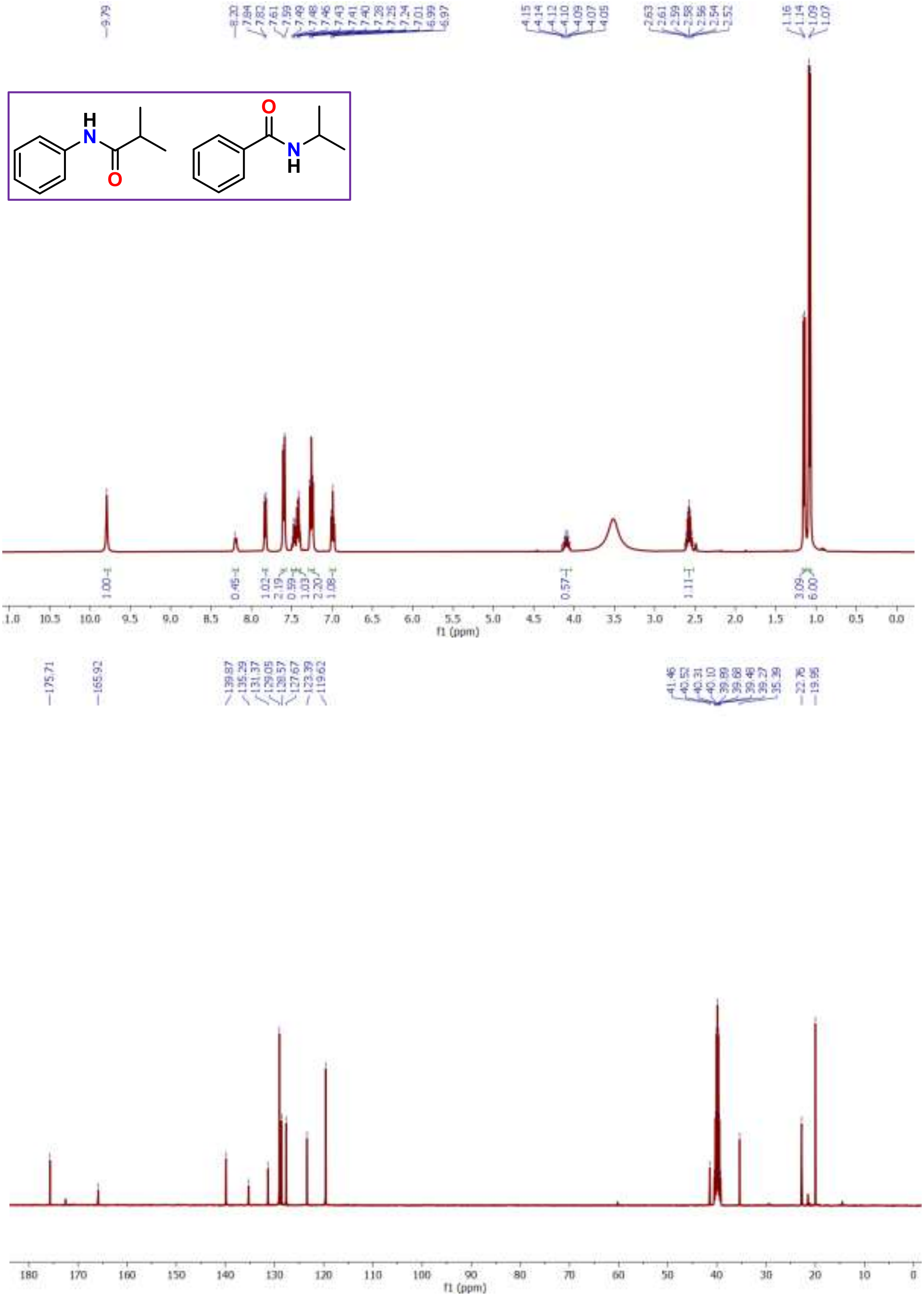

Figure S47: ^{1}H and ^{13}C NMR Spectrum of N-phenylisobutyramide and N-isopropylbenzamide (**4s**).

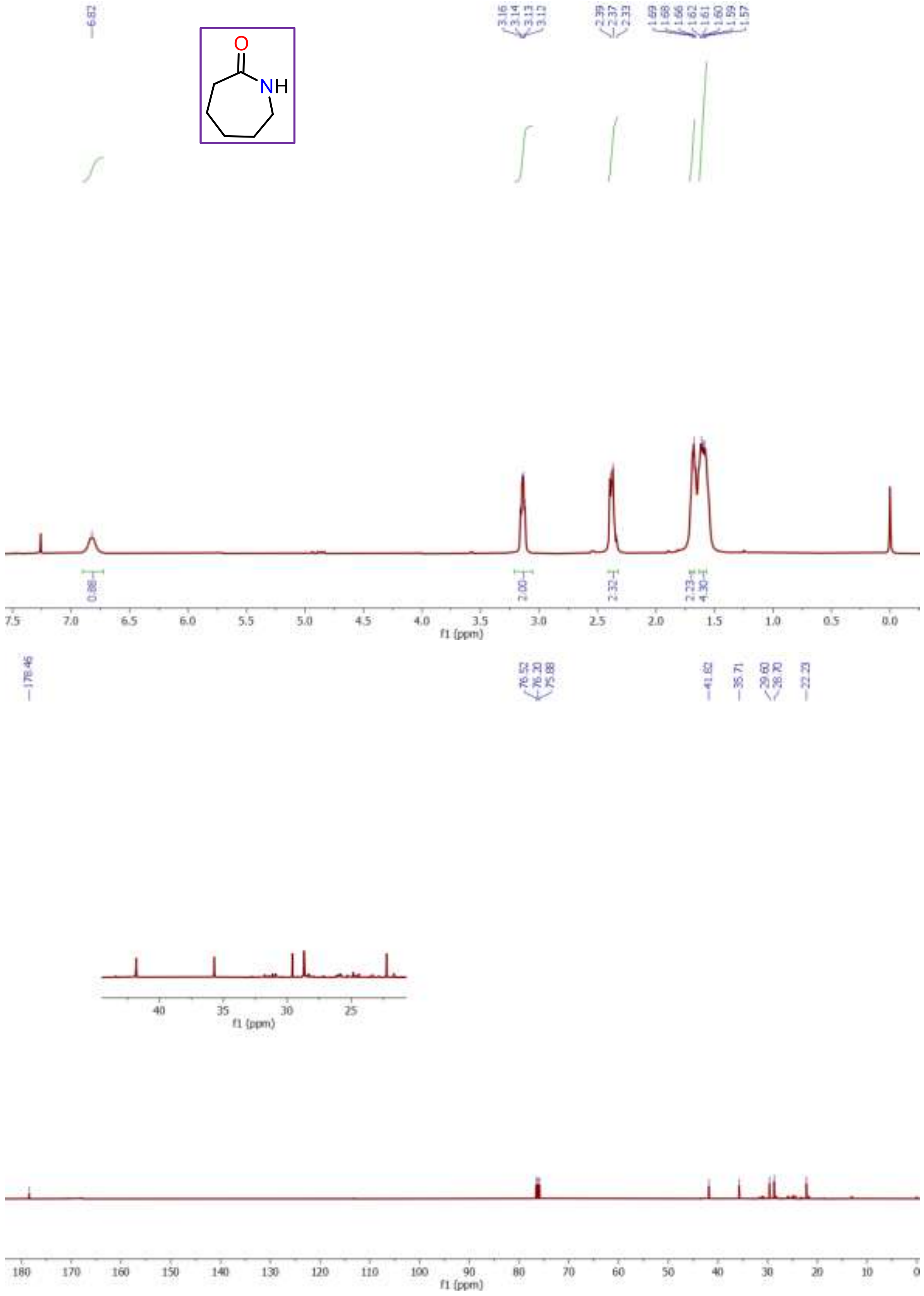

Figure S48: ^{1}H and ^{13}C NMR Spectrum of Azepan-2-one (**4t**).

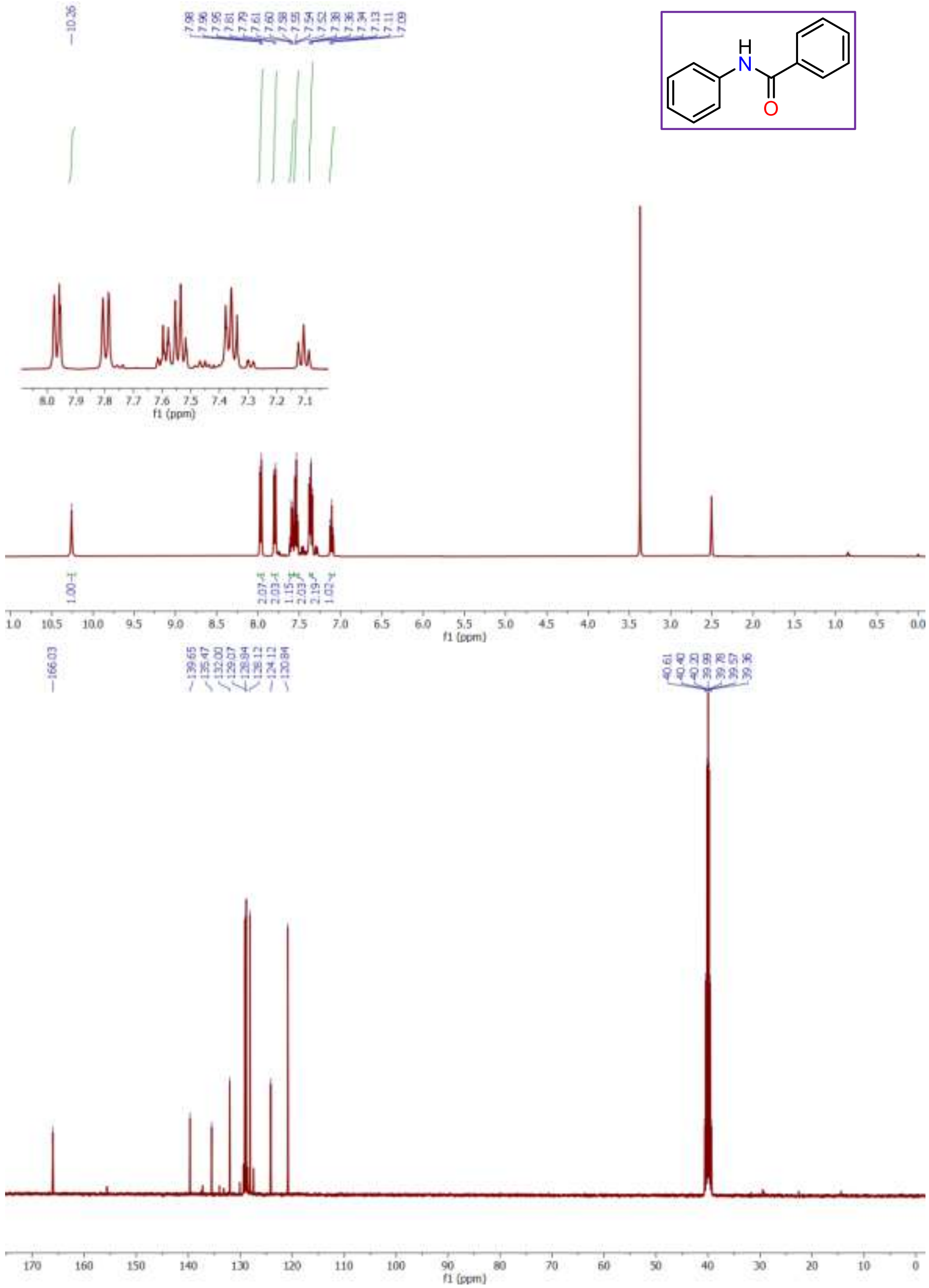

Figure S49: ^{1}H and ^{13}C NMR Spectrum of N-Phenylbenzamide (**4u**).

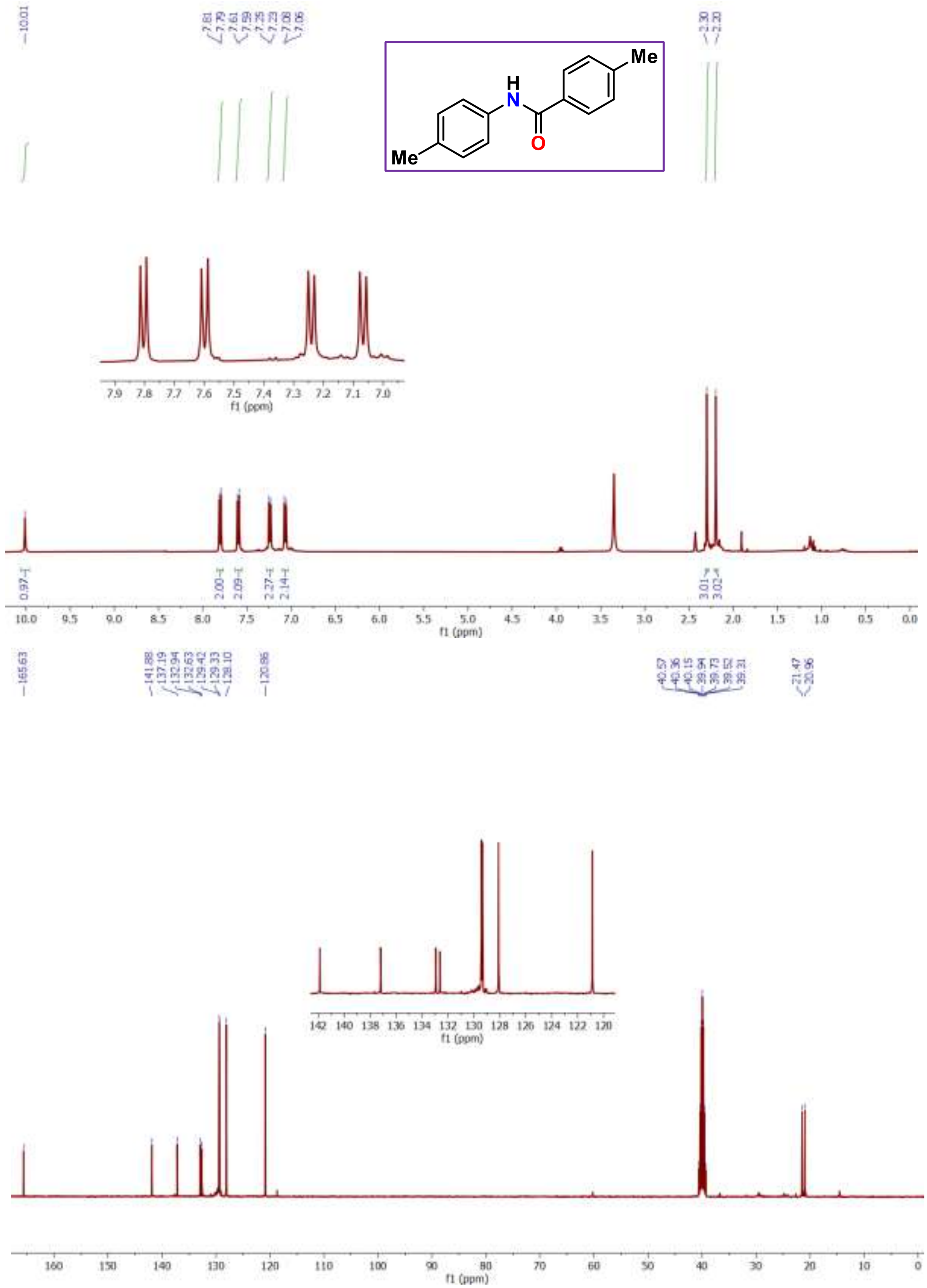

Figure S50: ^{1}H and ^{13}C NMR Spectrum of (4-methyl-*N*-*p*-tolylbenzamide) (**4v**).

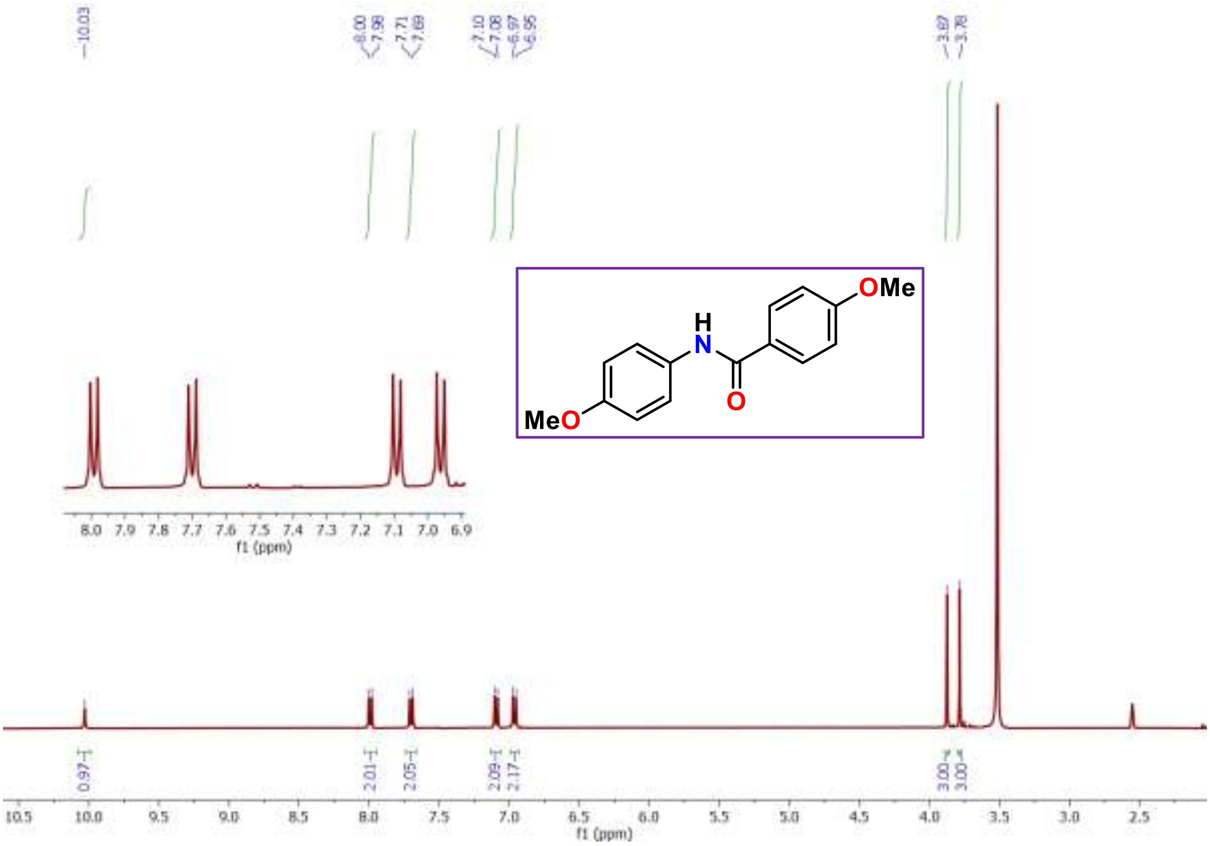

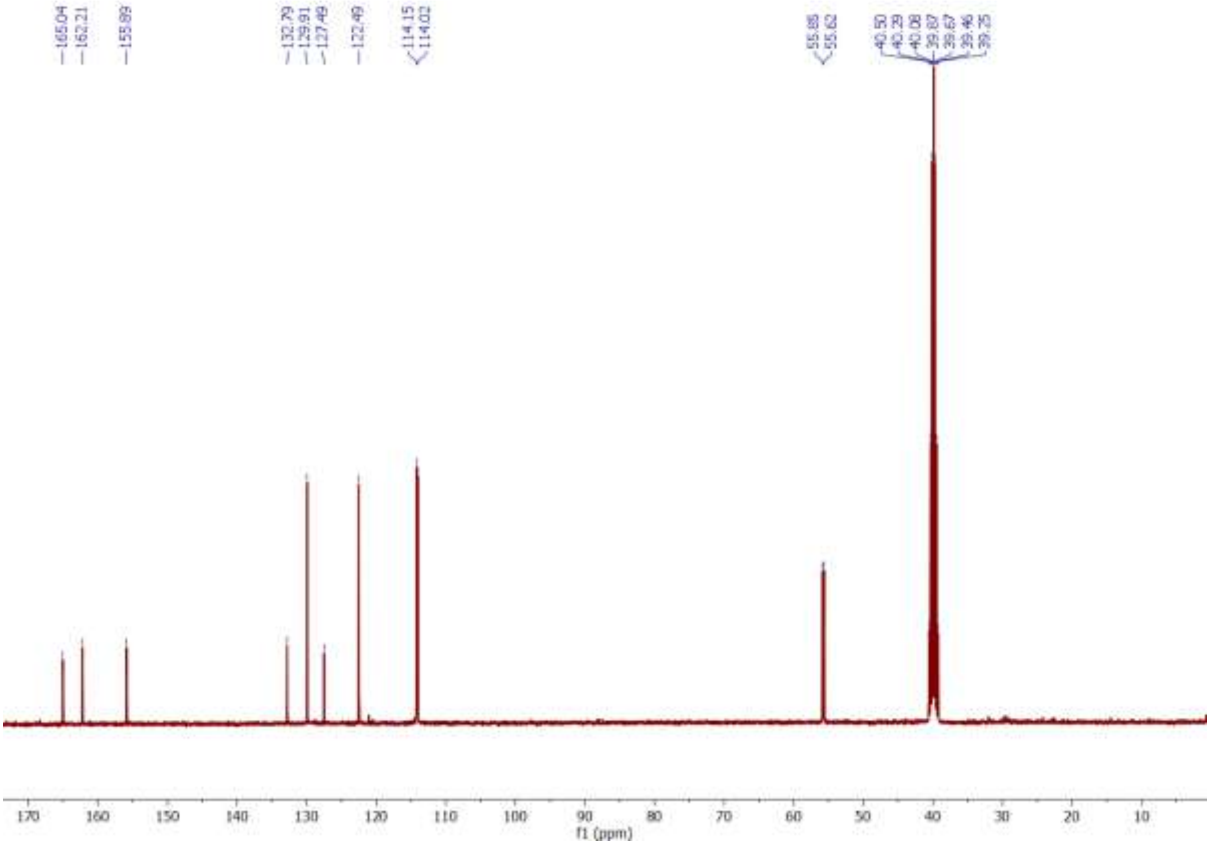

Figure S51: ^{1}H and ^{13}C NMR Spectrum of (4-methyl-*N*-*p*-tolylbenzamide) (**4w**).

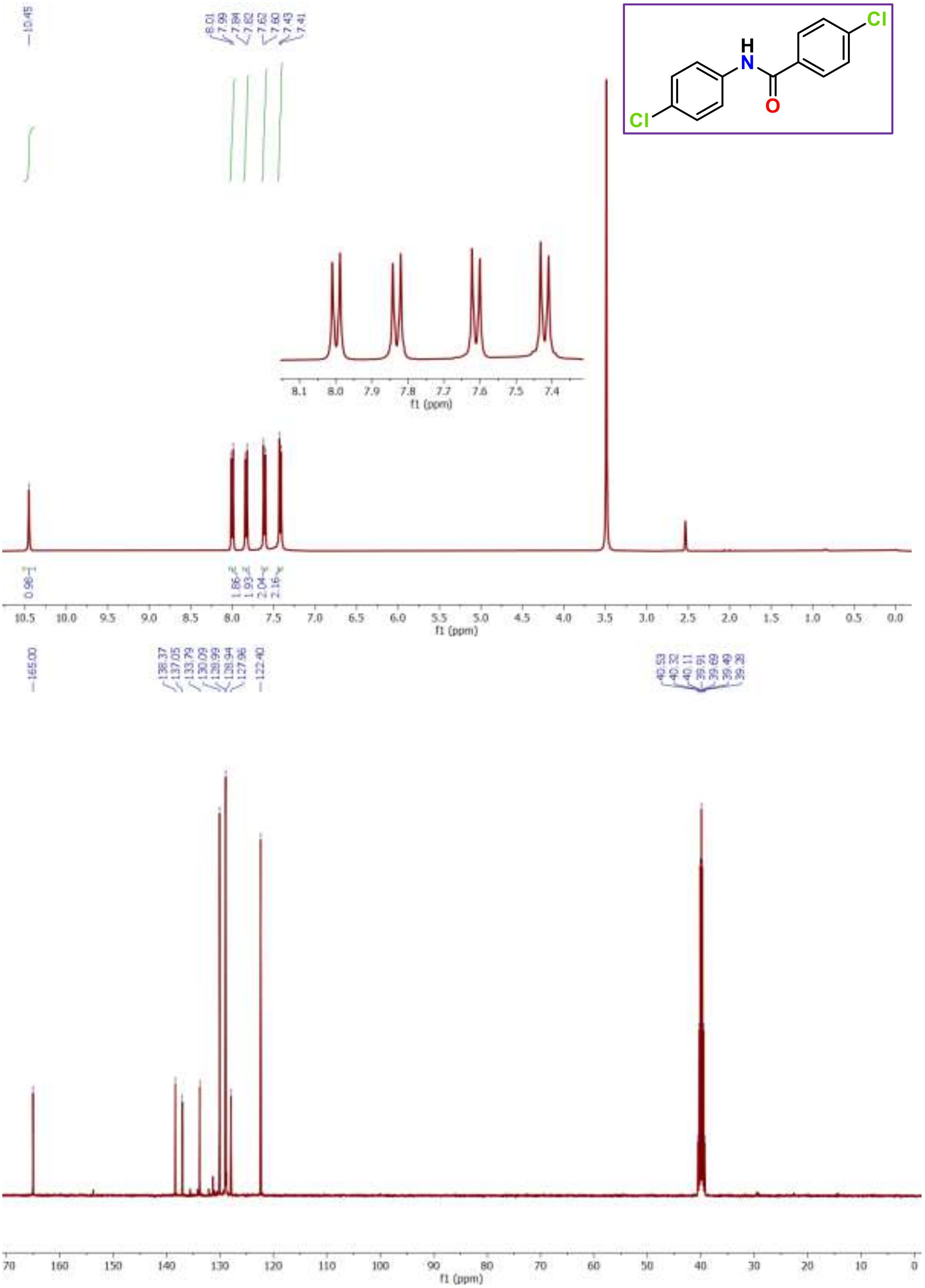

Figure S52: ^{1}H and ^{13}C NMR Spectrum of 4-chloro-N-(4-chlorophenyl)benzamide (**4x**).

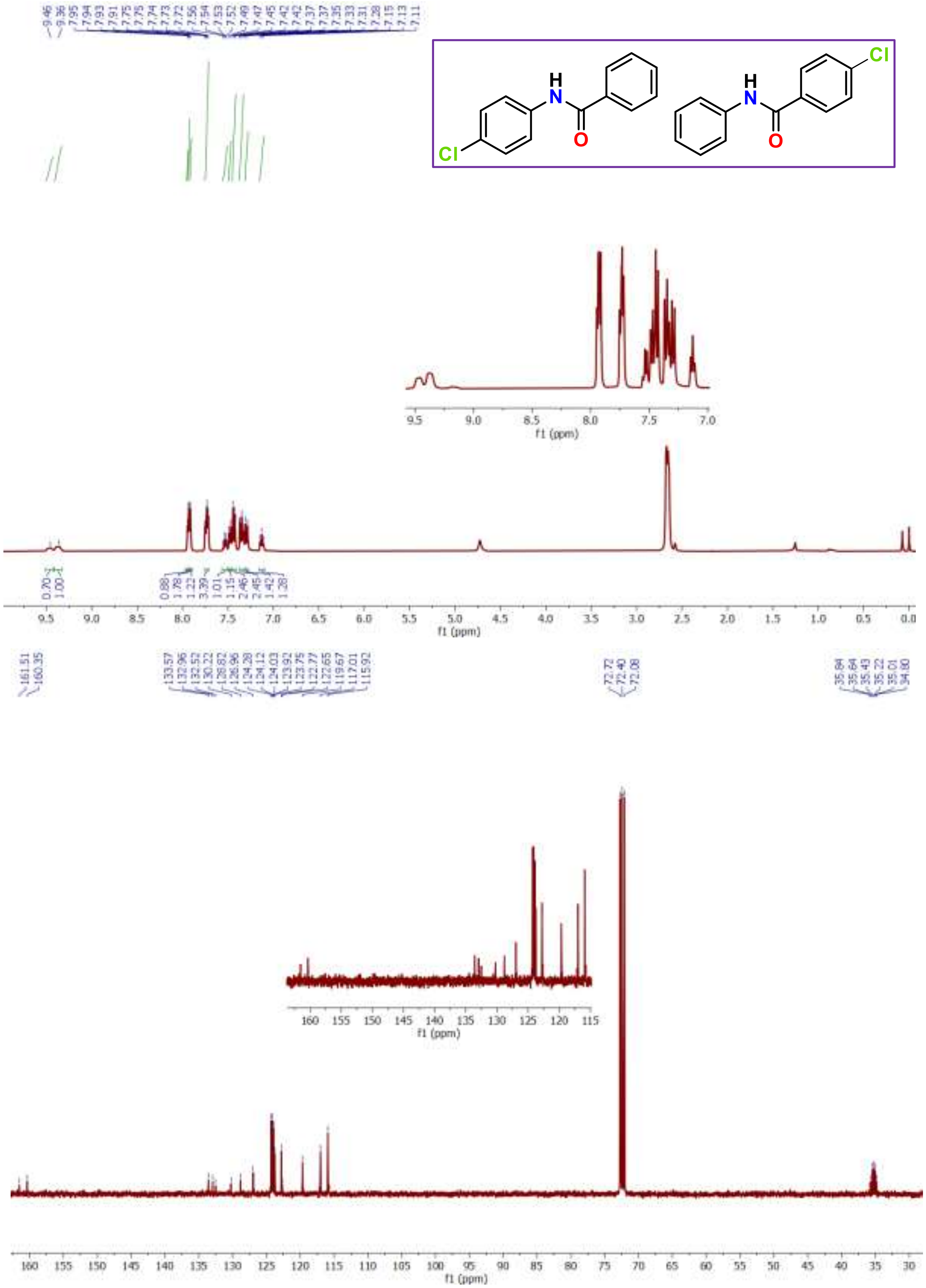

Figure S53: ^{1}H and ^{13}C NMR Spectrum of N-(4-chlorophenyl)benzamide and 4-chloro-N-phenylbenzamide (**4y**).

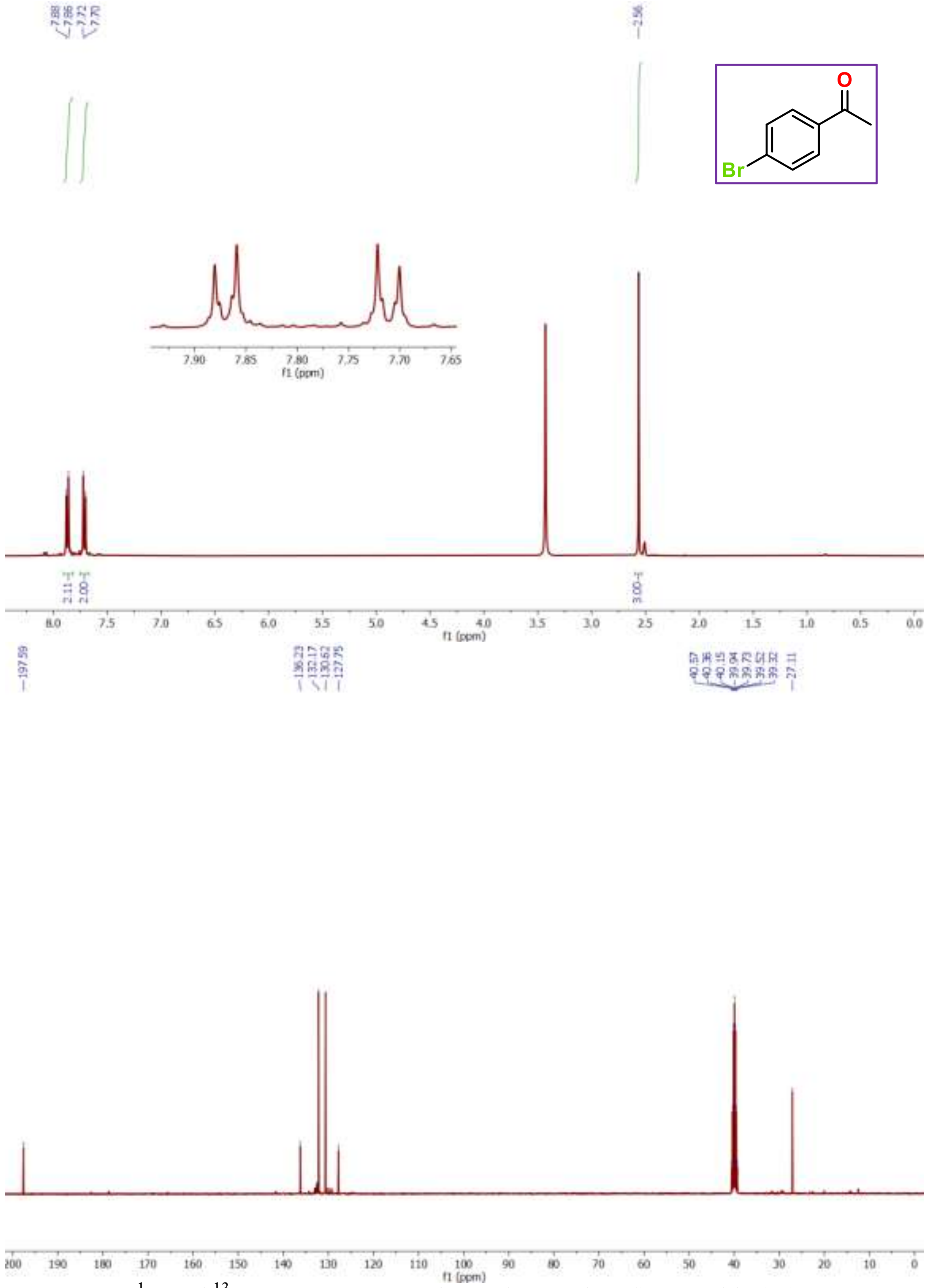

Figure S54: ^{1}H and ^{13}C NMR Spectrum of N-(4-bromophenyl)acetamide (**4z**).

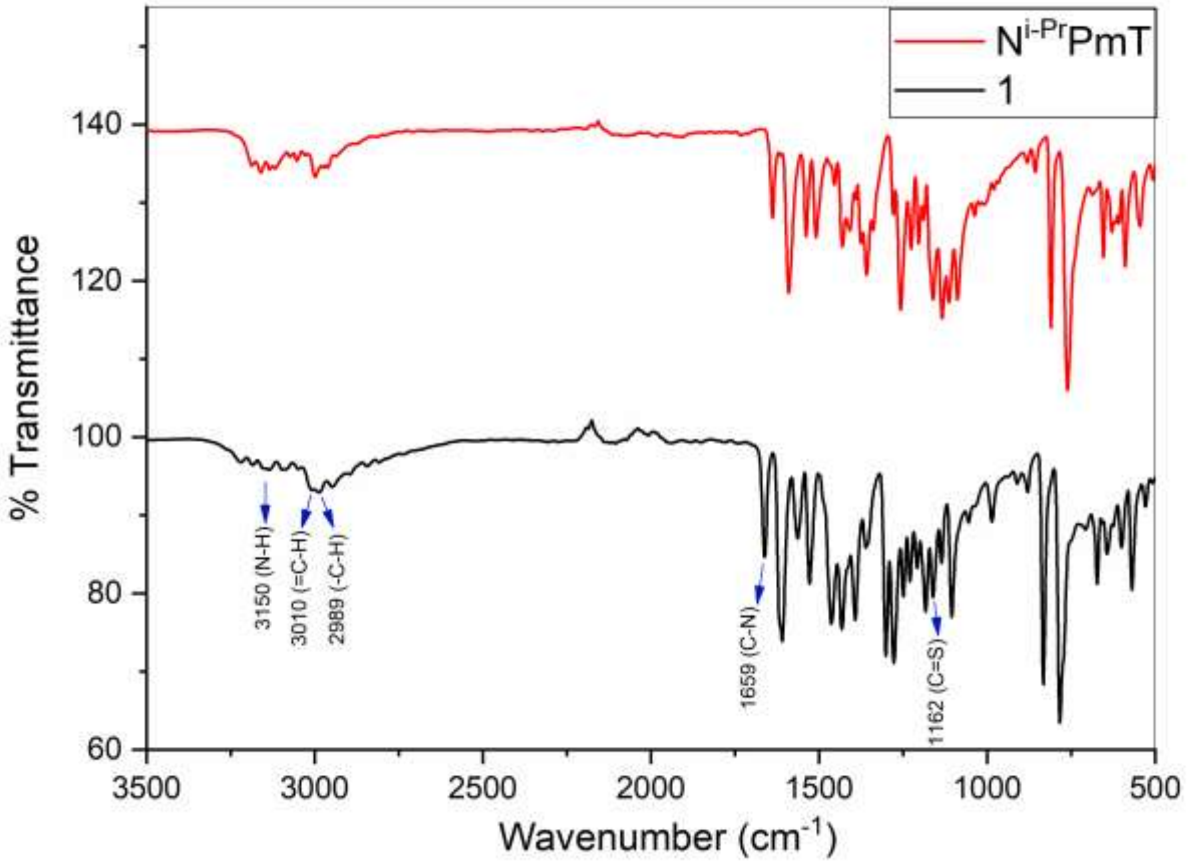

Figure S55: FT-IR spectra of Hg(II) complex (**1**) and Ligand **N$^{i\text{-}Pr}$PmT**.

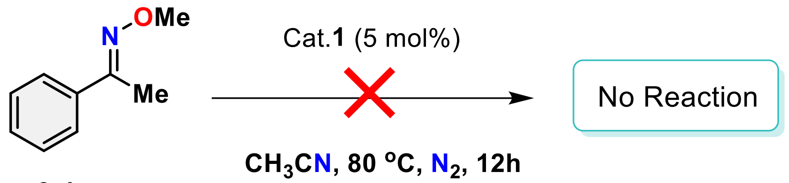

Scheme S1: The Beckmann rearrangement of (E)-1-phenylethan-1-one O-methyl oxime (**3ah**) under standard conditions using Hg(II) catalyst (**1**).
